# Ogt-mediated O-GlcNAcylation inhibits astrocytes activation through modulating NF-κB signaling pathway

**DOI:** 10.1186/s12974-023-02824-8

**Published:** 2023-06-22

**Authors:** Xiaoxue Dong, Liqi Shu, Jinyu Zhang, Xu Yang, Xuejun Cheng, Xingsen Zhao, Wenzheng Qu, Qiang Zhu, Yikai Shou, Guoping Peng, Binggui Sun, Wen Yi, Qiang Shu, Xuekun Li

**Affiliations:** 1 The Children’s Hospital, National Clinical Research Center for Child Health, School of Medicine, Zhejiang University, Hangzhou, 310052 China; 2 The Institute of Translational Medicine, School of Medicine, Zhejiang University, Hangzhou, 310029 China; 3 Department of Neurology, The Warren Alpert Medical School of Brown University, Providence, RI 02908 USA; 4 MOE Key Laboratory of Biosystems Homeostasis & Protection, College of Life Sciences, Zhejiang University, Hangzhou, 310058 China; 5 The First Affiliated Hospital, Zhejiang University School of Medicine, Hangzhou, 310058 China; 6 NHC and CAMS Key Laboratory of Medical Neurobiology, School of Brain Science and Brain Medicine, Zhejiang University, Hangzhou, 310058 Zhejiang China; 7 Zhejiang University Cancer Center, Zhejiang University, Hangzhou, 310029 China; 8 Binjiang Institute of Zhejiang University, Hangzhou, 310053 China

## Abstract

Previous studies have shown that Ogt-mediated O-GlcNAcylation is essential for neuronal development and function. However, the function of O-GlcNAc transferase (Ogt) and O-GlcNAcylation in astrocytes remains largely unknown. Here we show that *Ogt* deficiency induces inflammatory activation of astrocytes in vivo and in vitro, and impairs cognitive function of mice. The restoration of O-GlcNAcylation via GlcNAc supplementation inhibits the activation of astrocytes, inflammation and improves the impaired cognitive function of *Ogt* deficient mice*.* Mechanistically, Ogt interacts with NF-κB p65 and catalyzes the O-GlcNAcylation of NF-κB p65 in astrocytes. *Ogt* deficiency induces the activation of NF-κB signaling pathway by promoting Gsk3β binding. Moreover, *Ogt* depletion induces the activation of astrocytes derived from human induced pluripotent stem cells. The restoration of O-GlcNAcylation inhibits the activation of astrocytes, inflammation and reduces Aβ plaque of AD mice in vitro and in vivo*.* Collectively, our study reveals a critical function of Ogt-mediated O-GlcNAcylation in astrocytes through regulating NF-κB signaling pathway.

**Supplementary Information:**

The online version contains supplementary material available at 10.1186/s12974-023-02824-8.

## Introduction

Astrocytes are abundant glial cells in central nervous system (CNS) and play critical function, such as maintaining CNS homeostasis, regulating synaptic information processing [1, 25, 39, 58]. Astrocytes activation, marked by the up-regulation of glial fibrillary acidic protein (GFAP) [60], presents in some pathological conditions including neurodegenerative diseases [16, 23, 44]. Reactive astrocytes secrete pro-inflammatory cytokines affecting neuronal survival and consequently contribute to neurological diseases such as Alzheimer’s disease, multiple sclerosis and spinal cord injury [6, 10, 22, 45, 46, 54, 71]. Diverse mechanisms, including epigenetic modifications and environmental factors, have been shown regulate the activation of astrocytes [46, 61, 71].

As a post-translational modification, O-GlcNAcylation is catalyzed by O-GlcNAc transferase (Ogt) with UDP-GlcNAc on the serine and threonine residues of proteins; meanwhile, O-GlcNAcylation can be removed by O-GlcNAcase (Oga). The balance between the activities of Ogt and Oga is essential for the dynamic and reversible feature of O-GlcNAcylation. Previous studies have shown that thousands of cytoplasmic and nuclear proteins are modified with O-GlcNAcylation, which is implicated in gene expression, proteasomal degradation, energy metabolism, cellular stress responses, and signal transduction [24, 26, 40, 64, 75].

Previous studies have shown that neuronal Ogt is essential for gene expression, neuronal survival and synaptic development, and that *Ogt* deficiency leads to abnormal feeding behavior and induces neurodegeneration [29, 36, 37, 55, 62, 67]. Specific deletion of *Ogt* in neural stem/progenitor cells alters neurogenesis and impairs cognitive function of mice [7, 9, 72]. Consistently, global level of O-GlcNAcylation decreases along with brain ageing, and its restoration can attenuate cognitive impairment in aged mice [70]. However, the function of O-GlcNAcylation in astrocytes remains largely unknown.

In the present study, we showed that astrocytic *Ogt* deficiency induced activation of astrocytes in vivo and in vitro and impaired cognitive function of mice. The restoration of O-GlcNAcylation by the replenishment of GlcNAc increased the level of UDP-O-GlcNAcylation, inhibited activation of astrocytes, and improved the cognitive function of *Ogt* deficient mice*.* Mechanistically, Ogt interacted with NF-κB p65 and catalyzed the O-GlcNAcylation of NF-κB p65 in astrocytes. *Ogt* deficiency promoted the binding of Gsk3β to NF-κB p65 and led to the activation of NF-κB signaling pathway. The restoration of decreased O-GlcNAcylation inhibited activation of astrocytes, inflammation and reduced Aβ plaque in the brain of Alzheimer’s disease (AD) mouse model*.*

## Results

### *Ogt* deficiency leads to the activation of astrocytes in vitro and in vivo

To determine the function of Ogt in astrocytes, we first isolated astrocytes from the brains of wild-type (WT) adult (postnatal 7-week) mice and performed immunostaining with multiple cell lineage markers. We observed that nearly 97% of cells were positive for astrocyte markers GFAP, Glast and Aldh1l1, respectively, but only very few cells were positive for the neuronal cell marker Tuj1 and the microglia marker Iba1 (Additional file 1: Fig. S1a, b), suggesting the homogeneity of cultured astrocytes. Western blot (WB) assay results showed that astrocytes displayed lower levels of Ogt and Oga compared to neurons (Additional file 1: Fig. S1c–e). However, the ratio of Ogt/Oga was significantly higher in astrocytes than that of neurons (Additional file 1: Fig. S1c, f). Consistently, the level of O-GlcNAcylation was significantly higher in astrocytes compared to neurons (Additional file 1: Fig. S1c, g).

**Fig. 1:**
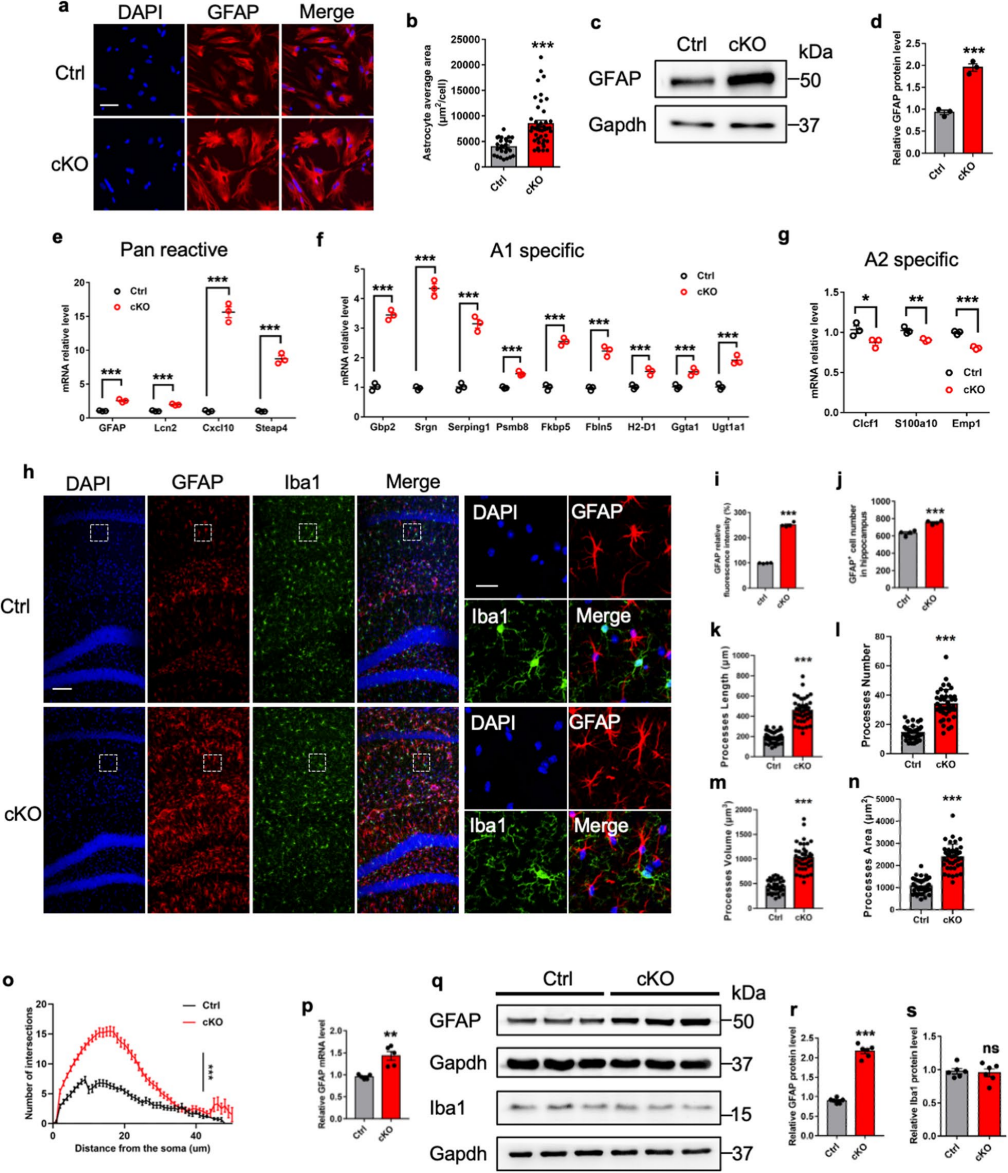
*Ogt* deficiency leads to activation of astrocytes in vitro and in vivo. **a**, **b** Representative images of GFAP immunostaining (**a**) and quantification results showed that *Ogt* deficiency increased the average area of adult astrocytes (**b**). Scale bar, 50 μm. n = 10 astrocytes were picked up per animal and 30 cells in total were analyzed per group. Values represent mean ± SEM; *p < 0.05, **p < 0.01, ***p < 0.001; unpaired Student’s t-test. **c**, **d** Western blot (WB) assay (**c**) and quantification results (**d**) showed that the protein level of GFAP significantly increased in cKO astrocytes compared with Ctrl astrocytes. Astrocytes isolated from 2 to 3 adult mice were pooled together and regarded as n = 1 in the present study. n = 3 independent experiments. Values represent mean ± SEM; *p < 0.05, **p < 0.01, ***p < 0.001; unpaired Student’s t-test. All the original images of western blot assays can be found in Additional file 10: Fig. S10. **e**–**g** qRT-PCR results show that mRNA levels of pan reactive (**e**) and A1 specific (**f**) markers increased in *Ogt* deficient adult astrocytes, and A2 specific markers decreased (**g**). n = 3 independent experiments. Values represent mean ± SEM; *p < 0.05, **p < 0.01, ***p < 0.001; unpaired Student’s t-test. **h** Representative images of GFAP and Iba1 immunostaining. Scale bar, 100 μm. The right panels showed the higher magnification of the area marked with white frame in left panel images. Scale bar (right panels, 20 μm). **i**–**l** Quantification results showed that the level of GFAP fluorescence intensity (**i**) and the number of GFAP^+^ cells (**j**) significantly increased in the hippocampus region of cKO mice compared to Ctrl mice. n = 4 mice per genotype. Values represent mean ± SEM; *p < 0.05, **p < 0.01, ***p < 0.001; unpaired Student’s t-test. **k**–**n** three-dimension (3D) analysis of astrocytic morphology showed the increase of the total length (**k**), the number of processes (**l**), the process volume (**m**) and total process area (**n**) of astrocytes from the hippocampus of adult cKO mice compared with Ctrl mice. n = 10 astrocytes were picked up per animal and 40 cells in total were analyzed per group. Values represent mean ± SEM; *p < 0.05, **p < 0.01, ***p < 0.001; unpaired Student’s t-test. **o** 3D Sholl analysis showed the increased process arbor complexity of astrocytes in the hippocampus of cKO mice compared with Ctrl mice. n = 10 astrocytes were picked up per animal and 40 cells in total were analyzed per group. Values represent mean ± SEM; *p < 0.05, **p < 0.01, ***p < 0.001; two-way ANOVA followed by Sidak’s multiple comparisons test, *F*(_1, 5304)_ = 2258. **p** qRT-PCR results showed that mRNA level of GFAP increased in the hippocampal tissues of cKO mice compared to Ctrl mice. n = 5 mice per genotype. Values represent mean ± SEM; *p < 0.05, **p < 0.01, ***p < 0.001; unpaired Student’s t-test. **q**–**s** WB assay (**q**) and quantification results showed that the protein level of GFAP (**r**) increased in the hippocampal tissues of cKO mice compared to Ctrl mice, but the level of Iba1 (**s**) showed no difference between Ctrl and cKO mice. n = 6 mice per genotype. Values represent mean ± SEM; *p < 0.05, **p < 0.01, ***p < 0.001; unpaired Student’s t-test

Next, we generated a *GlastCreERT2*::*Ogt*^loxp/Y^ (cKO) mouse model by crossing *Ogt*^loxp/loxp^ conditional allele with *GlastCreERT2* driver. Adult (postnatal 7-week) male mice were intraperitoneally (i.p.) administrated with multiple doses of tamoxifen (cKO) and corn oil (Ctrl) (1 time/day for 5 consecutive days, Additional file 1: Fig. S1h), respectively. Eight weeks after the final injection, astrocytes were isolated from the brains of Ctrl and cKO mice for in vitro assays, respectively. Both mRNA and protein levels of Ogt (Additional file 1: Fig. S1i–k), and the levels of O-GlcNAcylation and Oga (Additional file 1: Fig. S1j–m) were also significantly decreased in cKO cells compared to Ctrl cells. Immunostaining intensity of Ogt and O-GlcNAcylation signals were significantly decreased compared to those of Ctrl cells in vitro (Additional file 1: Fig. S1n, o). Interestingly, we noticed that the average area of cKO astrocytes (indicated by GFAP staining), the signal intensity and the protein level of GFAP were remarkably increased compared to Ctrl cells (Fig. 1a–d). mRNA levels of pan reactive astrocyte markers were also significantly increased in cKO astrocytes (Fig. 1e). Of note, mRNA levels of A1 type (neurotoxic) astrocyte markers were significantly increased; whereas, mRNA levels of A2 type (neuroprotective) astrocytic markers showed a subtle decrease (Fig. 1f, g). These results collectively suggest that *Ogt* deficiency leads to the activation of astrocytes in vitro*.*

Next, we aim to examine whether *Ogt* deficiency induces the activation of astrocytes in vivo. Immunostaining with brain sections revealed that Ogt and O-GlcNAcylation were significantly decreased in GFAP^+^ astrocytes of cKO mice (Additional file 1: Fig. S1p, q). Immunostaining intensity of GFAP and the number of astrocytes significantly increased in the hippocampal region of cKO mice compared to Ctrl mice (Fig. 1h–j). Three-dimension (3D) construction analysis showed that the volume, the number and process length of astrocytes significantly increased in the brain of cKO mice compared to Ctrl (Fig. 1k–o, Additional file 2: Fig. S2a), while the number and neurite length of microglia were not affected in the brain of mice (Additional file 2: Fig. S2b–g). Consistently, qRT-PCR and western blot results revealed a significant increase of GFAP but not Iba1 in the hippocampal tissue of cKO mice compared to Ctrl mice (Fig. 1p–s, Additional file 2: Fig. S2h).

Considering that few Glast^+^ astrocytes could be Nestin^+^ adult neural stem/progenitor cells (aNSPCs) in the brain of adult mice, we then examined whether *Ogt* deficiency in astrocytes affected neurogenesis. We observed that adult cKO mice showed the reduced number of BrdU^+^ cells and BrdU^+^DCX^+^ cells in the subgranular zone of hippocampus (Additional file 3: Fig. S3a–c) and subventricular zone (Additional file 3: Fig. S3e–g) compared to Ctrl, respectively, but the percentage of BrdU^+^DCX^+^/BrdU^+^ in these two regions showed no difference between Ctrl and cKO mice (Additional file 3: Fig. S3a, d, e, h). We further isolated aNSPCs from the forebrains of adult Ctrl and cKO mice, respectively. Ctrl and cKO NSPCs showed no difference in the levels of Ogt and O-GlcNAcylation (Additional file 3: Fig. S3i–k), and mRNA levels of proliferation and differentiation markers (Additional file 3: Fig. S3l–q). In addition, we did not observe the activation of astrocytes in the brain of *Nestin*CreERT2::*Ogt*^loxp/Y^ mice, in which *Ogt* is specific deficient in Nestin^+^ aNSPCs (Additional file 3: Fig. S3r, s). Collectively, these results suggest that *Ogt* deficiency in astrocytes specifically leads to astrocyte activation and impairs neurogenesis in vivo.

### Activated astrocytes induce inflammation in *Ogt* deficient mice

**Fig. 2:**
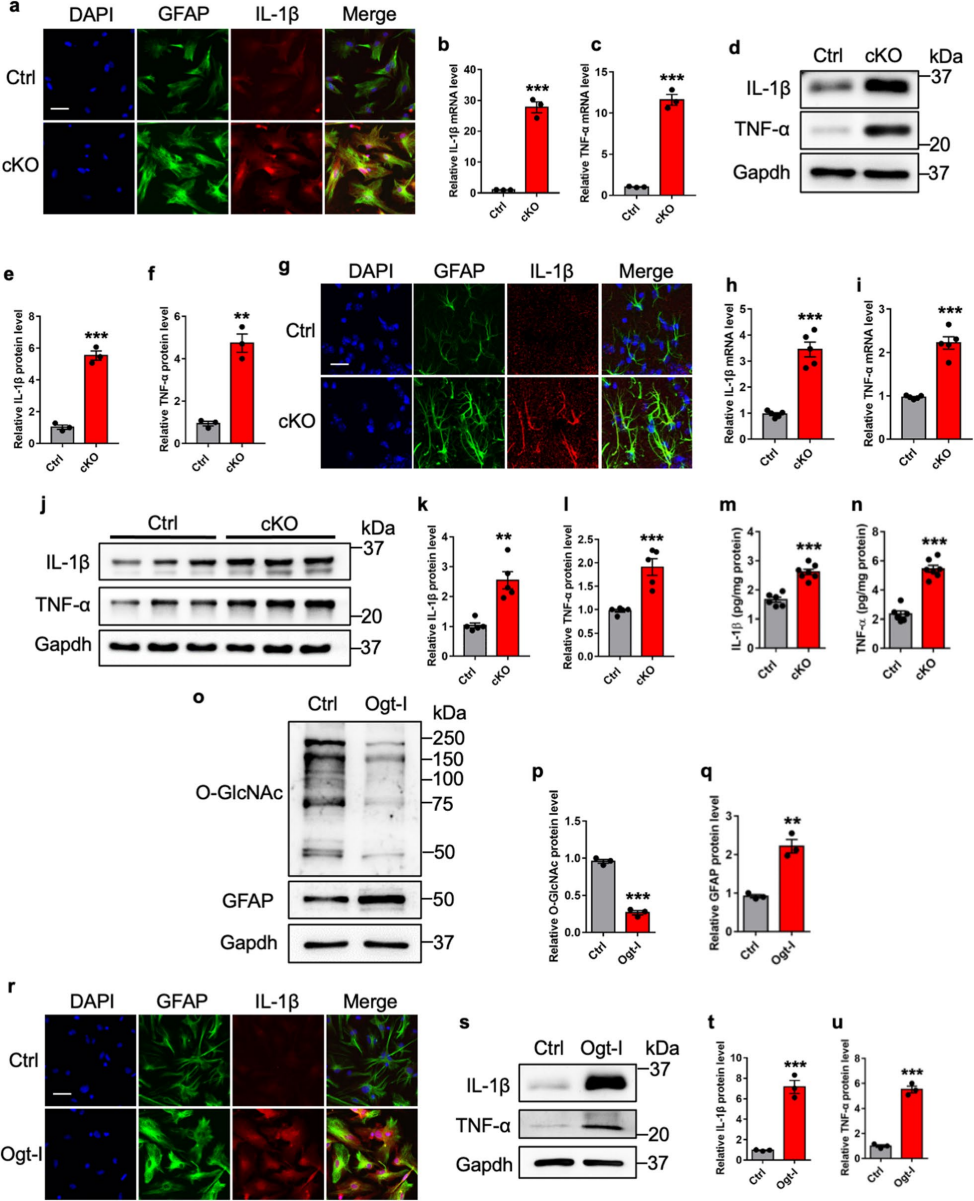
*Ogt* deficiency induces inflammatory response of astrocytes. **a** Representative images of GFAP and IL-1β immunostaining with cultured adult Ctrl and cKO astrocytes in vitro. Scale bar, 50 μm. **b**, **c** qRT-PCR results showed that mRNA levels of IL-1β (**b**) and TNF-α (**c**) increased in cKO astrocytes. n = 3 independent experiments. Values represent mean ± SEM; *p < 0.05, **p < 0.01, ***p < 0.001; unpaired Student’s t-test. **d**–**f** WB assay (**d**) and quantification results showed that protein levels of IL-1β (**e**) and TNF-α (**f**) significantly increased in adult cKO astrocytes in vitro. n = 3 independent experiments. Values represent mean ± SEM; *p < 0.05, **p < 0.01, ***p < 0.001; unpaired Student’s t-test. **g** Representative images of GFAP and IL-1β immunostaining in the hippocampus of brains of Ctrl and cKO mice. Scale bar, 20 μm. **h**, **i** qRT-PCR results showed that mRNA levels of IL-1β (**h**) and TNF-α (**i**) increased in the hippocampal tissues of cKO mice compared to Ctrl mice. n = 5 mice per genotype. Values represent mean ± SEM; *p < 0.05, **p < 0.01, ***p < 0.001; unpaired Student’s t-test. **j**–**l** WB assay (**j**) and quantification results showed that the IL-1β (**k**) and TNF-α (**l**) protein levels significantly increased in the hippocampal tissues of cKO mice compared to Ctrl mice. n = 5 mice per genotype. Values represent mean ± SEM; *p < 0.05, **p < 0.01, ***p < 0.001; unpaired Student’s t-test. **m**, **n** ELISA assay results show that the levels of IL-1β (**m**) and TNF-α (**n**) increased in the hippocampal tissue supernatants of cKO mice compared to Ctrl mice. n = 6 mice per genotype. Values represent mean ± SEM; *p < 0.05, **p < 0.01, ***p < 0.001; unpaired Student’s t-test. **o**–**q** WB assay (**o**) and quantification results showed that the treatment with Ogt inhibitor (Ogt-I) OSMI-4 reduced the level of O-GlcNAcylation in adult astrocytes (**p**), but increased the level of GFAP (**q**) compared to Ctrl group. n = 3 independent experiments. Values represent mean ± SEM; *p < 0.05, **p < 0.01, ***p < 0.001; unpaired Student’s t-test. **r** Representative images of GFAP and IL-1β immunostaining of adult astrocytes treated with PBS (Ctrl) and Ogt-I (20 μM for 72 h), respectively. Scale bar, 50 μm. **s**–**u** WB assay (**s**) and quantification results show that the treatment with Ogt inhibitor (Ogt-I) OSMI-4 significantly increased the levels of IL-1β (**t**) and TNF-α (**u**) of adult astrocytes in vitro. n = 3 independent experiments. Values represent mean ± SEM; *p < 0.05, **p < 0.01, ***p < 0.001; unpaired Student’s t-test

To determine whether activated astrocytes induced by *Ogt* deficiency drives inflammation, we first performed IL-1β immunostaining with cultured Ctrl and cKO astrocytes, respectively, and observed that the intensity of IL-1β signal increased in cKO astrocytes compared to Ctrl (Fig. 2a). qRT-PCR and WB results also showed that the levels of IL-1β and TNF-αwere significantly increased in cKO astrocytes (Fig. 2b–f).

Next, we examined the expression of IL-1β in the brains of Ctrl and cKO mice, and observed that the immunofluorescence intensity of IL-1β signal was remarkably increased (Fig. 2g). Consistently, qRT-PCR (Fig. 2h, i) and WB assays (Fig. 2j–l) results showed that the levels of IL-1β and TNF-α were significantly increased in the hippocampal tissues of cKO mice compared to Ctrl mice. ELISA results showed that the levels of IL-1β and TNF-α were significantly increased in the hippocampal tissue supernatants of cKO mice compared to Ctrl mice (Fig. 2m, n). Further, the exposure of an Ogt inhibitor, Ogt small molecule inhibitor-4 (OSMI-4), significantly reduced the level of O-GlcNAcylation in wild-type (WT) astrocytes (Fig. 2o, p), but significantly increased the of protein level of GFAP (Fig. 2o, q). In addition, the treatment with OSMI-4 also remarkably increased the staining intensity of GFAP and IL-1β (Fig. 2r), and the protein levels of IL-1β and TNF-α (Fig. 2s–u). These results collectively suggest that *Ogt* deficiency and inhibition of Ogt activity induce astrocytes activation and drive inflammation in vitro and in vivo.

### *Ogt* deficiency in astrocytes impairs neurons and cognitive function of mice

**Fig. 3:**
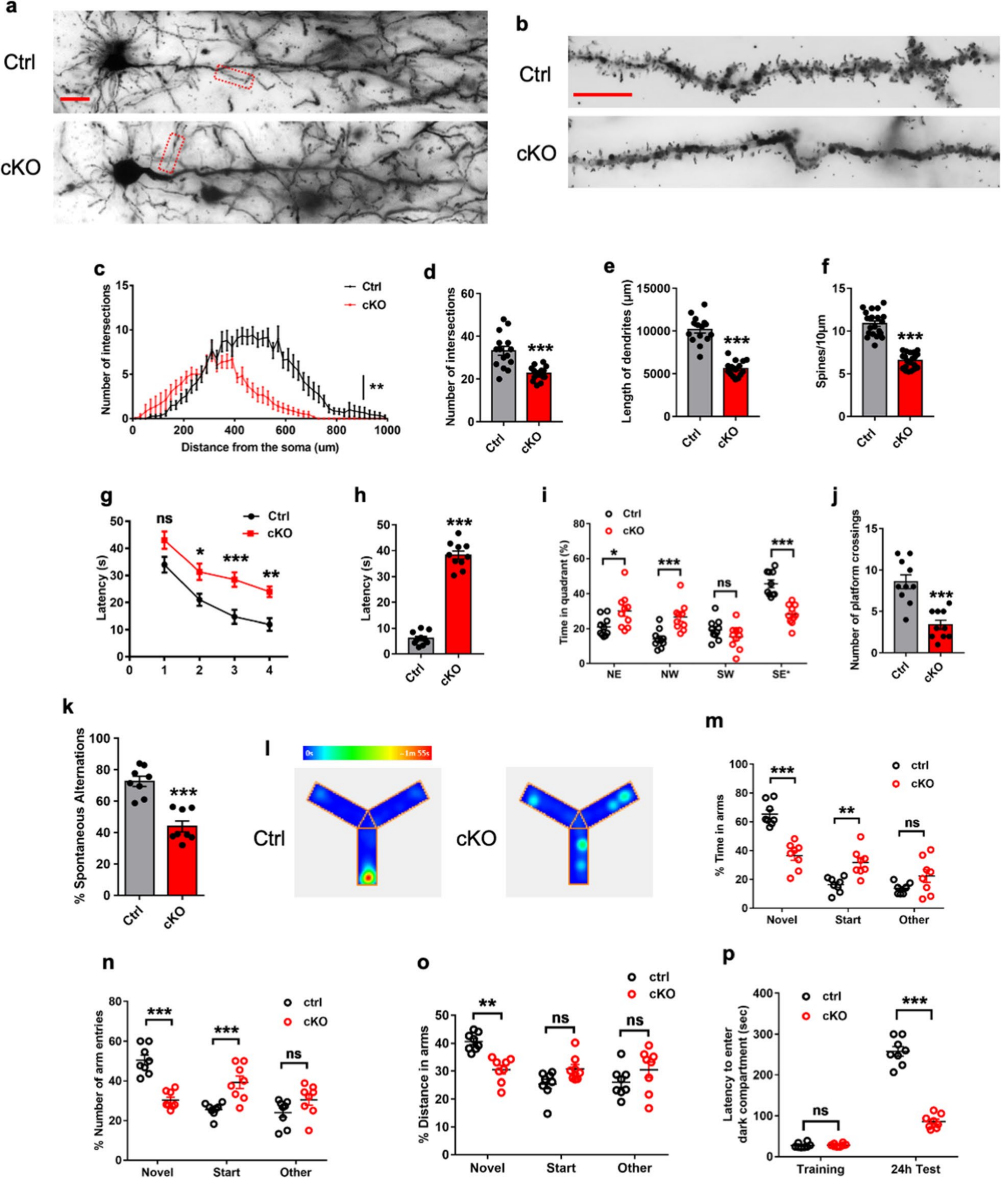
*Ogt* deficient astrocytes impair hippocampal neurons and mice cognition. **a** Representative images of neurons with Golgi staining in CA1 region of Ctrl and cKO mice. Scale bar, 10 μm. **b** Representative images of Golgi-stained dendritic spines of second-order segment in the CA1 region of Ctrl and cKO mice. Scale bar, 1 μm. **c**–**e** Sholl analysis showed the overall decrease in the number of dendritic intersections per radius (**c**) and the number of dendrites per cell (**d**) and total length of dendrites (**e**) in the Golgi-stained neurons from the CA1 region of cKO mice compared with Ctrl mice. n = 15 neurons from 3 mice per group were analyzed. Values represent mean ± SEM; *p < 0.05, **p < 0.01, ***p < 0.001; two-way ANOVA analysis followed by Sidak’s multiple-comparison test for **c**, *F*_(1, 1400)_ = 153.7; unpaired Student’s t-test for **d**, **e**. **f** Quantification results show the decreased dendritic spine density of cKO mice compared to that of Ctrl mice. Spine density was calculated by dividing the number of spines with the length of dendrite. n = 24 neurons from 3 mice per group were analyzed. Values represent mean ± SEM; *p < 0.05, **p < 0.01, ***p < 0.001; unpaired Student’s t-test. **g** The escape latency during the training period of Ctrl and cKO adult mice. cKO mice required longer time to reach the platform starting from the first day of the 4-day training period. Ctrl/cKO, n = 10 mice. Values represent mean ± SEM; *p < 0.05, **p < 0.01, ***p < 0.001; two-way ANOVA analysis followed by Sidak’s multiple-comparison test, *F*_(3, 72)_ = 23.06. **h**–**j** During the probe test, cKO mice required longer time to reach the platform (**h**), decreased time in target quadrant (**i**), and decreased numbers of crossing the platform (**j**). Ctrl mice, n = 10; cKO mice, n = 10. Values represent mean ± SEM; *p < 0.05, **p < 0.01, ***p < 0.001; unpaired Student’s t-test for **h**, **j** and two-way ANOVA analysis followed by Sidak’s multiple-comparison test for **i**, *F*_(3, 72)_ = 30.62. **k** The percentage of spontaneous alternations in cKO mice was lower than that in Ctrl mice during the Y maze spontaneous alternation task test. Ctrl, n = 8 mice; cKO, n = 8 mice. Values represent mean ± SEM; *p < 0.05, **p < 0.01, ***p < 0.001; unpaired Student’s t-test. **l** Representative heatmap shows the distribution of exploring time of Ctrl and cKO mice during the testing trial in Y maze spatial novelty preference test. **m**–**o** cKO mice showed the decreased percentage of exploring time (**m**), the number of entries (**n**) and distance (**o**) in the novel arm compared to those in Ctrl mice during the Y maze spatial novelty preference test. n = 8 mice for Ctrl/cKO group. Values represent mean ± SEM; *p < 0.05; **p < 0.01; ***p < 0.001; two-way ANOVA analysis followed by Sidak’s multiple-comparison test, *F*_(2, 42)_ = 67.41 for **m**, *F*_(2, 42)_ = 15.92 for **n**, *F*_(2, 42)_ = 10.84 for **o**. **p** In the passive avoidance task test, cKO mice displayed shorter latency to enter the dark compartment than that of Ctrl mice during the retention test while Ctrl and cKO mice showed no difference of the latency during the training session. n = 8 mice for Ctrl/cKO group. Values represent mean ± SEM; *p < 0.05; **p < 0.01; ***p < 0.001; two-way ANOVA analysis followed by Sidak’s multiple-comparison test, *F*_(1, 28)_ = 456.2

Next, we examined the effects of *Ogt* deficiency in astrocytes on neuronal cells. ELISA results showed that the levels of IL-1β and TNF-α significantly increased in the supernatants of cultured cKO astrocytes (Additional file 4: Fig. S4a, b). The supplementation of supernatants from cKO astrocytes significantly reduced the intersection number and dendritic length of cultured hippocampal neurons (Additional file 4: Fig. S4c–f). In addition, the treatment with the supernatants of cultured cKO astrocytes also significantly increased the number of active Caspase3 positive (aCaspase3^+^) cells (Additional file 4: Fig. S4g, h) and the protein level of aCaspase3 (Additional file 4: Fig. S4i, j). Golgi staining results showed that hippocampal neurons displayed shorter length of dendrites, decreased numbers of intersections and spines in the brain of *Ogt* deficient mice (Fig. 3a–f). Together, these results suggest that activated astrocytes induce inflammation and impair neurons in *Ogt* deficient mice.

Given the neurons were impaired in cKO mice, we next examined whether *Ogt* deficiency in astrocytes affects cognition of mice. Eight weeks after tamoxifen induction (Additional file 4: Fig. S4k), Morris water maze (MWM) test was performed and we observed that cKO mice displayed longer latency during the training period (Fig. 3g, Additional file 4: Fig. S4l). A probe test was performed 24 h after the training. Although Ctrl and cKO mice exhibited similar swimming speed and travelled paths (Additional file 4: Fig. S4m, n), cKO mice displayed the longer latency, the decreased time in the target quadrant and decreased numbers of crossing the platform (Fig. 3h–j). Y maze test results showed that cKO mice displayed the lower percentage of spontaneous alternations than Ctrl mice during the trial test (Fig. 3k), while Ctrl and *Ogt* cKO mice showed no difference between the times of arm entries and distance length (Additional file 4: Fig. S4o, p). For the spatial novelty preference test in the Y maze, cKO mice displayed a lower percentage of time exploring in the novel arm, decreased distance and numbers of arm entries compared to those of Ctrl mice (Fig. 3l–o). Further, cKO mice displayed shorter latency to enter the dark compartment than that of Ctrl mice during the retention test, while it showed no difference between groups during the training session in the passive avoidance task (Fig. 3p). Collectively, these results suggest that cKO mice have the impaired learning and memory.

### Restoration of O-GlcNAcylation inhibits astrocyte reactivation and inflammation

**Fig. 4:**
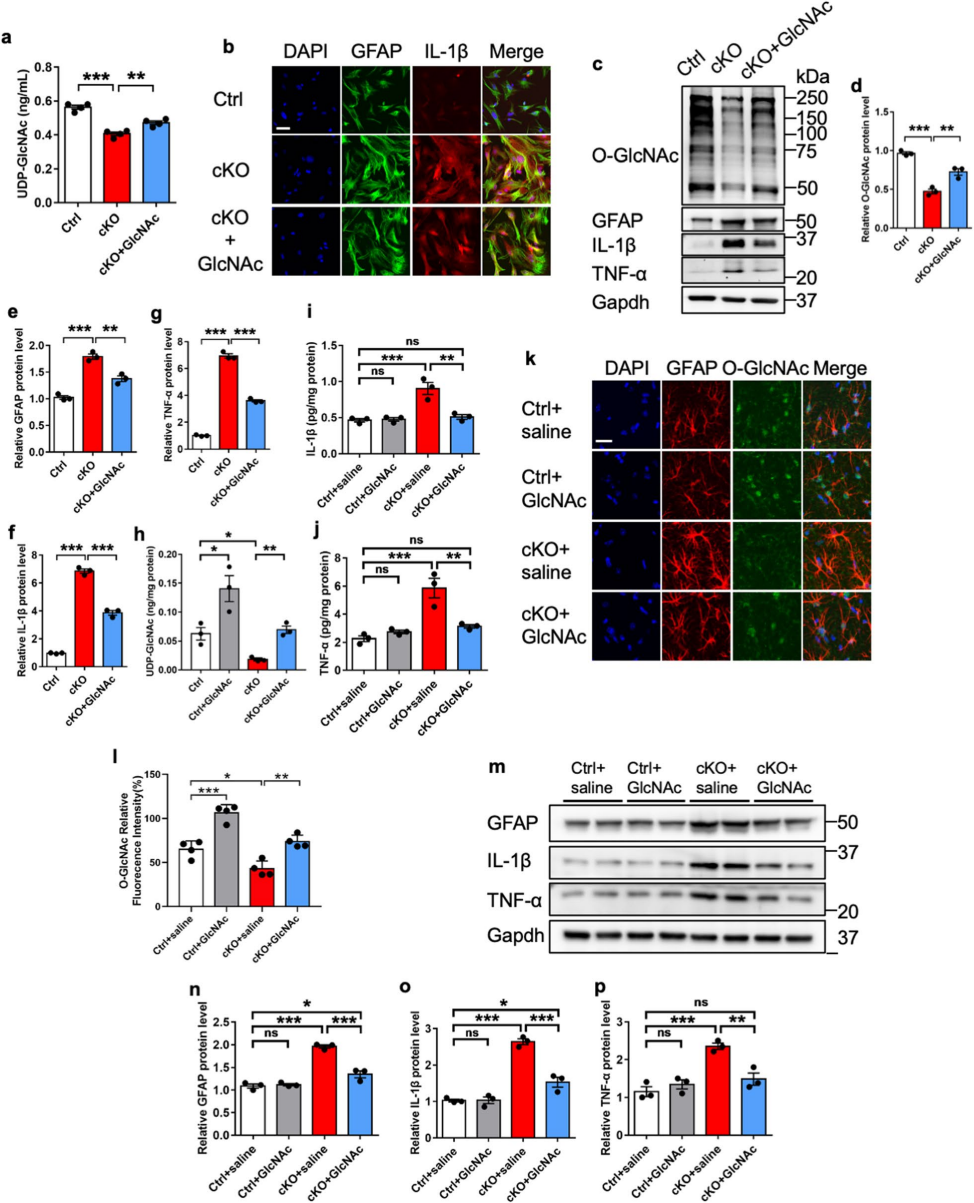
Restoration of O-GlcNAcylation inhibits reactivation and inflammation of cKO astrocytes in vitro and in vivo. **a** ELISA assay results showed that the level of UDP-GlcNAc significantly decreased in the supernatants of cKO astrocytes compared with Ctrl astrocytes, and the GlcNAc replenishment significantly increased UDP-GlcNAc level of cKO astrocytes. n = 4 independent experiments. Values represent mean ± SEM; *p < 0.05, **p < 0.01, ***p < 0.001; One-way ANOVA analysis followed by Tukey’s multiple-comparison test, *F*_(2, 9)_ = 53.04. **b** Representative images of GFAP and IL-1β immunostaining with adult Ctrl and cKO astrocytes treated with PBS (Ctrl and cKO) and O-GlcNAcylation substrate GlcNAc (20 μM, cKO + GlcNAc) for 72 h, respectively. Scale bar, 50 μm. **c**–**g** WB assay (**c**) and quantification results showed that GlcNAc supplementation significantly restored the decreased level of O-GlcNAcylation (**d**), and reduced the levels of GFAP (**e**), IL-1β (**f**) and TNF-α (**g**) of cKO astrocytes in vitro. n = 3 independent experiments. Values represent mean ± SEM; *p < 0.05, **p < 0.01, ***p < 0.001; one-way ANOVA analysis followed by Tukey’s multiple-comparison test, *F*_(2, 6)_ = 59.32 for **d**, *F*_(2, 6)_ = 69.6 for **e**, *F*_(2, 6)_ = 480.6 for **f**, *F*_(2, 6)_ = 788.7 for **g**. **h**–**j** ELISA result showed that GlcNAc supplementation significantly increased the level of UDP-GlcNAc (**h**), but reduced the levels of of IL-1β (**i**) and TNF-α (**j**) in the supernatants of adult cKO astrocytes. n = 3 independent experiments. Values represent mean ± SEM; *p < 0.05, **p < 0.01, ***p < 0.001; one-way ANOVA analysis followed by Tukey’s multiple-comparison test, *F*_(3, 8)_ = 15.49 for **h**, *F*_(3, 8)_ = 19.37 for **i**, *F*_(3, 8)_ = 19.08 for **j**. **k** Representative images of GFAP and O-GlcNAcylation immunostaining with the brain sections of adult Ctrl and cKO mice treated with saline (Ctrl + saline, cKO + saline) and GlcNAc (Ctrl + GlcNAc, cKO + GlcNAc), respectively. Scale bar, 50 μm. **l** Quantification results of O-GlcNAcylation immunostaining in **k** showed that GlcNAc supplementation significantly increased the immunostaining intensity of O-GlcNAcylation in the brain of cKO mice. n = 4 mice per group. Values represent mean ± SEM; *p < 0.05, **p < 0.01, ***p < 0.001; one-way ANOVA analysis followed by Tukey’s multiple-comparison test, *F*_(3, 12)_ = 36.08. **m**–**p** WB assay (**m**) and quantification results showed that GlcNAc supplementation significantly reduced the protein levels of GFAP (**n**), IL-1β (**o**) and TNF-α (**p**) in the hippocampal tissues of cKO mice compared with saline-treated cKO mice. n = 3 per group. Values represent mean ± SEM; *p < 0.05, **p < 0.01, ***p < 0.001; one-way ANOVA analysis followed by Tukey’s multiple-comparison test, *F*_(3, 8)_ = 65.8 for **n**, *F*_(3, 8)_ = 65.61 for **o**, *F*_(3, 8)_ = 17.5 for **p**

Previous studies have shown that *N*-acetylglucosamine (GlcNAc) could be converted to UDP-GlcNAc, the substrate of O-GlcNAcylation [3, 31, 57]. Next, we examined whether the restoration of O-GlcNAcylation via GlcNAc administration could inhibit astrocytes activation and inflammation in vitro. Ctrl and *Ogt*-deficient astrocytes were treated with phosphate buffer solution (PBS, Ctrl) and GlcNAc (20 μM) for 72 h, respectively. ELISA results showed that GlcNAc supplementation led to a significant increase of UDP-GlcNAc level (Fig. 4a). GlcNAc supplementation also reduced the staining intensity of GFAP and IL-1β of the cultured cKO astrocytes (Fig. 4b). WB assay and quantification results showed that GlcNAc supplementation significantly restored the level of O-GlcNAcylation (Fig. 4c, d) and remarkably reduced the levels of GFAP, IL-1β and TNF-α in cultured astrocytes (Fig. 4c, e–g).

Next, we examined the effects of the GlcNAc supplementation on astrocyte activation in vivo. Ctrl and cKO mice were administrated with saline and GlcNAc (400 mg/kg, i.p., 1 time/day for 14 days), respectively (Additional file 5: Fig. S5a). ELISA assay results showed that the level of UDP-GlcNAc was significantly increased in the supernatants of hippocampal tissue of cKO mice (Fig. 4h). ELISA assay results also showed that the levels of IL-1β and TNF-α were significantly decreased in the hippocampal tissue supernatants of GlcNAc-treated cKO mice compared to those of saline-treated cKO mice (Fig. 4i, j). Immunostaining showed that GlcNAc supplementation increased the intensity of O-GlcNAcylation in the brain of cKO mice (Fig. 4k, l). WB results showed that the GlcNAc supplementation significantly reduced the levels of GFAP, IL-1β and TNF-α in the hippocampal tissues of cKO mice (Fig. 4m–p). GlcNAc supplementation also remarkably reduced the immunostaining intensities of GFAP and IL-1β in the brain of cKO mice (Additional file 5: Fig. S5b). Taken together, these results suggest that the administration of GlcNAc restores O-GlcNAcylation, and inhibits astrocyte activation and inflammation in vitro and in vivo.

### Restoration of O-GlcNAcylation improves behavioral deficits of *Ogt* deficient mice

**Fig. 5:**
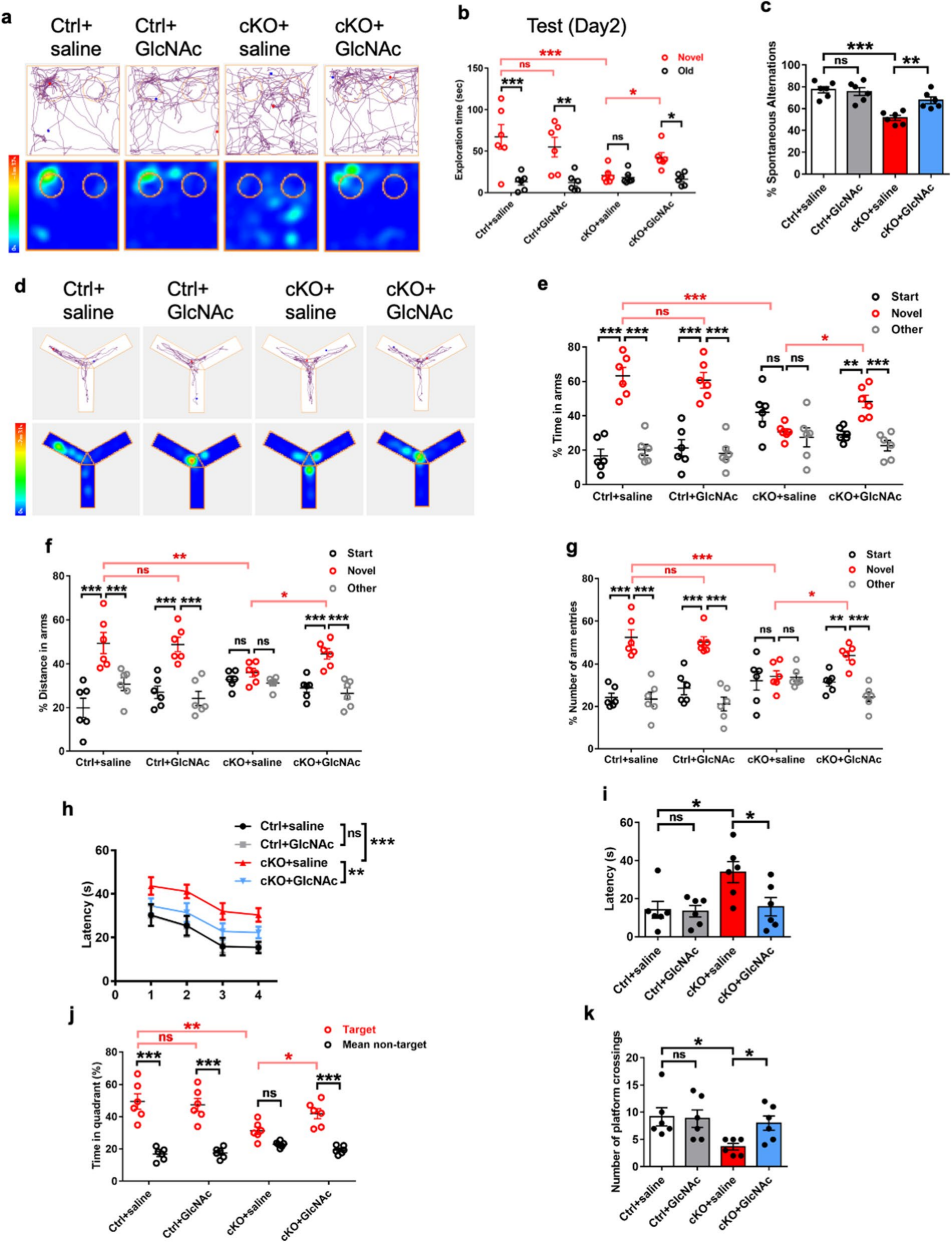
Restoration of O-GlcNAcylation improves behavioral deficits of *Ogt* deficient mice. **a** Representative images of the explored path (upper) and spent time (lower, heatmap) of novel object recognition task test of Ctrl and cKO mice treated with saline (Ctrl + saline, cKO + saline) and GlcNAc (Ctrl + GlcNAc, cKO + GlcNAc) during the testing trial, respectively. Heatmap image of each animal was shown in Additional file 5: Fig. S5e. **b** The time (seconds) spending in exploring the novel and old object of Ctrl and cKO adult mice treated with saline and GlcNAc during the testing trial. Compared to Ctrl mice, cKO mice spent less time exploring the novel object. cKO mice treated with GlcNAc displayed increased time exploring the novel object compared to cKO mice treated with saline during the testing trial. n = 6 mice per group. Values represent mean ± SEM; *p < 0.05, **p < 0.01, ***p < 0.001; two-way ANOVA analysis followed by Sidak’s multiple-comparison test, *F*_(3, 40)_ = 4.342. **c** Y maze spontaneous alternation task test results showed that cKO mice treated with GlcNAc displayed an increased percentage of spontaneous alternations compared to cKO mice treated with saline. n = 6 mice per group. Values represent mean ± SEM; *p < 0.05, **p < 0.01, ***p < 0.001; one-way ANOVA analysis followed by Tukey’s multiple-comparison test, *F*_(3, 20)_ = 14.94. **d** Representative travelled path and heatmap images showing the exploring path (upper) and spent time (lower, heat map) of Ctrl and cKO mice treated with saline and GlcNAc during Y maze spatial novelty preference test. Heatmap image for each animal was shown in supplemental Fig. 6c. **e**–**g** cKO mice treated with GlcNAc showed a higher percentage of exploring time (**e**), increased number of entries (**f**) and distance (**g**) in the novel arm compared to those of cKO mice treated with saline during Y maze spatial novelty preference test. n = 6 mice per group. Values represent mean ± SEM; *p < 0.05, **p < 0.01, ***p < 0.001; two-way ANOVA analysis followed by Sidak’s multiple-comparison test, *F*_(6, 60)_ = 11.13 for **e**, *F*_(6, 60)_ = 6.752 for **f**, *F*_(6, 60)_ = 4.623 for **g**. **h** The escape latency of Ctrl and cKO mice treated with saline and GlcNAc during the training period in Morris water maze test. cKO mice with the GlcNAc administration showed the decreased time to reach the platform of during 4-day training period compared to cKO mice treated with saline. n = 6 mice per group. Values represent mean ± SEM; *p < 0.05, **p < 0.01, ***p < 0.001; two-way ANOVA analysis followed by Sidak’s multiple-comparison test, *F*_(3, 80)_ = 14.31. **i**–**k** cKO mice treated with GlcNAc exhibited the reduced latency (**i**), increased time in target quadrant (**j**) and increased numbers of crossing the platform (**k**) compared to saline-treated cKO mice during the probe trial of Morris water maze test. n = 6 mice per group. Values represent mean ± SEM; *p < 0.05, **p < 0.01, ***p < 0.001; one-way ANOVA analysis followed by Tukey’s multiple-comparison test, *F*_(3, 20)_ = 4.668 for **i**; *F*_(3, 20)_ = 3.493 for **k**; two-way ANOVA analysis followed by Sidak’s multiple-comparison test for **j**, *F*_(3, 40)_ = 7.787

To examine whether the restoration of O-GlcNAcylation could improve the behavioral deficits of cKO mice, Ctrl and cKO mice were administrated with saline and GlcNAc for 14 days (400 mg/kg, i.p., 1 time/day), respectively, and a series of behavioral tests were performed (Additional file 5: Fig. S5c). A novel object recognition task test showed no difference in exploration time between groups during the training time (day 1) (Additional file 5: Fig. S5d), but cKO mice treated with GlcNAc displayed a longer time of exploring novel objects compared to the saline-treated cKO mice during the test (day 2) (Fig. 5a, b, Additional file 5: Fig. S5e). Y maze test results showed that GlcNAc-treated cKO mice showed a significant increase in the percentage of spontaneous alternation compared to saline-treated cKO mice (Fig. 5c). No difference was observed for the times of arm entries and the travelled distance between groups (Additional file 6: Fig. S6a, b). Y maze also showed that GlcNAc-treated cKO mice displayed the longer time, longer distance and higher numbers of entries to the novel arm compared to saline-treated cKO mice (Fig. 5d–g, Additional file 6: Fig. S6c).

Next, we performed Morris Water Maze test (MWM) and observed that cKO mice treated with the GlcNAc displayed shorter latency compared to saline-treated cKO mice during the training period (Fig. 5h). 24 h after training, the probe test results showed that cKO mice treated with GlcNAc exhibited the reduced latency, increased time in target quadrant and increased numbers of crossing the platform compared to saline-treated cKO mice (Fig. 5i–k). GlcNAc supplementation did not affect the swimming speed and travelled path of cKO mice (Additional file 6: Fig. S6d–f). Collectively, these results suggest that the restoration of GlcNAcylation improves the behavioral deficits of cKO mice.

### Ogt catalyzes the O-GlcNAcylation of NF-κB p65 and inhibits the activation of NF-κB signaling pathway

**Fig. 6:**
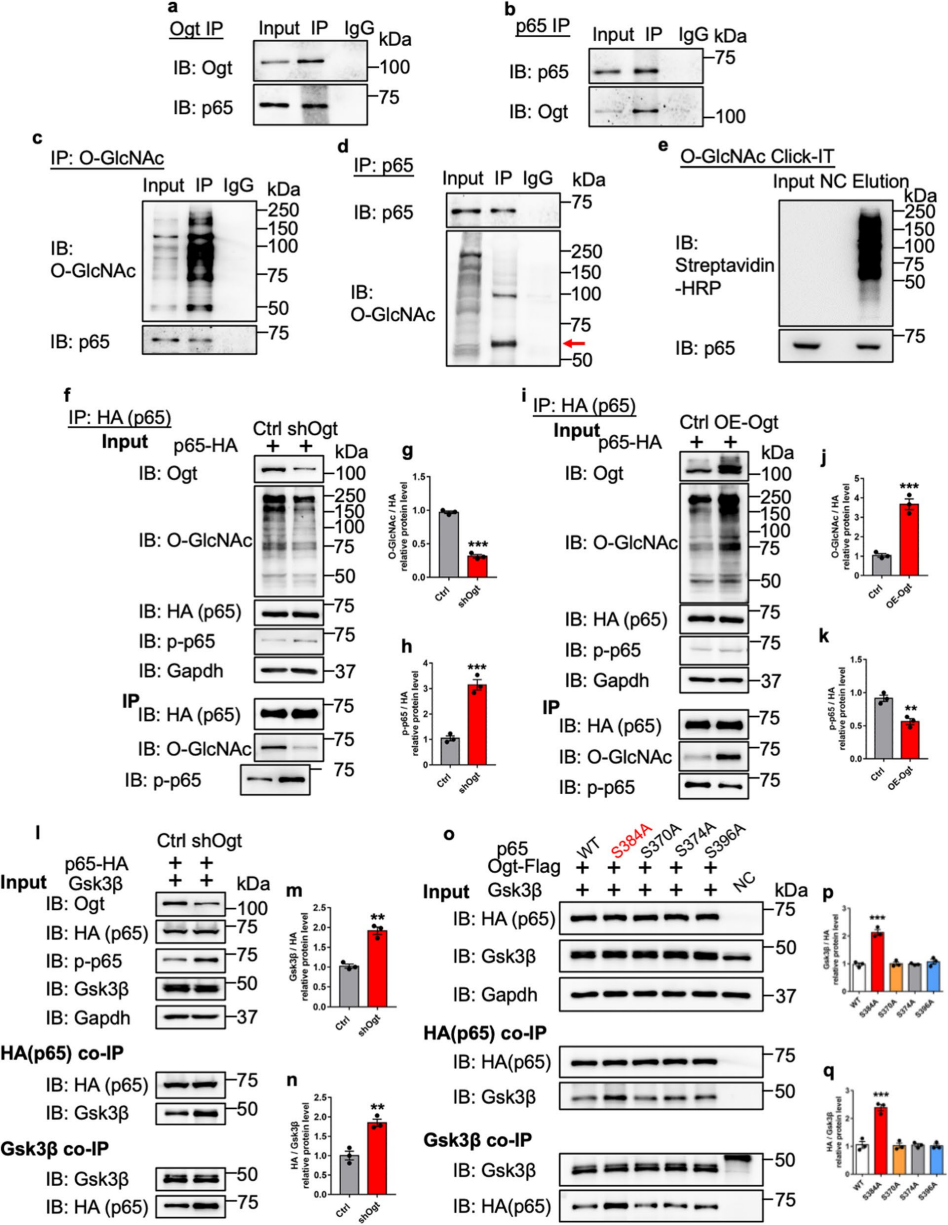
Ogt catalyzes O-GlcNAcylation of NF-κB on S384 and inhibits the activation of NF-κB signaling pathway. **a**, **b** Reciprocal IP-WB analysis showed that Ogt readily precipitated p65 (**a**), and vice versa in astrocytes (**b**). **c**, **d** Reciprocal IP-WB analysis showed that O-GlcNAcylation precipitated p65 (**c**), and vice versa in astrocytes (**d**). **e** Click-iT reaction results revealed that p65 was modified with O-GlcNAcylation. **f**–**h** WB assay showed that under *Ogt* knock down (KD) significantly decreased the levels of Ogt and O-GlcNAcylation, but significantly increased the level of p-p65 in N2a cells (**f**, Input part). IP with HA (HA-p65) antibody followed by WB assays (**f**, IP part) showed that O-GlcNAcylation levels of exogenous p65 (**g**) were significantly decreased, and the level of p-p65 (**h**) was significantly increased under *Ogt* KD condition. n = 3 independent experiments. Values represent mean ± SEM; *p < 0.05, **p < 0.01, ***p < 0.001; unpaired Student’s t-test. **i**–**k** WB assay showed that ectopic expression of Ogt (Ogt-Flag, OE-Ogt) and significantly increased the levels of Ogt and O-GlcNAcylation without affecting the levels of total p65 and p-p65 in N2a cells (**i**, Input part). IP with HA (HA-p65) antibody followed by WB assays (**i**) showed that the O-GlcNAcylation level of p65 (p65-HA) (**j**, IP) significantly increased, but the level of p-p65 (**k**) significantly decreased. n = 3 independent experiments. Values represent mean ± SEM; *p < 0.05, **p < 0.01, ***p < 0.001; unpaired Student’s t-test. **l**–**n** IP followed by WB assays showed that *Ogt* KD promoted the binding of Gsk3β to p-65. n = 3 independent experiments. Values represent mean ± SEM; *p < 0.05, **p < 0.01, ***p < 0.001; unpaired Student’s t-test. **o**–**q** IP followed by WB assays showed that mutation of S384A significantly increased the binding of Gsk3β to p-65 compared to other O-GlcNAcylation sites. n = 3 independent experiments. Values represent mean ± SEM; *p < 0.05, **p < 0.01, ***p < 0.001; one-way ANOVA analysis followed by Tukey’s multiple-comparison test, *F*_(4, 10)_ = 47.49 for **p**, *F*_(4, 10)_ = 42.31 for **q**

Previous studies have shown the important function of the NF-κB (p65) signaling pathway in inflammation [5, 11, 30, 35, 38, 59, 79]. Next, we examined whether *Ogt* deficiency induced inflammation through regulating NF-κB signaling pathway. co-immunoprecipitation (co-IP) showed that Ogt directly interacts with NF-κB p65, and vice versa, in cultured astrocytes (Fig. 6a, b). A pan-O-GlcNAcylation antibody could also precipitate p65, and vice versa, in cultured astrocytes (Fig. 6c, d). Chemoenzymatic labeling assay [32, 43] results showed that p65 was modified with O-GlcNAcylation (Fig. 6e). Under *Ogt* knock down (KD) condition, IP followed by WB assays showed that *Ogt* KD significantly decreased the O-GlcNAcylation of p65 (p65-HA), but significantly increased the level of p-p65, an active form of p65, in N2a cells (Fig. 6f–h). Consistently, IP followed by WB assays showed that ectopic Ogt significantly increased the O-GlcNAcylation of p65, but decreased the level of p-p65 in N2a cells (Fig. 6i–k).

To uncover the O-GlcNAcylation sites of p65, ectopic expression of p65 were performed followed by O-GlcNAcylation immunoprecipitation (IP) in N2a cells, and the precipitates were subjected to liquid chromatography coupled tandem mass spectrometry (LC–MS/MS). Four potential O-GlcNAcylation sites, Ser370, Ser374, Ser384, and Ser396 were identified on p65 (Additional file 7: Fig. S7a). Among these sites, the O-GlcNAcylation of Ser370, Ser384, and Ser396 had not been reported previously. The mutation of each single site (S370A, S374A, S384A, or S396A) led to a significant decrease of the O-GlcNAcylation level of p65, with the mutation of S384A and S396A showing the most dramatic effect (Additional file 7: Fig. S7b, c). Of note, only the mutation of S384A induced a significant increase of p-p65 level (Additional file 7: Fig. S7b, d).

To identify the proteins which phosphorylated p65, hippocampal tissue from Ctrl and cKO mice were performed IP with p65 antibody and the precipitates were subjected to liquid chromatography coupled tandem mass spectrometry (LC–MS/MS). MS/MS data analysis revealed 392 and 458 proteins in the precipitates of Ctrl and cKO mice, respectively (Additional file 11: Table S1). Of note, glycogen synthase kinase 3β (GSK3β) was specifically identified in cKO group. GSK3β has kinase activity and phosphorylates NF-κB p65 [65]. Co-IP with hippocampal tissues of Ctrl and cKO mice showed that the interaction between p65-GSK3β significantly increased in AD mice compared to Ctrl (Additional file 7: Fig. S7e–i). IP followed by WB assays showed that p65 directly interacted with glycogen synthase kinase 3β (Gsk3β), and *Ogt* KD promoted the binding of Gsk3β to p65 (Fig. 6l–n). Further, S384A mutation of Ogt significantly increased the binding of Gsk3β to p65 compared to the mutations of other O-GlcNAcylation sites (Fig. 6o–q). Of note, we did not observe a direct interaction between Gsk3β and Ogt (Additional file 7: Fig. S7j).

Next, we performed RNA-seq with Ctrl and *Ogt* cKO astrocytes. RNA-seq data analysis identified that 4269 genes showed differential expression, with 2343 genes up-regulated and 1926 genes down-regulated (Additional file 8: Fig. S8a, Additional file 12: Table S2). Gene ontology (GO) analysis revealed that up-regulated genes enriched terms for inflammatory response, cytokine signaling and NF-κB signaling pathway; whereas, down-regulated genes enriched for axon development and learning and memory (Additional file 8: Fig. S8b). WB results showed that the level of total p65 was not altered, but the level of p-p65 was significantly increased in cKO astrocytes compared to that of Ctrl cells (Additional file 8: Fig. S8c–e). In addition, we observed that cytoplasmic p65 was significantly decreased (Additional file 8: Fig. S8f, g) and nucleic p-p65 significantly increased at protein levels (Additional file 8: Fig. S8h, i). These results collectively suggest that Ogt regulates NF-κB signaling pathway through catalyzing O-GlcNAcylation on S384 of NF-κB p65.

### *Ogt* depletion induces inflammation by activating NF-κB signaling in human astrocytes

**Fig. 7:**
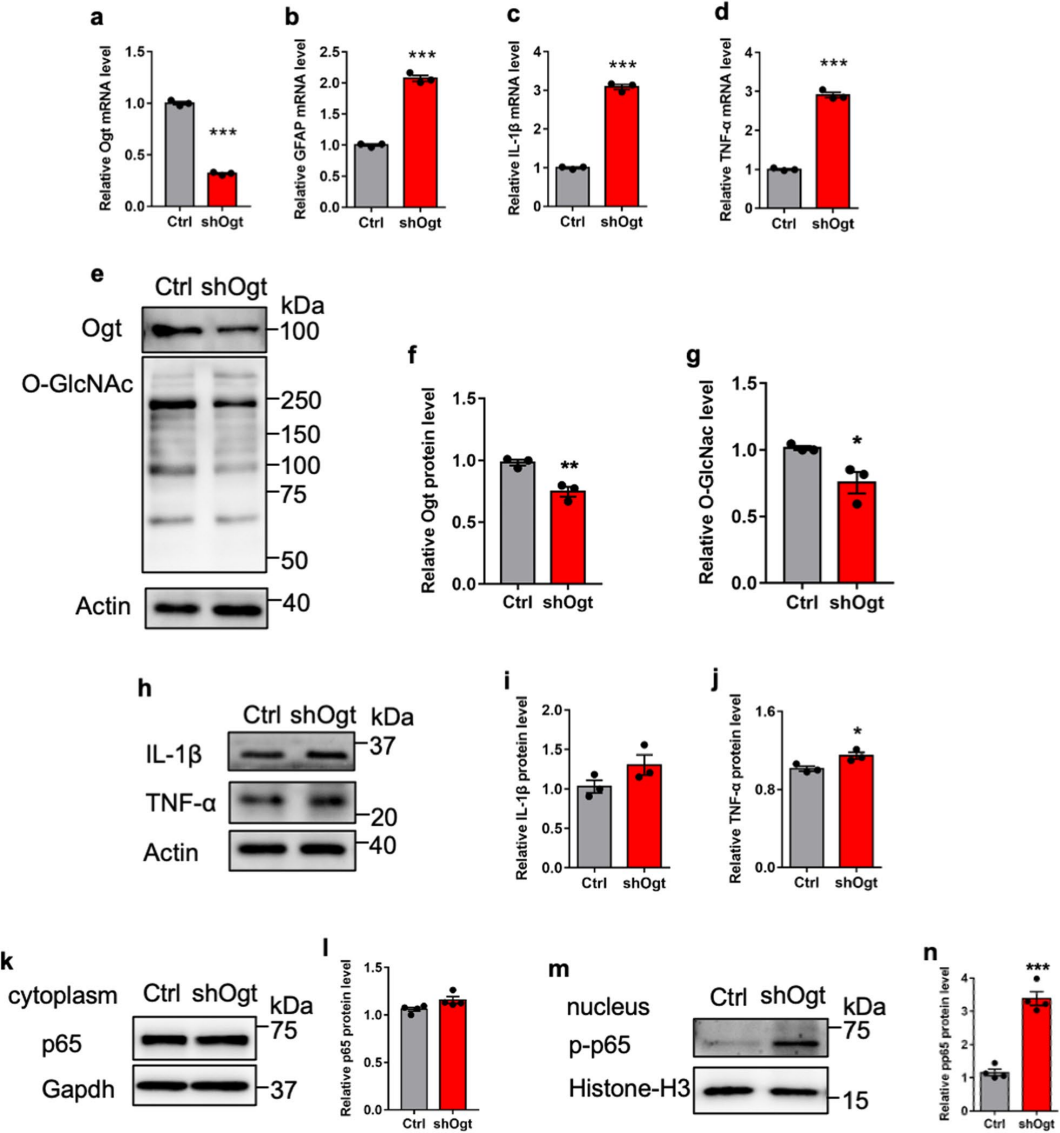
*Ogt* depletion reduces the level of O-GlcNAcylation and induces activation of astrocytes derived from iPSCs. **a** qRT-PCR results showed that *Ogt* depletion significantly reduced the level of *Ogt* in astrocytes derived from iPSCs. n = 3 independent experiments. Values represent mean ± SEM; *p < 0.05, **p < 0.01, ***p < 0.001; unpaired Student’s t-test. **b**–**d** qRT-PCR results showed that *Ogt* depletion significantly enhanced the level of *Gfap* (**b**), *IL-1β* (**c**) and *TNF-α* (**d**) in astrocytes derived from iPSCs. n = 3 independent experiments. Values represent mean ± SEM; *p < 0.05, **p < 0.01, ***p < 0.001; unpaired Student’s t-test. **e**–**g** WB assay (**e**) and quantification results showed that *Ogt* depletion significantly enhanced the level of Ogt (**f**) and O-GlcNAcylation (**g**) in astrocytes derived from iPSCs. n = 3 independent experiments. Values represent mean ± SEM; *p < 0.05, **p < 0.01, ***p < 0.001; unpaired Student’s t-test. **h**–**j** WB assay (**h**) and quantification results showed that *Ogt* depletion significantly enhanced the levels of of IL-1β (**i**) and TNF-α (**j**) in astrocytes derived from iPSCs. n = 3 independent experiments. Values represent mean ± SEM; *p < 0.05, **p < 0.01, ***p < 0.001; unpaired Student’s t-test. **k**–**n** WB assay and quantification results showed that *Ogt* depletion did not affect the level of cytoplasmic p65 in astrocytes derived from iPSCs (**k**, **l**), but significantly increased the level of nuclear p-p65 (**m**, **n**). n = 4 independent experiments. Values represent mean ± SEM; *p < 0.05, **p < 0.01, ***p < 0.001; unpaired Student’s t-test

Next, we aim to examine whether *Ogt* deficiency also induces inflammation in human astrocytes. Induced pluripotent stem cells (iPSCs) were generated by reprogramming urinary cells with Sendai virus following the manufacturer’s protocol, and were induced differentiation towards astrocyte lineage. Astrocytes were infected with lentivirus expressing shRNA against human *Ogt*. We observed that *Ogt* KD significantly reduced *Ogt* level, and remarkably increased mRNA levels of *GFAP*, *IL-1β* and *TNF-α* in human astrocytes (Fig. 7a–d). *Ogt* KD significantly reduced the levels of Ogt protein and O-GlcNAcylation (Fig. 7e–g), but remarkably increased the protein level of TNF-α in human astrocytes, while the level of IL-1β showed no difference (Fig. 7h–j). In addition, WB results showed that *Ogt* KD significantly increased the level of nuclear p-p65 in astrocytes, but did not affect the level of cytoplasmic p-65 (Fig. 7k–n). These results suggest that *Ogt* deficiency induces human astrocytes activation and inflammation.

### Restoration of O-GlcNAcylation inhibits inflammatory activation of AD astrocytes

**Fig. 8:**
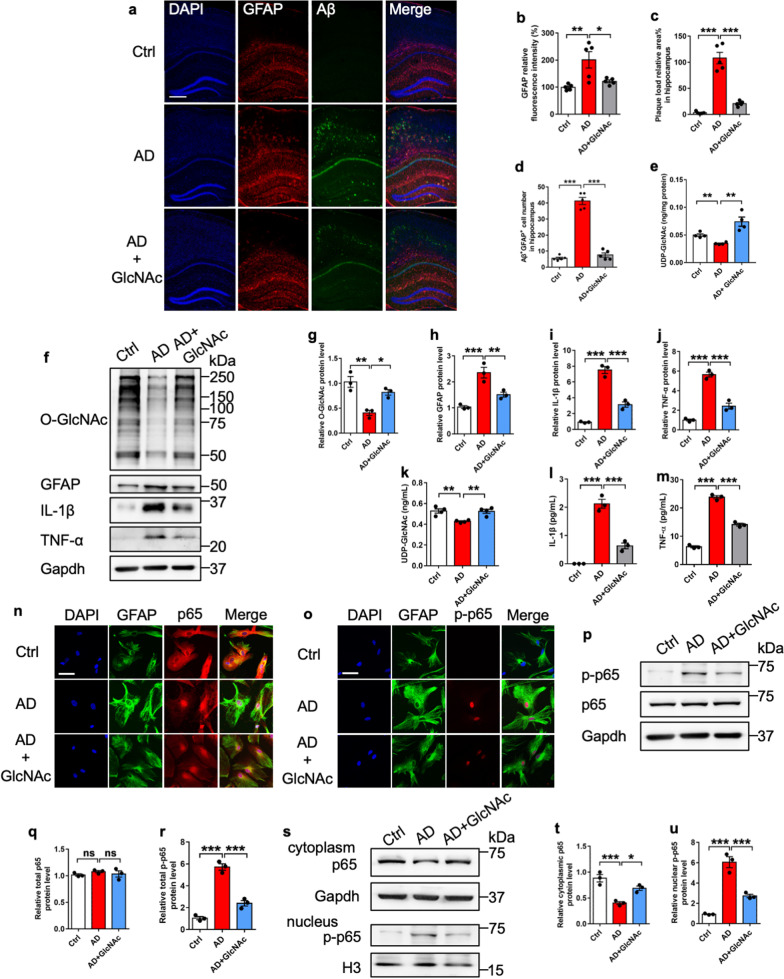
Restoration of O-GlcNAcylation inhibits astrocytes reactivation of AD model mice. **a**–**d** Immunostaining (**a**) and quantification results showed that GlcNAc administration significantly reduced GFAP immunostaining intensity (**b**), Aβ plaque area (**c**) and the number of GFAP^+^Aβ^+^ cells (**d**) in the hippocampus region of AD mice compared to Ctrl mice, respectively. 4–5 sections were picked up per animal and n = 5 mice per group. Values represent mean ± SEM; *p < 0.05, **p < 0.01, ***p < 0.001; one-way ANOVA analysis followed by Tukey’s multiple-comparison test, *F*_(2, 12)_ = 8.772 for **b**, *F*_(2, 12)_ = 75.82 for **c** and *F*_(2, 12)_ = 172.7 for **d**. **e** ELISA assay results show that the GlcNAc supplementation increased the levels of UDP-GlcNAc in the supernatants of hippocampal tissues of GlcNAc-treated AD mice compared with saline-treated AD mice. n = 4 mice per group. Values represent mean ± SEM; *p < 0.05, **p < 0.01, ***p < 0.001; one-way ANOVA analysis followed by Tukey’s multiple-comparison test, *F*_(2, 9)_ = 16.13. **f**–**j** WB assay (**f**) and quantification results showed that GlcNAc supplementation significantly restored the level of O-GlcNAcylation (**g**), and decreased the levels of GFAP (**h**), IL-1β (**i**) and TNF-α (**j**) of AD astrocytes compared to PBS-treated AD cells. n = 3 independent experiments. Values represent mean ± SEM; *p < 0.05, **p < 0.01, ***p < 0.001; one-way ANOVA analysis followed by Tukey’s multiple-comparison test, *F*_(2, 6)_ = 17.08 for (**g**), *F*_(2, 6)_ = 24.8 for **h**, *F*_(2, 6)_ = 108.5 for **i**, *F*_(2, 6)_ = 102.3 for **j**. **k**–**m** ELISA assay results showed that the GlcNAc supplementation increased the level of UDP-GlcNAc (**k**), and reduced the levels of IL-1β (**l**) and TNF-α (**m**) in the supernatants of cultured AD astrocytes compared with PBS-treated AD astrocytes. n = 4 independent experiments for **k** and n = 3 independent experiments for **l**, **m**. Values represent mean ± SEM; *p < 0.05, **p < 0.01, ***p < 0.001; one-way ANOVA analysis followed by Tukey’s multiple-comparison test, *F*_(2, 9)_ = 12.46 for **k**, *F*_(2, 6)_ = 100.2 for **l**, *F*_(2, 6)_ = 327.7 for **m**. **n** Representative images of GFAP and p65 immunostaining with Ctrl and AD adult astrocytes treated with PBS (Ctrl and AD) and GlcNAc (20 μM, AD + GlcNAc) for 72 h, respectively. Scale bar, 50 μm. **o** Representative images of GFAP and p-p65 immunostaining with Ctrl and AD adult astrocytes treated with PBS (Ctrl and AD) and GlcNAc (20 μM, AD + GlcNAc) for 72 h, respectively. Scale bar, 50 μm. **p**–**r** WB assay (**p**) and quantification results showed that the GlcNAc supplementation did not affect the level of total p65 (**q**), but significantly increased the protein level of p-p65 (**r**) in AD astrocytes. n = 3 independent experiments. Values represent mean ± SEM; *p < 0.05, **p < 0.01, ***p < 0.001; one-way ANOVA analysis followed by Tukey’s multiple-comparison test, *F*_(2, 6)_ = 0.6736 for **q**, *F*_(2, 6)_ = 100.1 for **r**. **s**–**u** WB assay (**s**) and quantification results showed that GlcNAc supplementation significantly restored the level of cytoplasmic p65 (**t**) and reduced the level of p-p65 in nucleus (**u**). n = 3 independent experiments. Values represent mean ± SEM; *p < 0.05, **p < 0.01, ***p < 0.001; one-way ANOVA analysis followed by Tukey’s multiple-comparison test, *F*_(2, 6)_ = 23.04 for **t**, *F*_(2, 6)_ = 63.16 for **u**

Finally, we aim to examine the roles of O-GlcNAcylation in Alzheimer’s disease (AD) mouse model. Previous studies have shown the decreased O-GlcNAcylation and the activation of astrocytes in the brain of the AD mouse model and AD patients [52, 53, 56, 69, 73, 76, 77]. Ctrl and AD mice (postnatal 10-week) were administrated with GlcNAc (600 mg/kg, i.p., 1 time/day for 6 weeks) (Additional file 9: Fig. S9a). Immunostaining and quantification results showed the significant increase of GFAP and Aβ intensities in AD mice, which can be remarkably reduced by GlcNAc replenishment (Fig. 8a–c). GlcNAc supplementation also significantly reduced the number of GFAP^+^Aβ^+^ cells (Fig. 8d). ELISA results showed that the level of UDP-GlcNAc significantly increased in the brains of GlcNAc-treated AD mice (Fig. 8e). the number of GlcNAc administration significantly increased the intensity of O-GlcNAcylation, but reduced the intensity of Iba1 in the brain of AD mice (Additional file 9: Fig. S9b–d).

Next, we isolated astrocytes from the adult Ctrl and AD mice (postnatal 6-month). Compared to Ctrl cells, AD astrocytes displayed the decreased level of O-GlcNAcylation, Ogt and Oga, but the increased levels of APP (Additional file 9: Fig. S9f–j). WB assays and quantification results showed that GlcNAc administration significantly restored the level of O-GlcNAcylation (Fig. 8f, g), but also significantly reduced the levels of GFAP, IL-1β and TNF-α in AD astrocytes (Fig. 8f, h–j). ELISA results also showed that the level of UDP-GlcNAc significantly increased, but significantly reduced the levels of IL-1β and TNF-α upon GlcNAc supplementation in the astrocytes of AD mice (Fig. 8k–m).

Next, we examined the interaction between Ogt, p65 and Gsk3β in the brain of AD mice. WB assay and quantification results showed the decreased levels of O-GlcNAcylation and Ogt, while the levels of total p65 and Gsk3β showed no difference in the brain of AD mice (Additional file 9: Fig. S9j–n, input part). IP followed by WB assays showed that the O-GlcNAcylation on p65, and the interaction between p65 and Ogt was significantly decreased in AD mice, but the interaction between p65 and Gsk3β was significantly increased in the hippocampus of AD mice compared to Ctrl mice (Additional file 9: Fig. S9j, IP part, Additional file 9: Fig. S9o–t).

Next, we examined the effects of GlcNAc supplementation with Ctrl and AD astrocytes. Immunostaining showed that the GlcNAc supplementation reduced the signal intensities of GFAP, p65 and nucleic p-p65 of AD astrocytes (Fig. 8n, o). WB results showed that the GlcNAc supplementation significantly reduced the levels of p-p65 in AD astrocytes, but it did not affect the level of total p65 (Fig. 8p–r). Furthermore, GlcNAc supplementation led to a significant increase of cytoplasmic p65 and a significant decrease of nucleic p-p65 (Fig. 8s–u). Collectively, these results suggest that the restoration of O-GlcNAcylation inhibited activation of AD astrocytes by repressing NF-κB signaling pathway.

## Discussion

In this study we showed that *Ogt* deficiency leads to activation of astrocytes and subsequently induces inflammation in vitro and in vivo. Activated astrocytes impair neurons and cognitive function of mice. Mechanistically, *Ogt* directly interacts with NF-κB p65 and catalyzes the O-GlcNAc modification of NF-κB p65. *Ogt* deficiency induces the activation of NF-κB signaling by promoting the binding of Gsk3β to NF-κB. Further, restoration of O-GlcNAcylation inhibits astrocytes activation and inflammation, and improves cognitive function of mice. The depletion of *Ogt* in human astrocytes derived from the induced pluripotent stem cells (iPSCs) also induces inflammation and activates NF-κB signaling. The activation of astrocytes, inflammation and activated NF-κB signaling in Alzheimer’s disease (AD) model mice can be significantly inhibited by restoration of O-GlcNAcylation can inhibit the activation of AD astrocytes. Our findings indicate the essential roles of Ogt-mediated O-GlcNAcylation in regulating astrocytes function under normal and neurodegenerative conditions.

As a post-translational modification, O-GlcNAcylation modifies hundreds of proteins, displaying dynamic and reversible features, and regulates fundamental biological processes [75]. Previous studies have shown that O-GlcNAcylation plays important functions in regulating neuronal development, cell survival and neurogenesis [7, 24, 33, 36, 40, 62, 67, 72, 75]. Our present study showed that specific deletion of *Ogt* in astrocytes reduces O-GlcNAcylation of astrocytes, but not affects O-GlcNAcylation in other cells such as adult neural/progenitor cells (aNSPCs). The decreased O-GlcNAcylation of astrocytes induces activation and inflammation in vitro and in vivo. Activated astrocytes not only impair neuronal function, also impair neurogenesis in vivo, but not in vitro, suggesting that it was potentially caused by inflammatory signals from activated astrocytes in vivo, but not due to the lineage leaking.

In the present study, we adopted a tamoxifen induction strategy to delete *Ogt* in astrocytes. Based on our results of immunostaining, qRT-PCR and western blot assays, the level of Ogt is significantly reduced, though there are some residues, and the level of O-GlcNAcylation is also remarkably decreased. Consistently, the level of O-GlcNAcylation can be restored by the administration of GlcNAc in *Ogt* deficient mice. The content of UDP-GlcNAc, the substrate of O-GlcNAcylation, is also increased in the brain of *Ogt* cKO mice by GlcNAc administration, which are consistent with previous studies that showing GlcNAc can be converted to UDP-GlcNAc [3, 31]. Our results also show that the level of O-GlcNAcase (Oga) decreases in *Ogt* deficient astrocytes. Previous study indicated that Oga is sensitive to the alteration of O-GlcNAc homeostasis [80]. We speculate that Oga could play important roles when the O-GlcNAcylation system is disrupted.

Reactive astrocytes involve in CNS inflammation [44–46, 71], and have also been detected in neurodegenerative diseases including multiple sclerosis (MS) and Alzheimer’s disease (AD) [22, 23, 46, 51, 60, 71]. Diverse factors, including epigenetic modifications, environmental factors, metabolism, bacterial challenges, modulate astrocyte activation [22, 46, 51, 60, 71]. Our present study has shown that reduced O-GlcNAcylation induces the activation of mouse astrocytes, which are mainly type A1, i.e., neurotoxic astrocytes, and drives neuroinflammation. A recent study has shown that under depression condition, astrocytic Ogt in the medial prefrontal cortex (mPFC) is up-regulated and specific deletion of *Ogt* in astrocytes produces anti-depression-like effects through regulating glutamate transporter-1 (GLT-1) [19]. Collectively, these results highlight the important roles of O-GlcNAcylation in astrocytes.

The activation of astrocytes is regulated by multiple pathways including the Janus kinase/signal transducer and activator of transcription 3 (JAK/STAT3) pathway, the Sonic hedgehog (SHH) pathway, the mitogen-activated protein kinase (MAPK) pathway and the nuclear factor kappa-light-chain-enhancer of activated B cells (NF-κB) pathway [22, 28, 41, 68]. NF-κB family of transcription factors consists of multiple members and plays pivotal roles in inflammation [13, 14, 18, 46, 74], which is regulated by many factors including post-translational modifications such as O-GlcNAcylation and phosphorylation [13, 17, 47, 50, 82]. Multiple sites of O-GlcNAcylation have been identified on NF-κB, and the interaction between O-GlcNAcylation and phosphorylation is important for NF-κB signaling in immune cells [2, 13, 17, 49, 74]. Our present study reveals a novel O-GlcNAcylation site of NF-κB, Ser-384, which is important for the activation of NF-κB in astrocytes. The mutagenesis of Ser-384 significantly decreased the level of O-GlcNAcylation of NF-κB, and subsequently induces its activation by promoting the binding of Gsk3β. In addition, our results show that *Ogt* deficiency also induces p65 phosphorylation, astrocyte activation and inflammation in human astrocytes. Therefore, our study provides a novel insight into the regulation of O-GlcNAcylation on astrocyte-inducing inflammation, which is conserved between humans and mice.

The level of O-GlcNAcylation decreases with ageing of normal and AD brains, which was associated with neurodegeneration [67, 69, 70, 76]. The decreased O-GlcNAcylation could be caused by the decreased activity of Ogt, the increased activity of Oga and the decreased content of UDP-GlcNAc, the substrate of O-GlcNAcylation. We observe that the levels of O-GlcNAcylation (around 60%), Ogt (around 30%), Oga (around 50%), and UDP-GlcNAc (around 20%) decreases in AD astrocytes compared to Ctrl astrocytes, respectively, suggesting a dysregulation of O-GlcNAcylation process, which is consistent with previous study [15]. The restoration of O-GlcNAcylation inhibits astrocytes activation and inflammation, and also improves the learning and memory of *Ogt* deficient mice. We hold the opinion that Ogt plays a critical role in regulating the O-GlcNAcylation homeostasis in AD astrocytes.

Decreased O-GlcNAcylation is involved in ageing and neurodegeneration [48, 53, 67, 69, 70]. The restoration of O-GlcNAcylation level in neuronal cells improves cognition of mice [70].The inhibition of *Oga* increases GlcNAcylation of AD mice, and reduces AD pathology including the impaired cognition and Aβ plaque [52]. Our present results show that the remarkable astrocyte activation and Aβ plaque deposition, which are the key features of AD, are significantly inhibited by the restoration of O-GlcNAcylation in AD model mice. Collectively, these results suggest that O-GlcNAcylation could be a potential therapeutic target for AD.

## Methods

### Animals and tamoxifen administration

Glast-CreERT2 (#012586), Nestin-CreERT2 (#016261), *Ogt*^floxp/floxp^ (#004860) and 5XFAD (AD) (#034840) mice were obtained from The Jackson Laboratory. The inducible *Ogt* conditional knockout mice (cKO) were produced by crossing *Ogt*^*floxp/floxp*^ mice with Glast-CreERT2 and Nestin-CreERT2 mice, respectively. All mice used were in the C57BL/6 genetic background and were bred in the animal center of Zhejiang University under 12-h light/12-h dark conditions with free access to food and water. A stock solution of Tamoxifen was prepared with a solution of corn oil at 37 °C with occasional vortexing until completely dissolved. Adult cKO male mice were administrated with 100 mg/kg tamoxifen and vehicle (i.p., 1 time/day for for 5 consecutive days), respectively.

### Isolation and culture of astrocytes

Adult astrocytes were isolated from adult male Ctrl and cKO mice according to the previous study with some modifications [63]. Adult mice were sacrificed by cervical dislocation and quickly decapitated. Hippocampal and cortical tissues were dissected in pre-chilled PBS and meninges were carefully removed under stereomicroscope. Single cells were isolated by 0.125% trypsin and 1000 U/mg papain in EBSS. After being washed with cold PBS, tissues were minced finely with a scalpel and pipetted, followed by a digestion with 0.125% trypsin for 15 min at 37 °C on a rotator. Precipitates were enzymatically dissociated by slowly spinning at 37 °C for 15 min with papain and reverse blending with 20 μl DNase I (50,000 U/ml). They were mechanically dissociated using a pipette. Cells isolated from 2 to 3 adult mice were pooled together, respectively and regarded as n = 1. Cell suspensions were neutralized with 2 ml DMEM with 10% FBS and filtered through 70 μM pore nylon meshes. After centrifuging at 1600 rpm for 3 min, cell pellets were resuspended with 3 ml of astrocyte growth medium (34% DMEM, 45% DMEM/F12, 20% FBS and 1% P/S) and 1 ml percoll density gradient media. The suspension was centrifuged at 1600 rpm for 20 min then washed once with 2 ml astrocyte growth medium and centrifuged at 1600 rpm for 3 min. Cells were resuspended with 4 ml of astrocyte growth medium and seeded on a PDL-coated 25 cm^2^-flask or slides in 24-well plates. Adult astrocytes were cultured at 37 °C in 5% CO_2_. The medium was changed completely on the 3rd day after plating, and half of the medium was changed once every 4 days. Flasks were shaken on a shaker at 260 rpm and 37 °C for 16–18 h once the plate coverage was over 95%. The purity of primary astrocytes was above 95% as determined by GFAP and Glast immunofluorescence staining.

The isolation and culture of primary astrocytes from postnatal day 1 (P1) pups were prepared as described previously with minor modifications [21]. Newborn pups were anesthetized with ice, and hippocampal and cortical tissues were dissected out. Tissues from two neonates were combined, washed with ice-cold PBS. After triturating by pipetting, tissue samples were treated with 0.25% trypsin–EDTA for 15 min at 37 °C on a rotator. After a quick spin, samples were treated with 0.25% trypsin–EDTA at 37 °C for another 12 min. Cell suspensions were added to 40 μl DNase I (50,000 U/ml) in 3 ml DMEM with 10% FBS and pipetted for 5 min. After filtration with a 70 μM pore nylon mesh and centrifugation at 1600 rpm for 6 min, cell pellets were resuspended with DMEM containing 10% FBS, 2 mM l-glutamine and 1% antibiotic–antimycotic then seeded on a PDL (5 μg/ml)-coated 25 cm^2^-flask. Astrocytes were cultured in a 5% CO_2_ incubator at 37 °C. 24 h later, medium was completely replaced, and 3/4 of the medium was replaced every 2 days after that. When ~ 95% confluency was reached, samples were shaken for 16–18 h (260 rpm) at 37 °C to purify them. The purity of astrocytes was determined by immunofluorescence staining and qRT-PCR. Astrocytes were passaged once with trypsin–EDTA for in vitro assays.

### Neurogenesis assay in vitro and in vivo

Adult (age of 7-week) Ctrl and cKO mice were injected 5-bromo-2′-deoxyuridine (BrdU) (50 mg/kg i.p., 6 times with an interval of 4 h) 8 weeks after the final administration of tamoxifen. 4 h post final BrdU injection, Ctrl and cKO mice were anesthetized with isoflurane and transcardially perfused with cold PBS followed by cold 4% paraformaldehyde (PFA). Brains were post-fixed, and completely dehydrated with 30% sucrose at 4 °C. Coronal sections were prepared with a cryostat (Leica) and sections containing the dentate gyrus of hippocampal and the lateral ventricles were picked up for immunostaining with BrdU and DCX antibodies.

For in vitro assay, adult neural stem/progenitor cells (aNSPCs) were isolated from the forebrain of Ctrl and *Ogt* cKO mice 8 weeks after final tamoxifen administration as previous described [7]. aNSPCs were isolated passaged about 4–5 times under the proliferating condition to achieve a stable and homogeneous state. Ctrl and *Ogt* cKO aNSPCs were cultured with DMEM/F12 medium containing 2% B27 (minus vitamin A), 20 ng/ml EGF, 20 ng/ml FGF-2, 2 mM l-Glutamine and 1% antibiotic–antimycotic. For differentiation, aNSPCs were cultured with DMEM/F12 medium containing containing 2% B27 (minus vitamin A), 2 mM l-glutamine, 1 μM retinotic acid, 5 μM forskolin and 1% antibiotic–antimycotic for 48 h.

### N2a cell culture and plasmids transfection

Neuroblastoma (N2a) cells were cultured with DMEM medium containing 10% FBS, 2 mM l-glutamine, 1% antibiotic–antimycotic in a 5% CO_2_ incubator at 37 °C, and medium was replaced every 24 h. The targeted plasmids and empty vectors were transfected with Lipo 2000 following the manufacturer’s instructions. Cells were collected for assays 48 h after transfection.

### Co-culture of neurons and astrocytes

The isolation and culture of hippocampal neurons from embryonic day 15 (E15) mice were prepared as described previously [78]. In brief, Hippocampi were dissected and digested by 0.125% trypsin at 37 °C for 10 min. 150,000 neurons were plated onto Poly-d-lysine (5 μg/ml) coated cell climbing slice with MEM containing 10% FBS, 1% l-Glu and 1% sodium pyruvate. 4 h later, the medium was replaced with neurobasal medium containing 0.25% l-Glu, 0.125% GlutaMax, 2% B27. Half of the medium was replaced every 3 days.

For astrocyte-neuron co-culture, astrocytes and neurons were cultured with neurobasal medium. 4 days later, neurobasal medium was replaced with astrocyte-conditioned medium (ACM) of Ctrl and *Ogt* cKO astrocytes, respectively, and cultured for another 3 (DIV7) or 6 (DIV10) days. Neurons were collected at DIV7 for cleaved caspase-3 immunostaining and Western blot assays. For morphology and sholl analysis, the medium was replaced at DIV7, and cells were collected at DIV10.

### Preparation and application of OSMI-4

Stock solution of Ogt inhibitor Ogt small molecule inhibitor-4 (OSMI-4) (5 mM) was prepared with DMSO. Ogt inhibitor was added to cultured medium at the final concentration of 20 μM for 72 h. Equal volume of PBS was added as control.

### Preparation and administration GlcNAc

N-Acetyl-D-glucosamine (GlcNAc) (MedChemExpress, #7512-17-6) was dissolved with nuclease free water. The details for in vitro and in vivo administration of GlcNAc can be found in figure legends.

### Immunofluorescence staining

Mice were anesthetized with isoflurane and transcardially perfused with cold PBS followed by cold 4% PFA. Brains were dissected and post-fixed with 4% PFA overnight. After completely dehydrating with 30% sucrose at 4 °C, brain samples were embedded with O.C.T and coronally sectioned at a thickness of 20-µm with a cryostat. The sections were stored in preservative solution at − 20 °C.

To perform staining, brain sections containing hippocampus regions were picked up and washed with PBS three times. For cultured cells, astrocytes were seeded on coverslips in 24-well plates and fixed with 4% PFA for 30 min followed by washing with PBS. Blocking solution containing 3% goat serum and 0.1% Triton X-100 in PBS were applied to tissue and cell samples at room temperature for 1 h, followed by incubation with primary antibodies at 4 °C overnight (the information for antibodies can be found in Additional file 13: Table S3). For BrdU immunostaining, samples were pretreated with 1N HCl for 30 min before applying the blocking solution. On the second day, samples were washed with PBS followed by incubation with fluorescence-conjugated secondary Alexa antibodies and counterstained with DAPI at room temperature for 1 h (the information for antibodies can be found in Additional file 13: Table S3). After washing, sections were mounted onto glass slides with mounting medium. Images were taken with an Olympus confocal microscope and analyzed with Image J software. All images were taken with identical settings under the same conditions. 4–5 sections per animal were adopted for analysis.

### Morphology and Sholl analysis

Captured images were inputted into Fiji and converted to 8-bit images. The Fiji plugin NeuronJ was used to manually trace dendritic arbors and analyze the length and branch number of individual cells. Traced images were then imported into Fiji and converted to 8-bit binary images. The soma center and neurite termination points were marked using the straight tool. Sholl analysis was carried out using the Fiji plugins Sholl Analysis [20] at 20 μm intervals to a maximum radius of 1000 μm, the number of intersections at each concentric circle was counted [34].

Three-dimension morphological analysis of GFAP^+^ astrocyte was performed with Imaris software (version 9.0.1). Confocal *z*-stack images (step size: 0.5 μm) of brain sections were taken with the 60× objective of an Olympus FV3000 confocal microscope. Three-dimensional images of single astrocyte were generated in Imaris Surpass view. The cell body and processes were segmented as the region of interest. The total length, the number of processes, the process volume, total process area and 3D sholl analysis were auto-analyzed with Imaris software.

### Golgi staining and quantitative analysis of dendrites and spine

Golgi staining was performed according to the manufacturer’s protocol (FD Neuro-Technologies, PK401). In brief, fresh mouse brains were placed in a Golgi-Cox solution and stored in the dark for 14 days followed by the incubation with 30% sucrose at 4 °C for 2 days. Coronal sections were cut to a thickness of 200 µm with a cryostat. The sections were collected, dehydrated in absolute alcohol, cleaned in xylene, and cover-slipped with a resinous medium in sequence. The dendritic branches and spine density of 6–8 pyramidal neurons per animal within CA1 region were imaged in Z-stack pattern with an Olympus light microscope and analyzed. Only well-impregnated cells with soma located in the middle plane of the section, without broken arbors and no overlap with neighboring cells were selected for analysis. A second-order segment in the apical dendrite was imaged to analyze the dendritic spine density, which was calculated by dividing the number of spines with the length of dendrite.

### Plasmids

Overexpression sequences were generated as follows. The mouse full-length p65-HA coding sequence (CDS) and partial 3′ and 5′ UTRs were PCR-amplified from mouse hippocampal cDNA and cloned into the pcDNA3.1 vector using the restriction sites XhoI by the ClonExpress II One Step Cloning Kit. p65 Ser or Thr to Ala substitution mutants, which included p65 S370A (Ser-370 mutated to Ala), S374A, S384A, S396A were created by site-directed mutagenesis and the used primers can be found in Additional file 14: Table S4.

### Western blot

Cortices and hippocampi were dissected on ice and quick frozen in liquid nitrogen before total protein extraction. Tissues were triturated with cold RIPA Lysis Buffer on ice and transferred to a microfuge tube, followed by centrifugation at 4 °C for 30 min at 12,000 rpm. Cell pellets were collected and the total proteins were lysed with cold RIPA Lysis Buffer on ice and then centrifuged at 4 °C for 30 min at 12,000 rpm. Supernatants were collected and quantified by BCA protein assay. Nuclear and cytoplasmic proteins were extraction with KIT.

Proteins were subjected to sodium dodecyl sulfate–polyacrylamide gel electrophoresis (SDS-PAGE) at different concentrations of separating gels according to molecular weights of target proteins, and then transferred onto nitrocellulose membranes. Membranes were blocked with 5% non-fat milk or 5% BSA in TBST at room temperature for 1 h and incubated with primary antibodies at 4 °C overnight. After 3 washes with TBST, membranes were incubated with HRP-labeled secondary antibodies at room temperature for 1 h. Membranes were washed 3 times with TBST and incubated with UltraSignal ECL. Protein bands were visualized by a Molecular Imager Imaging System and band intensities were measured by Adobe Photoshop software.

### Enzyme-linked immunosorbent assays (ELISA)

Hippocampal tissues were homogenized in ice-cold lysis buffer (500 μl, 20 mM Tris–HCl, 150 mM NaCl, pH7.4, 1% Triton X-100, 1 mM EDTA, 1× protease inhibitor cocktail) and centrifuged at 14,000×*g* for 15 min at 4 °C and the supernatants were collected. Enzyme-Linked Immunosorbent Assays (ELISA) for IL-1β (Thermo Fisher Scientific, 88-7013-88), TNF-α (Thermo Fisher Scientific, 88-7324-88) and UDP-GlcNAc (JINHENGNUO, JHN80816) were performed with 100 μl cell and tissue supernatants using ELISA kits according to the manufacturer’s instructions.

### Click-iT O-GlcNAcylation analysis and mapping glycosylation sites

Analysis of p65 O-GlcNAcylation was conducted as described [81]. Briefly, O-GlcNAcylated proteins in cell lysates were first labeled with GalNAz using the Click-iT O-GlcNAc Enzymatic Labeling System and conjugated with an alkyne-biotin compound using the Click-iT Protein Analysis Detection System. Control experiments in the absence of the enzyme GalT or UDP-GalNAz were carried out in parallel. Labeled proteins were then precipitated, resolubilized, neutralized, and further incubated with streptavidin beads at 4 °C overnight. After extensive washing steps, the bound protein fractions were eluted in the boiling elution buffer containing 50 mM Tris–HCl pH 6.8, 2.5% SDS, 100 mM DTT, 10% glycerol, and 20 mM biotin. Immunoblotting was carried out using anti-p65 antibody.

Flag-tagged p65 and HA-tagged OGT were co-expressed in N2a cells for 48 h. Flag-tagged p65 was immunoprecipitated from the cell lysates using anti-Flag-M2 magnetic beads and eluted in a buffer containing 4% SDS and 100 mM Tris–HCl pH 8.0. After separation and staining in 8–15% Bis–Tris gels, the p65 protein band was excised and digested with trypsin and chymotrypsin. The extracted mixtures were purified by reverse-phase HPLC (Agilent 1100) using a gradient of 5–30% buffer B (100% MeCN) over 20 min at 4 ml/min and a constant flow of buffer A (0.5% aqueous AcOH). Eluted fractions between 5 and 12 min were manually collected, pooled, lyophilized, and analyzed using nanoLC-LTQ-CID/ETD-MS. Proteome Discovery (MASCOT search engine, version 1.3) was used for data search with O-GlcNAc (Ser/Thr) set as a variable modification.

### Immunoprecipitation/co-immunoprecipitation (IP/co-IP)

Primary P1 astrocytes were prepared for endogenous IP and Co-IP. N2a cells transfected with Ogt-Flag and NF-κB p65-HA overexpression plasmids were prepared for exogenous IP/Co-IP. For IP, cells were lysed by RIPA Lysis Buffer and lysates were filled up to 300 μl with IP Lysis Buffer (50 mM Tris–HCl, 0.5% Triton-X, 150 mM KCl, 1 mM EDTA in ddH_2_O). For endogenous assay, lysates were blocked with 10 μl Protein A agarose beads at 4 °C for 2 h with rotation. 3 μl of primary antibody or rabbit IgG were added to the supernatant and incubated with rotation overnight at 4 °C. 30 μl of Protein A agarose beads were conjugated with antibody at 4 °C for 6–8 h with rotation. For exogenous assays, HA/Flag-beads with conjugated antibodies were added to the lysates directly at 4 °C overnight. Unconjugated protein was removed from beads by 3 washing times with IP Wash Buffer (50 mM Tris–HCl, 0.1% Triton-X, 150 mM KCl, 1 mM EDTA in ddH_2_O) at 4 °C for 10 min. Target proteins were eluted from beads in 1× loading buffer at 100 °C for 5 min, and lysates before IP were used as the input. Proteins were detected by Western blot.

For co-IP assays, N2a cells and P0 astrocytes were crosslinked with 1% formaldehyde for 10 min, respectively, and the reaction was terminated with 125 mM glycine for 5 min at room temperature on a shaker at 40 rpm. Crosslinked cells were lysed with IP Lysis Buffer to avoid breaking up protein–protein interactions. co-IP assays were preformed similar to IP assay described above.

### Total RNA extraction and quantitative real-time PCR (qRT-PCR)

Total RNA from cells and tissues were extracted by TRIzol reagent according to the manufacture’s protocol, and the concentration was determined with a Nanodrop spectrophotometer 2000 (Thermo Fisher Scientific). 0.5 µg total RNA was used for reverse transcription, and standard real-time qPCR assays were performed using SYBR Green (Vazyme) in triplicate. The results were analyzed using the ^ΔΔ^Ct method. Samples from 3 to 5 animals per group were analyzed. The primers for qRT-PCR are shown in Additional file 14: Table S4.

### RNA-seq and data analysis

All samples used for the cDNA library were assessed by NanoDrop 2000 (Thermo Fisher Scientific), and the RNA integrity value (RIN) was determined with the RNA Nano 6000 Assay Kit of the Bioanalyzer 2100 system (Agilent Technologies Inc.). A total amount of 3 µg of RNA per sample was used as input for each RNA sample preparation. Sequencing libraries were generated following the manufacturer’s recommendations, and index codes were added to attribute sequences to each sample using NEBNext® UltraTM RNA Library Prep Kit for Illumina® (NEB). Second strand cDNA synthesis was subsequently performed using DNA Polymerase I and RNase H. Remaining overhangs were converted into blunt ends via exonuclease/polymerase activities. The NEBNext Adaptor with hairpin loop structure was ligated to prepare for hybridization after adenylation of the 3′ ends of DNA fragments. The library fragments were purified with AMPure XP system (Beckman Coulter) to preferentially select cDNA fragments of 250–300 bp. Then, 3 µl USER Enzyme (NEB) was used with size-selected, adaptor-ligated cDNA at 37 °C for 15 min, followed by 5 min at 95 °C before PCR. Then PCR was performed with Phusion High-Fidelity DNA polymerase, Universal PCR primers and Index (X) Primer. Finally, PCR products were purified using AMPure XP system (Beckman Coulter), and library quality was assessed on the Agilent Bioanalyzer 2100 system (Agilent Technologies Inc.). Subsequently, the clustering of the index-coded samples was performed on a cBot Cluster Generation System using TruSeq PE Cluster Kit v3-cBot-HS (Illumina) according to the manufacturer’s instructions. After cluster generation, the library preparations were sequenced on an Illumina Hiseq platform (Illumina NovaSeq6000), and 125 bp/150 bp paired-end reads were generated.

Raw reads of fastq format were processed. In this step, clean reads were obtained by removing reads containing adapter, reads containing ploy-N and low-quality reads. The retained clean reads were aligned to the *Mus musculus* reference genome mm10. To quantify the gene expression level, feature counts v1.5.0-p3 was used to count the read numbers mapped to each gene. FPKM (expected number of Fragments Per Kilobase of transcript sequence per Millions base pairs sequenced) of each transcript was calculated based on the length of the gene and the count of reads mapped to this gene.

### Gene ontology analysis

Gene ontology (GO) analysis was performed using the DAVID database, as described previously [8]. Each enriched GO function term is represented by a node, and the node size is proportional to the number of genes in its corresponding function term in the enrichment maps. Similar GO functions are categorized as one cluster. The function term and the number of genes in each cluster are labelled.

### Behavioral tests

The Morris water maze (MWM) was performed as previously described [42]. MWM was performed in a round, water-filled tub (120 cm in diameter). MWM was divided into two stages: training and testing. For the training stage, an invisible escape platform (10 cm in diameter) was located in the same spatial location 1 cm below the water surface independent of a subject’s start position on a particular trial. Mice were placed in the water maze from 4 different starting positions (NE, NW, SE, SW). In this way, each animal would be able to determine the platform’s location, and each animal was given 4 trials/day for 4 days. 24 h after the final training trial, a probe trial was performed, during which the platform was removed and the time of swimming in the quadrant that previously placed the escape platform during task acquisition was measured over 60 s. All trials were videotaped and analyzed with MazeScan software (Acimetrics).

The Novel Object recognition (NOR) task test was performed as described previously [4]. Two sample objects in one environment were used to examine learning and memory with 24 h delays. In this test, mice were placed in a white plastic box (60 cm × 60 cm × 30 cm) with illumination, monitored by an overhead video camera and analyzed by an automated tracking system (San Diego Instruments, CA). On the first day, two identical objects (small cylindrical iron columns with a diameter of 5 cm and a height of 5 cm), termed as ‘old’, were placed 15 cm from the walls in the North–South orientation, and a mouse was placed at the mid-point of the wall opposite to the sample objects. After exploring the objects for 10 min, mice were put back in their home cage. 24 h later, one of the ‘old’ objects used for the memory acquisition was replaced with a ‘novel’ object (a small cylindrical iron cone with a diameter of 5 cm and a height of 5 cm) similar to the ‘old’ one. The mouse was again placed in the chamber for 10 min to explore the objects and the time spent exploring the novel and old object was assessed.

Y maze spontaneous alternation and spatial novelty preference test with three identical, opaque arms at 120° angles from each other, as previously described [12]. The arms were 35 cm long, 8 cm wide and 8 cm high, allowing the mice to see distal spatial cues. The continuous spontaneous alternation testing is based on the natural tendency of rodents to explore a novel environment. The mice were placed in the Y maze, facing the wall of one randomly chosen arm and could freely explore the three arms of the Y maze for 8 min. Typically, mice explore the least recently visited arm and tend to alternate between the three arms. For efficient alternation, mice rely on their spatial working memory [66]. Every arm entry was recorded by automated tracking system (San Diego Instruments, CA) in order to calculate the percentage of alternation. An entry was recorded when all four limbs were within the arm. The number of arm entries and total distance were used as measures for locomotor activity, while the spontaneous alternation percentage was used as a measure of spatial working memory. To calculate the percentage, the total number of alternations (i.e., every time a mouse explored the three arms consecutively) was divided by the total possible alternations (i.e., the total number of arm entries minus 2) and multiplied by 100.

In Y maze spatial novelty preference test, one of the three arms was closed and the mice could explore the other two arms for 10 min (training phase). Next, the mice were transferred to their holding cage for 2 h. Subsequently, they were placed into the start arm and could explore all three arms of the maze for 5 min (test phase). During these 5 min, the time and distance and number of entries in each arm were recorded by automatic tracking system (San Diego Instruments, CA) and those in the novel arm (i.e., previously closed during the training phase) were used as measures for spatial short-term memory.

The passive avoidance task was performed as previously described [27], with minor modifications. The apparatus consists of a two-chambered light–dark shuttle box (20.3 cm height × 21.3 cm width × 15.9 cm depth; ENV-010MC, Med Associates, USA) interconnected with a guillotine door. The experimental protocol consisted of two sessions; the training and the retention test session, conducted 24 h apart. During the training session, the animals were individually placed into the light compartment of the apparatus and explored the light compartment for a 30-s adaptation period with the door between the light and dark compartments closed. After the adaptation period, the door between the two compartments was automatically raised and mice were given an additional 5 min to explore the compartment during which the latency to enter the dark compartment was measured. Upon entering the dark compartment, the guillotine door was closed and 3 s later an inescapable foot-shock (0.5 mA, 2 s duration) was delivered through the grid floor. Thirty sec after the foot-shock, animals were immediately returned to their home cage. On the retention test day (24 h following training) mice were placed into the light compartment of the apparatus, and after a 30-s delay the door between the two compartments was raised. The latency to enter the dark compartment was automatically measured by the Instruments software. The trial was terminated after 5 min when the animal did not cross into the dark compartment.

### Statistical analysis

Data are presented as the means ± SEM and were analyzed with GraphPad Prism software. Unpaired Student’s t test was used to determine the difference between two groups; one-way ANOVA analysis followed by Tukey’s multiple-comparison test (used for 1 variable with normal distribution sample) and two-way ANOVA analysis followed by Sidak’s multiple-comparison test (used for multiple variables) was used to determine differences between multiple groups, respectively. P < 0.05 was considered statistically significant. The details for the number of samples used can be found in Figure legends.

## Supplementary Information

**Additional file 1: Figure S1.** *Ogt* deficient astrocytes showed the reduced levels of Ogt and O-GlcNAcylation.Representative images of GFAP, Aldh1l1, Glast, Iba1, and Tuj1 immunostaining with cultured adult Ctrl astrocytes. Scale bar, 50 μm.Quantification results show that the percentage of GFAP^+^, Glast^+^ and Aldh1l1^+^ cells are all around 97%, the percentage of Tuj1^+^ cells is around 2%, and the percentage of Iba1^+^ cells is around 3%. n = 3 independent experiments. Values represent mean ± SEM.WB assayand quantification results showed that P1 and adult astrocytes showed the decreased levels of Ogtand Oga, but the increased Ogt/Ogaand O-GlcNAcylation levelcompared with cortical and hippocampal neurons. n = 3 independent experiments for each group. Values represent mean ± SEM; *p < 0.05, **p < 0.01, ***p < 0.001; one-way ANOVA analysis followed by Tukey’s multiple-comparison test, *F* = 38.5 for, *F* = 333.8 for, *F* = 51.81 for, *F* = 418.4 for. C: cortical neurons. H, hippocampal neurons; P1, postnatal day 1; Adult, postnatal 7-week.Schematic illustration of tamoxifen administration strategy. AdultGlast-CreERT2::Ogt^floxp/Y^ mice were intraperitoneallyadministrated with tamoxifenand corn oil, respectively. Eight weeks after tamoxifen administration, mice were sacrificed for assays.qRT-PCR results showed that mRNA level of *Ogt* significantly decreased in adult cKO astrocytes compared with Ctrl astrocytes. n = 3 independent experiments. Values represent mean ± SEM; *p < 0.05, **p < 0.01, ***p < 0.001; unpaired Student’s t-test.WB assayand quantification results showed that the protein levels of Ogtand O-GlcNAcylationsignificantly decreased in adult cKO astrocytes compared with Ctrl astrocytes. The level of Oga was also decreased in cKO cells. n = 3 independent experiments. Values represent mean ± SEM; *p < 0.05, **p < 0.01, ***p < 0.001; unpaired Student’s t-test.Representative images of GFAP-Ogtand Glast-O-GlcNAcylationimmunostaining with cultured adult Ctrl and cKO astrocytes. Scale bar, 50 μm.Representative images of GFAP-Ogtand Glast-O- GlcNAcylationimmunostaining with the brain sections of adult Ctrl and cKO mice. Scale bar, 20 μm.

**Additional file 2: Figure S2.** Astrocytic *Ogt* deficiency does not affect microglia, and *Ogt* cKO in neural stem/progenitor cells does not induce inflammation in vivo.Three-dimensionalview of astrocytesand microgliaimmunostaining in the hippocampal regions of Ctrl and cKO mice. Scale bar, 20 μm.Quantification results showed that the level of Iba1 fluorescence intensityand the number of Iba1^+^ cellsshowed no difference in the hippocampus region between Ctrl and cKO mice. n = 4 mice per genotype. Values represent mean ± SEM; *p < 0.05, **p < 0.01, ***p < 0.001; unpaired Student’s t-test.Representative images of Iba1 immunostaining in the hippocampal regions of Ctrl and cKO mice. Scale bar, 20 μm.Sholl analysis showed no significant difference in the number of neurite intersections per radius, the number of neurites per cell, and the total length of neuritesof microglia in the hippocampal regions of *Ogt* cKO mice compared with Ctrl mice. 12 cells were picked up per animal and total 36 cells from 3 mice were analyzed per group; Values represent mean ± SEM; *p < 0.05, **p < 0.01, ***p < 0.001; two-way ANOVA analysis followed by Sidak's multiple-comparison test for, *F* = 5.062; unpaired Student’s t-test for.qRT-PCR results showed that mRNA level of Iba1 in the hippocampus showed no difference between Ctrl and cKO mice. n = 5 mice per genotype. Values represent mean ± SEM; *p < 0.05, **p < 0.01, ***p < 0.001; unpaired Student’s t-test.

**Additional file 3: Figure S3.** Astrocytic *Ogt* deficiency impairs adult neurogenesis, and *Ogt* cKO in neural stem/progenitor cells does not induce inflammation in vivo.Representative images of DCX and BrdU immunostaining in the subgranular zoneof the hippocampus of Ctrl and *Ogt* cKO mice. Scale bar, 100 μm.Quantification results showed that cKO mice had the reduced number of BrdU^+^ cellsand DCX^+^cells, but a similar percentage of BrdU^+^DCX^+^/BrdU^+^in the SGZ of Ctrl and *Ogt* cKO mice. mice were injectedwith BrdU and sacrificed 1-day post the final BrdU administration. n = 4 mice per genotype. Values represent mean ± SEM; *p < 0.05, **p < 0.01, ***p < 0.001; unpaired Student’s t-test.Representative images of DCX and BrdU immunostaining in the subventricular zoneof the lateral ventricles of Ctrl and *Ogt* cKO mice. Scale bar, 100 μm.Quantification results showed that cKO mice had the reduced number of of BrdU^+^ cellsand DCX^+^cells, but a similar percentage of BrdU^+^DCX^+^/BrdU^+^in the SVZ of Ctrl and *Ogt* cKO mice. mice were injectedwith BrdU and sacrificed 1-day post the final BrdU administration. n = 4 mice per genotype. Values represent mean ± SEM; *p < 0.05, **p < 0.01, ***p < 0.001; unpaired Student’s t-test.WB assayand quantification resultsshowed that the protein level of Ogt and O-GlcNAcylation were not affected in proliferating adult neural stem/progenitor cellsof Ctrl and *Ogt* cKO mice. n = 4 independent experiments. Values represent mean ± SEM; *p < 0.05, **p < 0.01, ***p < 0.001; unpaired Student’s t-test.qRT-PCR results showed that under proliferating condition, Ctrl and cKO aNSPCs showed no difference in mRNA levels of *Ogt*, *Sox2*and *Nestin*. n = 4 independent experiments. Values represent mean ± SEM; *p < 0.05, **p < 0.01, ***p < 0.001; unpaired Student’s t-test.qRT-PCR results showed that under differentiation condition, Ctrl and cKO aNSPCs showed no difference in mRNA levels of mRNA level of *Ogt*, *S100β*and *Map2*. n = 4 independent experiments. Values represent mean ± SEM; *p < 0.05, **p < 0.01, ***p < 0.001; unpaired Student’s t-test.Representative images of GFAP and Iba1 immunostaining in the hippocampus of Ctrland *NestinCreERT2*::*Ogt*^loxp/Y^adult mice. Scale bar, 100 μm.

**Additional file 4: Figure S4.** Reactive astrocytes impair hippocampal neuronal cells and cognition of mice.ELISA results showed that the levels of IL-1βand TNF-αremarkably increased in the supernatants of cKO astrocyte-conditioned medium. n = 3 independent experiments. Values represent mean ± SEM; *p < 0.05, **p < 0.01, ***p < 0.001; unpaired Student’s t-test.Representative images of Tuj1 immunostaining with hippocampal neurons at day in vitro 10cultured with neurobasal medium, Ctrl and cKO astrocyte-conditioned medium, respectively. Scale bar, 20 μm.Sholl analysis showed that hippocampal neurons cultured with cKO ACM displayed the overall decrease in the number of dendritic intersections per radius, the number of dendrites per celland total length of dendritesat DIV 10 compared with NB- and Ctrl ACM-cultured cells. Values represent mean ± SEM; *p < 0.05, **p < 0.01, ***p < 0.001; n = 36 neurons from 3 independent experiments for each group; two-way ANOVA analysis followed by Sidak’s multiple-comparison test for, *F* = 353.5; one-way ANOVA analysis followed by Tukey’s multiple-comparison test for, *F* = 87.52 for, *F* = 168.7 for.Representative images of Tuj1 and activated-Caspase3immunostaining with hippocampal neuronscultured with neurobasal medium, Ctrl and cKO ACM, respectively. Scale bar, 50 μm.Quantification results showed that hippocampal neurons cultured with cKO ACM displayed the increased percentage of activated-Caspase3^+^ cells compared to that with NB- and Ctrl ACM-cultured cells. n = 6 independent experiments. Values represent mean ± SEM; *p < 0.05, **p < 0.01, ***p < 0.001; one-way ANOVA analysis followed by Tukey’s multiple-comparison test, *F* = 86.5.WB assayand quantification resultsshowed that the protein level of aCaspase3 significantly increased in hippocampal neurons cultured with cKO ACM compared to cell cultured with NB and Ctrl ACM, respectively. n = 3 independent experiments. Values represent mean ± SEM; *p < 0.05, **p < 0.01, ***p < 0.001; one-way ANOVA analysis followed by Tukey’s multiple-comparison test, *F* = 42.74.Schematic illustration of tamoxifen administration and behavioral tests strategy. adult Glast-CreERT2::Ogt^floxp/Y^mice were intraperitoneally injected with tamoxifenand corn oil, respectively. Eight weeks after tamoxifen administration, behavioral tests were performed.Representative images of the swimming path of Ctrl and cKO mice during the probe trial in Morris Water Maze test. The red circle represents the position of platform.Adult Ctrl and cKO mice showed no difference for the length of swimming pathand average swimming speedduring the probe trial test. n = 10 mice per group. Values represent mean ± SEM; *p < 0.05, **p < 0.01, ***p < 0.001; unpaired Student’s t-test.Y maze spontaneous alternation test results showed no difference for the number of arm entriesand total distancebetween Ctrl and cKO mice. n = 8 mice per group. Values represent mean ± SEM; *p < 0.05, **p < 0.01, ***p < 0.001; unpaired Student’s t-test.

**Additional file 5: Figure S5.** Restoration of O-GlcNAcylation inhibits astrocyte activation and inflammation, and improves cognitive function of *Ogt* deficient mice.Schematic illustration of tamoxifen and GlcNAc administration strategy. AdultGlast-CreERT2::Ogt^floxp/Y^ mice were intraperitoneally injected with tamoxifenand corn oil, respectively. 6 weeks after tamoxifen administration, Ctrl and cKO mice were injected with salineand GlcNAcfor 14 days, and then the mice were sacrificed for assays.Representative images of GFAP and IL-1β immunostaining with brain sections of Ctrl and cKO mice treated with salineand GlcNAc, respectively. Scale bar, 20 μm.Schematic illustration of tamoxifen, GlcNAc administration and behavioral test strategy. AdultGlast-CreERT2::Ogt^floxp/Y^ mice were intraperitoneally injected with tamoxifenand corn oilfor, respectively. Six weeks after tamoxifen administration, Ctrl and cKO mice were injected with salineor GlcNAcfor 14 days, respectively, and behavioral tests were performed. Saline and GlcNAc was continuously injected during the behavioral tests.Mice from the four group showed no difference in the percentage of time spent exploring two identical objectsand during the training period in novel object recognition task test. n = 6 mice per group; Values represent mean ± SEM; *p < 0.05, **p < 0.01, ***p < 0.001; two-way ANOVA analysis followed by Sidak's multiple-comparison test, *F* = 0.3952.Schematic illustration of novel object recognition task and heatmap image of each animal showing the distribution of exploring time of 4 groups mice during the testing trial. n = 6 mice per group.

**Additional file 6: Figure S6.** Restoration of O-GlcNAcylation improves cognitive function.Mice showed no difference in the number of arm entries, total distancein Y maze spontaneous alternation test between four groups. n = 6 mice per group. Values represent mean ± SEM; *p < 0.05, **p < 0.01, ***p < 0.001; two-way ANOVA analysis followed by Sidak's multiple-comparison test for, *F* = 0.1623; one-way ANOVA analysis followed by Tukey’s multiple-comparison test for, *F* = 0.5806.Schematic Illustration of Y spontaneous alternation test and representative heat map images of mice during Y maze spontaneous alternation test.Representative images of the swimming path during the probe trial of Morris Water Maze test. The red circle represents the platform.The average swimming speedand total swimming path lengthshowed no difference in all four groups during the probe trial test. n = 6 mice per group; Values represent mean ± SEM; *p < 0.05, **p < 0.01, ***p < 0.001; one-way ANOVA analysis followed by Tukey’s multiple-comparison test, *F* = 0.3744 forand *F* = 0.3702 for.

**Additional file 7: Figure S7.** Ogt interacts with NF-κB and catalyzes the O-GlcNAcylation of NF-κB.Schematic illustration of O-GlcNAcylation sites of p65 on S374, S370, S384 and S396 in N2a cells identified by MS/MS analysis. nanoLC-LTQ-CID was used to mapping the sites of O-GlcNAcylation on p65. The matched fragment ions are labeled in y and b.IP-WB assayand quantification results show that mutation of single potential O-GlcNAcylation sites led to a significant decrease in the O-GlcNAcylation level of p65in N2a cells, and only the mutation of S384 induced a significant increase of p-p65. n = 3 independent experiments. Values represent mean ± SEM; *p < 0.05, **p < 0.01, ***p < 0.001; one-way ANOVA analysis followed by Tukey’s multiple-comparison test, *F* = 51.62 forand *F*  = 74.06 for.IP followed by WB assay results showed the level of GSK3β showed no difference in the hippocampus of Ctrl and cKO mice, and the interaction between p65 and GSK3β was significantly increased in the hippocampus of Ctrl and cKO mice. n = 3 independent experiments. Values represent mean ± SEM; *p < 0.05, **p < 0.01, ***p < 0.001; unpaired Student’s t-test.co-IP followed by WB assay showed no direct interaction between Gsk3β and Ogt.

**Additional file 8: Figure S8.** RNA-seq data analysis shows that *Ogt* deficiency activates NF-κB signaling pathway in astrocytes.Heatmap illustrating the altered transcriptome of cKO astrocytes group compared to Ctrl astrocytes. Red color, up-regulated genes; blue color, down-regulated genes. Two biological replicate samples were used for sequencing in each group.Gene ontologyCircle visualization of differentially expressed genesenriched biological process terms in cKO astrocytes including inflammatory response, cytokine signaling, NF-κB signaling, axon development and learning and memory. The outer circle showed the log2 fold changeof the genes in each category, and the height of the inner bar plot indicated the significance level of the GO term), and the color represents the Z score.WB assayand quantification results showed that *Ogt* deficiency did not affect the level of total p65, but significantly increased the protein level of p-p65in cultured astrocytes. n = 3 independent experiments. Values represent mean ± SEM; *p < 0.05, **p < 0.01, ***p < 0.001; unpaired Student’s t-test.WB assaysand quantification results showed that *Ogt* deficiency significantly reduced the level of p65 in cytoplasmand significantly increased the level of p-p65 in nucleus. n = 3 independent experiments. Values represent mean ± SEM; *p < 0.05, **p < 0.01, ***p < 0.001; unpaired Student’s t-test.

**Additional file 9: Figure S9.** Restoration of O-GlcNAcylation inhibits astrocyte activation and inflammation, and improves cognitive function of *Ogt* deficient mice.Schematic illustration of GlcNAc administration strategy. 2.5-month-old Ctrl and AD mice were administrated with salineand GlcNAcfor 6 weeks, and mice were sacrificed for assays.Representative images of O-GlcNAcylation and Iba1 immunostaining with brain sections of Ctrl and cKO mice treated with salineand GlcNAc, respectively. Scale bar, 100 μm.Quantification results show that the level of of O-GlcNAcylation fluorescence intensitysignificantly decreased in the hippocampus region of AD mice compared to Ctrl mice, which was significantly increased by GlcNAc administration. The intensity of Iba1 fluorescence was also significantly reduced after GlcNAc administration. n = 5 mice per group. Values represent mean ± SEM; *p < 0.05, **p < 0.01, ***p < 0.001; one-way ANOVA analysis followed by Tukey’s multiple-comparison test, *F* = 10.64) for, *F* = 8.674.WB assaysand quantification results showed that the levels of O-GlcNAcylation, Ogt, Ogasignificantly decreased, but the level of APPsignificantly increased in AD astrocytes compared with Ctrl astrocytes. n = 4 independent experiments. Values represent mean ± SEM; *p < 0.05, **p < 0.01, ***p < 0.001; unpaired Student’s t-test.IP-WBshowed the reduced levels of O-GlcNAcylationand Ogt, but the levels of p65and GSK3βwere not affected in the hippocampus of Ctrl and AD mice. n = 4 mice per group. Values represent mean ± SEM; *p < 0.05, **p < 0.01, ***p < 0.001; unpaired Student’s t-test.Quantification results of IP inshowed that the decreased O-GlcNAcylation on p65, the decreased interaction between p65 and Ogt, and the increased interaction between p65 and GSK3βin the hippocampus of AD mice compared to Ctrl mcie. n = 4 mice per group. Values represent mean ± SEM; *p < 0.05, **p < 0.01, ***p < 0.001; unpaired Student’s t-test.

**Additional file 10: Figure S10.** The original images of all western blot assays.

**Additional file 11: Table S1.** The list of proteins interacted with p65 in Ctrl and cKO hippocampal tissues.

**Additional file 12: Table S2.** The DEGs in cKO astrocyte.

**Additional file 13: Table S3.** Reagents and experimental model resources.

**Additional file 14: Table S4.** The list of primers used for qPCR, plasmids construction and and genotyping.

## Data Availability

The accession number for the RNA-seq data reported in this paper is GEO: GSE1693471.

## References

[1] Allen NJ, Lyons DA (2018). Glia as architects of central nervous system formation and function. Science.

[2] Allison DF, Wamsley JJ, Kumar M, Li D, Gray LG, Hart GW, Jones DR, Mayo MW (2012). Modification of RelA by O-linked *N*-acetylglucosamine links glucose metabolism to NF-kappaB acetylation and transcription. Proc Natl Acad Sci USA.

[3] Araujo L, Khim P, Mkhikian H, Mortales CL, Demetriou M (2017). Glycolysis and glutaminolysis cooperatively control T cell function by limiting metabolite supply to N-glycosylation. Elife.

[4] Bevins RA, Besheer J (2006). Object recognition in rats and mice: a one-trial non-matching-to-sample learning task to study ‘recognition memory’. Nat Protoc.

[5] Block ML, Zecca L, Hong JS (2007). Microglia-mediated neurotoxicity: uncovering the molecular mechanisms. Nat Rev Neurosci.

[6] Brambilla R, Bracchi-Ricard V, Hu WH, Frydel B, Bramwell A, Karmally S, Green EJ, Bethea JR (2005). Inhibition of astroglial nuclear factor kappaB reduces inflammation and improves functional recovery after spinal cord injury. J Exp Med.

[7] Chen J, Dong X, Cheng X, Zhu Q, Zhang J, Li Q, Huang X, Wang M, Li L, Guo W (2021). Ogt controls neural stem/progenitor cell pool and adult neurogenesis through modulating Notch signaling. Cell Rep.

[8] Chen J, Zhang YC, Huang C, Shen H, Sun B, Cheng X, Zhang YJ, Yang YG, Shu Q, Yang Y (2019). m(6)A regulates neurogenesis and neuronal development by modulating histone methyltransferase Ezh2. Genom Proteom Bioinform.

[9] Cheng J, Wu Y, Chen L, Li Y, Liu F, Shao J, Huang M, Fan M, Wu H (2020). Loss of O-GlcNAc transferase in neural stem cells impairs corticogenesis. Biochem Biophys Res Commun.

[10] Chung WS, Allen NJ, Eroglu C (2015). Astrocytes control synapse formation, function, and elimination. Cold Spring Harb Perspect Biol.

[11] Cildir G, Low KC, Tergaonkar V (2016). Noncanonical NF-kappaB signaling in health and disease. Trends Mol Med.

[12] De Bundel D, Schallier A, Loyens E, Fernando R, Miyashita H, Van Liefferinge J, Vermoesen K, Bannai S, Sato H, Michotte Y (2011). Loss of system x(c)- does not induce oxidative stress but decreases extracellular glutamate in hippocampus and influences spatial working memory and limbic seizure susceptibility. J Neurosci.

[13] de Jesus T, Shukla S, Ramakrishnan P (2018). Too sweet to resist: control of immune cell function by O-GlcNAcylation. Cell Immunol.

[14] Dela Justina V, Goncalves JS, de Freitas RA, Fonseca AD, Volpato GT, Tostes RC, Carneiro FS, Lima VV, Giachini FR (2017). Increased O-linked *N*-acetylglucosamine modification of NF-KappaB and augmented cytokine production in the placentas from hyperglycemic rats. Inflammation.

[15] Dias WB, Hart GW (2007). O-GlcNAc modification in diabetes and Alzheimer’s disease. Mol BioSyst.

[16] Diniz LP, Matias I, Siqueira M, Stipursky J, Gomes FCA (2019). Astrocytes and the TGF-beta1 pathway in the healthy and diseased brain: a double-edged sword. Mol Neurobiol.

[17] Dong H, Liu Z, Wen H (2022). Protein O-GlcNAcylation regulates innate immune cell function. Front Immunol.

[18] Douglass JD, Dorfman MD, Fasnacht R, Shaffer LD, Thaler JP (2017). Astrocyte IKKbeta/NF-kappaB signaling is required for diet-induced obesity and hypothalamic inflammation. Mol Metab.

[19] Fan J, Guo F, Mo R, Chen LY, Mo JW, Lu CL, Ren J, Zhong QL, Kuang XJ, Wen YL (2023). O-GlcNAc transferase in astrocytes modulates depression-related stress susceptibility through glutamatergic synaptic transmission. J Clin Invest.

[20] Ferreira T, Ou Y, Li S, Giniger E, van Meyel DJ (2014). Dendrite architecture organized by transcriptional control of the F-actin nucleator Spire. Development.

[21] Foo LC, Allen NJ, Bushong EA, Ventura PB, Chung WS, Zhou L, Cahoy JD, Daneman R, Zong H, Ellisman MH (2011). Development of a method for the purification and culture of rodent astrocytes. Neuron.

[22] Giovannoni F, Quintana FJ (2020). The role of astrocytes in CNS inflammation. Trends Immunol.

[23] Habib N, McCabe C, Medina S, Varshavsky M, Kitsberg D, Dvir-Szternfeld R, Green G, Dionne D, Nguyen L, Marshall JL (2020). Disease-associated astrocytes in Alzheimer’s disease and aging. Nat Neurosci.

[24] Hanover JA, Krause MW, Love DC (2012). Bittersweet memories: linking metabolism to epigenetics through O-GlcNAcylation. Nat Rev Mol Cell Biol.

[25] Harada K, Kamiya T, Tsuboi T (2015). Gliotransmitter release from astrocytes: functional, developmental, and pathological implications in the brain. Front Neurosci.

[26] Hart GW, Housley MP, Slawson C (2007). Cycling of O-linked beta-*N*-acetylglucosamine on nucleocytoplasmic proteins. Nature.

[27] Hefco V, Yamada K, Hefco A, Hritcu L, Tiron A, Nabeshima T (2003). Role of the mesotelencephalic dopamine system in learning and memory processes in the rat. Eur J Pharmacol.

[28] Hill SA, Fu M, Garcia ADR (2021). Sonic hedgehog signaling in astrocytes. Cell Mol Life Sci.

[29] Howerton CL, Morgan CP, Fischer DB, Bale TL (2013). O-GlcNAc transferase (OGT) as a placental biomarker of maternal stress and reprogramming of CNS gene transcription in development. Proc Natl Acad Sci USA.

[30] Hsiao HY, Chen YC, Chen HM, Tu PH, Chern Y (2013). A critical role of astrocyte-mediated nuclear factor-kappaB-dependent inflammation in Huntington's disease. Hum Mol Genet.

[31] Johswich A, Longuet C, Pawling J, Abdel Rahman A, Ryczko M, Drucker DJ, Dennis JW (2014). N-glycan remodeling on glucagon receptor is an effector of nutrient sensing by the hexosamine biosynthesis pathway. J Biol Chem.

[32] Khidekel N, Arndt S, Lamarre-Vincent N, Lippert A, Poulin-Kerstien KG, Ramakrishnan B, Qasba PK, Hsieh-Wilson LC (2003). A chemoenzymatic approach toward the rapid and sensitive detection of O-GlcNAc posttranslational modifications. J Am Chem Soc.

[33] Kim S, Maynard JC, Sasaki Y, Strickland A, Sherman DL, Brophy PJ, Burlingame AL, Milbrandt J (2016). Schwann cell O-GlcNAc glycosylation is required for myelin maintenance and axon integrity. J Neurosci.

[34] Kroner A, Greenhalgh AD, Zarruk JG, Passos Dos Santos R, Gaestel M, David S (2014). TNF and increased intracellular iron alter macrophage polarization to a detrimental M1 phenotype in the injured spinal cord. Neuron.

[35] Kunnumakkara AB, Shabnam B, Girisa S, Harsha C, Banik K, Devi TB, Choudhury R, Sahu H, Parama D, Sailo BL (2020). Inflammation, NF-kappaB, and chronic diseases: how are they linked?. Crit Rev Immunol.

[36] Lagerlof O, Hart GW, Huganir RL (2017). O-GlcNAc transferase regulates excitatory synapse maturity. Proc Natl Acad Sci USA.

[37] Lagerlof O, Slocomb JE, Hong I, Aponte Y, Blackshaw S, Hart GW, Huganir RL (2016). The nutrient sensor OGT in PVN neurons regulates feeding. Science.

[38] Lawrence T (2009). The nuclear factor NF-kappaB pathway in inflammation. Cold Spring Harb Perspect Biol.

[39] Lee JH, Kim JY, Noh S, Lee H, Lee SY, Mun JY, Park H, Chung WS (2020). Astrocytes phagocytose adult hippocampal synapses for circuit homeostasis. Nature.

[40] Levine ZG, Walker S (2016). The biochemistry of O-GlcNAc transferase: which functions make it essential in mammalian cells?. Annu Rev Biochem.

[41] Li D, Liu N, Zhao HH, Zhang X, Kawano H, Liu L, Zhao L, Li HP (2017). Interactions between Sirt1 and MAPKs regulate astrocyte activation induced by brain injury in vitro and in vivo. J Neuroinflamm.

[42] Li L, Zang L, Zhang F, Chen J, Shen H, Shu L, Liang F, Feng C, Chen D, Tao H (2017). Fat mass and obesity-associated (FTO) protein regulates adult neurogenesis. Hum Mol Genet.

[43] Li X, Zhu Q, Shi X, Cheng Y, Li X, Xu H, Duan X, Hsieh-Wilson LC, Chu J, Pelletier J (2019). O-GlcNAcylation of core components of the translation initiation machinery regulates protein synthesis. Proc Natl Acad Sci USA.

[44] Liddelow SA, Barres BA (2017). Reactive astrocytes: production, function, and therapeutic potential. Immunity.

[45] Liddelow SA, Guttenplan KA, Clarke LE, Bennett FC, Bohlen CJ, Schirmer L, Bennett ML, Munch AE, Chung WS, Peterson TC (2017). Neurotoxic reactive astrocytes are induced by activated microglia. Nature.

[46] Linnerbauer M, Wheeler MA, Quintana FJ (2020). Astrocyte crosstalk in CNS inflammation. Neuron.

[47] Liu R, Chen Y, Liu G, Li C, Song Y, Cao Z, Li W, Hu J, Lu C, Liu Y (2020). PI3K/AKT pathway as a key link modulates the multidrug resistance of cancers. Cell Death Dis.

[48] Ma X, Li H, He Y, Hao J (2017). The emerging link between O-GlcNAcylation and neurological disorders. Cell Mol Life Sci.

[49] McConnell MJ, Moran JV, Abyzov A, Akbarian S, Bae T, Cortes-Ciriano I, Erwin JA, Fasching L, Flasch DA, Freed D (2017). Intersection of diverse neuronal genomes and neuropsychiatric disease: the brain somatic mosaicism network. Science.

[50] Mota M, Porrini V, Parrella E, Benarese M, Bellucci A, Rhein S, Schwaninger M, Pizzi M (2020). Neuroprotective epi-drugs quench the inflammatory response and microglial/macrophage activation in a mouse model of permanent brain ischemia. J Neuroinflamm.

[51] Neal M, Richardson JR (2018). Epigenetic regulation of astrocyte function in neuroinflammation and neurodegeneration. Biochim Biophys Acta Mol Basis Dis.

[52] Park J, Ha HJ, Chung ES, Baek SH, Cho Y, Kim HK, Han J, Sul JH, Lee J, Kim E (2021). O-GlcNAcylation ameliorates the pathological manifestations of Alzheimer’s disease by inhibiting necroptosis. Sci Adv.

[53] Park J, Lai MK, Arumugam TV, Jo D-G (2020). O-GlcNAcylation as a therapeutic target for Alzheimer’s disease. Neuromol Med.

[54] Richetin K, Steullet P, Pachoud M, Perbet R, Parietti E, Maheswaran M, Eddarkaoui S, Begard S, Pythoud C, Rey M (2020). Tau accumulation in astrocytes of the dentate gyrus induces neuronal dysfunction and memory deficits in Alzheimer’s disease. Nat Neurosci.

[55] Ruan HB, Dietrich MO, Liu ZW, Zimmer MR, Li MD, Singh JP, Zhang K, Yin R, Wu J, Horvath TL (2014). O-GlcNAc transferase enables AgRP neurons to suppress browning of white fat. Cell.

[56] Ryan P, Xu M, Davey AK, Danon JJ, Mellick GD, Kassiou M, Rudrawar S (2019). O-GlcNAc modification protects against protein misfolding and aggregation in neurodegenerative disease. ACS Chem Neurosci.

[57] Ryczko MC, Pawling J, Chen R, Abdel Rahman AM, Yau K, Copeland JK, Zhang C, Surendra A, Guttman DS, Figeys D (2016). Metabolic reprogramming by hexosamine biosynthetic and Golgi N-glycan branching pathways. Sci Rep.

[58] Santello M, Toni N, Volterra A (2019). Astrocyte function from information processing to cognition and cognitive impairment. Nat Neurosci.

[59] Shih RH, Wang CY, Yang CM (2015). NF-kappaB signaling pathways in neurological inflammation: a mini review. Front Mol Neurosci.

[60] Sofroniew MV (2014). Astrogliosis. Cold Spring Harb Perspect Biol.

[61] Staszewski O, Prinz M (2014). Glial epigenetics in neuroinflammation and neurodegeneration. Cell Tissue Res.

[62] Su C, Schwarz TL (2017). O-GlcNAc transferase is essential for sensory neuron survival and maintenance. J Neurosci.

[63] Sun X, Hu X, Wang D, Yuan Y, Qin S, Tan Z, Gu Y, Huang X, He C, Su Z (2017). Establishment and characterization of primary astrocyte culture from adult mouse brain. Brain Res Bull.

[64] Torres CR, Hart GW (1984). Topography and polypeptide distribution of terminal *N*-acetylglucosamine residues on the surfaces of intact lymphocytes. Evidence for O-linked GlcNAc. J Biol Chem.

[65] Viatour P, Merville MP, Bours V, Chariot A (2005). Phosphorylation of NF-kappaB and IkappaB proteins: implications in cancer and inflammation. Trends Biochem Sci.

[66] Wall PM, Messier C (2002). Infralimbic kappa opioid and muscarinic M1 receptor interactions in the concurrent modulation of anxiety and memory. Psychopharmacology.

[67] Wang AC, Jensen EH, Rexach JE, Vinters HV, Hsieh-Wilson LC (2016). Loss of O-GlcNAc glycosylation in forebrain excitatory neurons induces neurodegeneration. Proc Natl Acad Sci USA.

[68] Wang X, Li X, Zuo X, Liang Z, Ding T, Li K, Ma Y, Li P, Zhu Z, Ju C (2021). Photobiomodulation inhibits the activation of neurotoxic microglia and astrocytes by inhibiting Lcn2/JAK2-STAT3 crosstalk after spinal cord injury in male rats. J Neuroinflamm.

[69] Wani WY, Chatham JC, Darley-Usmar V, McMahon LL, Zhang J (2017). O-GlcNAcylation and neurodegeneration. Brain Res Bull.

[70] Wheatley EG, Albarran E, White CW, Bieri G, Sanchez-Diaz C, Pratt K, Snethlage CE, Ding JB, Villeda SA (2019). Neuronal O-GlcNAcylation improves cognitive function in the aged mouse brain. Curr Biol.

[71] Wheeler MA, Jaronen M, Covacu R, Zandee SEJ, Scalisi G, Rothhammer V, Tjon EC, Chao CC, Kenison JE, Blain M (2019). Environmental control of astrocyte pathogenic activities in CNS inflammation. Cell.

[72] White CW, Fan X, Maynard JC, Wheatley EG, Bieri G, Couthouis J, Burlingame AL, Villeda SA (2020). Age-related loss of neural stem cell O-GlcNAc promotes a glial fate switch through STAT3 activation. Proc Natl Acad Sci USA.

[73] Xie S, Jin N, Gu J, Shi J, Sun J, Chu D, Zhang L, Dai CL, Gu JH, Gong CX (2016). O-GlcNAcylation of protein kinase A catalytic subunits enhances its activity: a mechanism linked to learning and memory deficits in Alzheimer’s disease. Aging Cell.

[74] Yang WH, Park SY, Nam HW, Kim DH, Kang JG, Kang ES, Kim YS, Lee HC, Kim KS, Cho JW (2008). NFkappaB activation is associated with its O-GlcNAcylation state under hyperglycemic conditions. Proc Natl Acad Sci USA.

[75] Yang X, Qian K (2017). Protein O-GlcNAcylation: emerging mechanisms and functions. Nat Rev Mol Cell Biol.

[76] Yuzwa SA, Shan X, Macauley MS, Clark T, Skorobogatko Y, Vosseller K, Vocadlo DJ (2012). Increasing O-GlcNAc slows neurodegeneration and stabilizes tau against aggregation. Nat Chem Biol.

[77] Yuzwa SA, Vocadlo DJ (2014). O-GlcNAc and neurodegeneration: biochemical mechanisms and potential roles in Alzheimer’s disease and beyond. Chem Soc Rev.

[78] Zhang C, Atasoy D, Araç D, Yang X, Fucillo MV, Robison AJ, Ko J, Brunger AT, Südhof TC (2010). Neurexins physically and functionally interact with GABA(A) receptors. Neuron.

[79] Zhang Q, Lenardo MJ, Baltimore D (2017). 30 Years of NF-kappaB: a blossoming of relevance to human pathobiology. Cell.

[80] Zhang Z, Tan EP, VandenHull NJ, Peterson KR, Slawson C (2014). O-GlcNAcase expression is sensitive to changes in O-GlcNAc homeostasis. Front Endocrinol.

[81] Zhu Q, Cheng X, Cheng Y, Chen J, Xu H, Gao Y, Duan X, Ji J, Li X, Yi W (2020). O-GlcNAcylation regulates the methionine cycle to promote pluripotency of stem cells. Proc Natl Acad Sci USA.

[82] Zhu Y, Wang Y, Yao R, Hao T, Cao J, Huang H, Wang L, Wu Y (2017). Enhanced neuroinflammation mediated by DNA methylation of the glucocorticoid receptor triggers cognitive dysfunction after sevoflurane anesthesia in adult rats subjected to maternal separation during the neonatal period. J Neuroinflamm.

