# Supplementary figures and images for: Ogt-mediated O-GlcNAcylation inhibits astrocytes activation through modulating NF-κB signaling pathway
Source: J Neuroinflammation. 2023 Jun 22;20:146. doi: 10.1186/s12974-023-02824-8 (PMC10286367; doi:10.1186/s12974-023-02824-8)

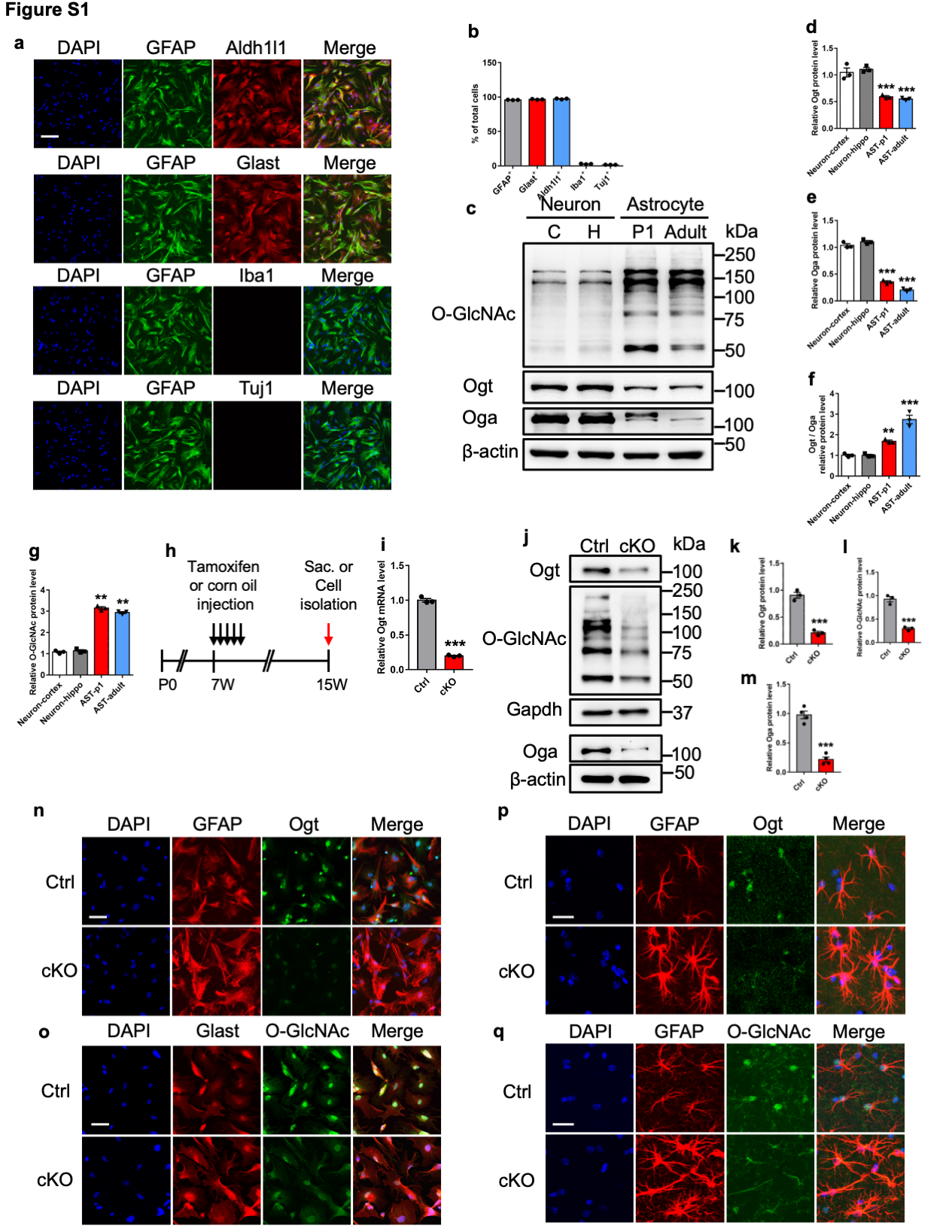

Supplement: Supplementary file 1 — Additional file 1: Figure S1. Ogt deficient astrocytes showed the reduced levels of Ogt and O-GlcNAcylation.Representative images of GFAP, Aldh1l1, Glast, Iba1, and Tuj1 immunostaining with cultured adult Ctrl astrocytes. Scale bar, 50 μm.Quantification results show that the percentage of GFAP+, Glast+ and Aldh1l1+ cells are all around 97%, the percentage of Tuj1+ cells is around 2%, and the percentage of Iba1+ cells is around 3%. n = 3 independent experiments. Values represent mean ± SEM.WB assayand quantification results showed that P1 and adult astrocytes showed the decreased levels of Ogtand Oga, but the increased Ogt/Ogaand O-GlcNAcylation levelcompared with cortical and hippocampal neurons. n = 3 independent experiments for each group. Values represent mean ± SEM; *p < 0.05, **p < 0.01, ***p < 0.001; one-way ANOVA analysis followed by Tukey’s multiple-comparison test, F = 38.5 for, F = 333.8 for, F = 51.81 for, F = 418.4 for. C: cortical neurons. H, hippocampal neurons; P1, postnatal day 1; Adult, postnatal 7-week.Schematic illustration of tamoxifen administration strategy. AdultGlast-CreERT2::Ogtfloxp/Y mice were intraperitoneallyadministrated with tamoxifenand corn oil, respectively. Eight weeks after tamoxifen administration, mice were sacrificed for assays.qRT-PCR results showed that mRNA level of Ogt significantly decreased in adult cKO astrocytes compared with Ctrl astrocytes. n = 3 independent experiments. Values represent mean ± SEM; *p < 0.05, **p < 0.01, ***p < 0.001; unpaired Student’s t-test.WB assayand quantification results showed that the protein levels of Ogtand O-GlcNAcylationsignificantly decreased in adult cKO astrocytes compared with Ctrl astrocytes. The level of Oga was also decreased in cKO cells. n = 3 independent experiments. Values represent mean ± SEM; *p < 0.05, **p < 0.01, ***p < 0.001; unpaired Student’s t-test.Representative images of GFAP-Ogtand Glast-O-GlcNAcylationimmunostaining with cultured adult Ctrl and cKO astrocytes. Scal [file 12974_2023_2824_MOESM1_ESM.tiff]

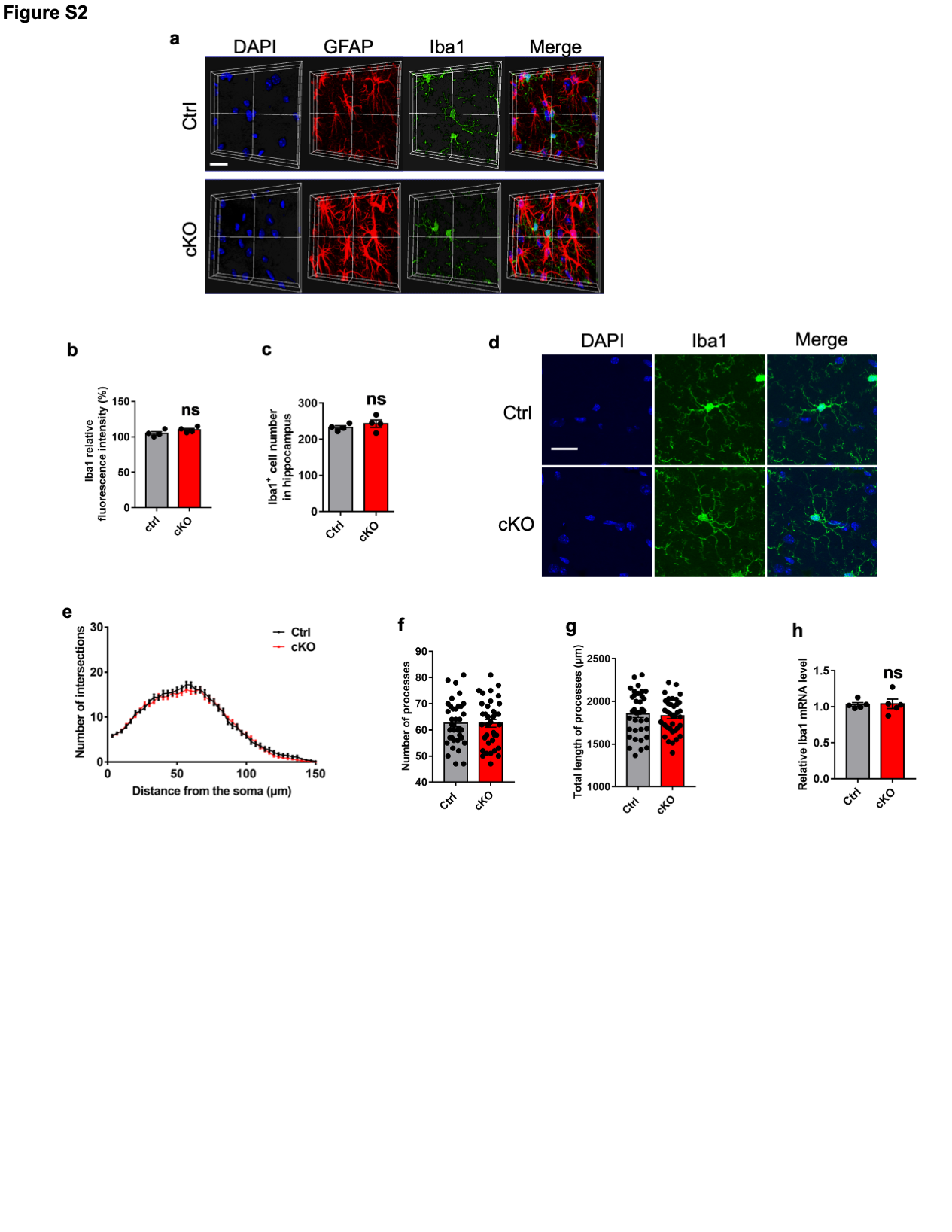

Supplement: Supplementary file 2 — Additional file 2: Figure S2. Astrocytic Ogt deficiency does not affect microglia, and Ogt cKO in neural stem/progenitor cells does not induce inflammation in vivo.Three-dimensionalview of astrocytesand microgliaimmunostaining in the hippocampal regions of Ctrl and cKO mice. Scale bar, 20 μm.Quantification results showed that the level of Iba1 fluorescence intensityand the number of Iba1+ cellsshowed no difference in the hippocampus region between Ctrl and cKO mice. n = 4 mice per genotype. Values represent mean ± SEM; *p < 0.05, **p < 0.01, ***p < 0.001; unpaired Student’s t-test.Representative images of Iba1 immunostaining in the hippocampal regions of Ctrl and cKO mice. Scale bar, 20 μm.Sholl analysis showed no significant difference in the number of neurite intersections per radius, the number of neurites per cell, and the total length of neuritesof microglia in the hippocampal regions of Ogt cKO mice compared with Ctrl mice. 12 cells were picked up per animal and total 36 cells from 3 mice were analyzed per group; Values represent mean ± SEM; *p < 0.05, **p < 0.01, ***p < 0.001; two-way ANOVA analysis followed by Sidak's multiple-comparison test for, F = 5.062; unpaired Student’s t-test for.qRT-PCR results showed that mRNA level of Iba1 in the hippocampus showed no difference between Ctrl and cKO mice. n = 5 mice per genotype. Values represent mean ± SEM; *p < 0.05, **p < 0.01, ***p < 0.001; unpaired Student’s t-test. [file 12974_2023_2824_MOESM2_ESM.tiff]

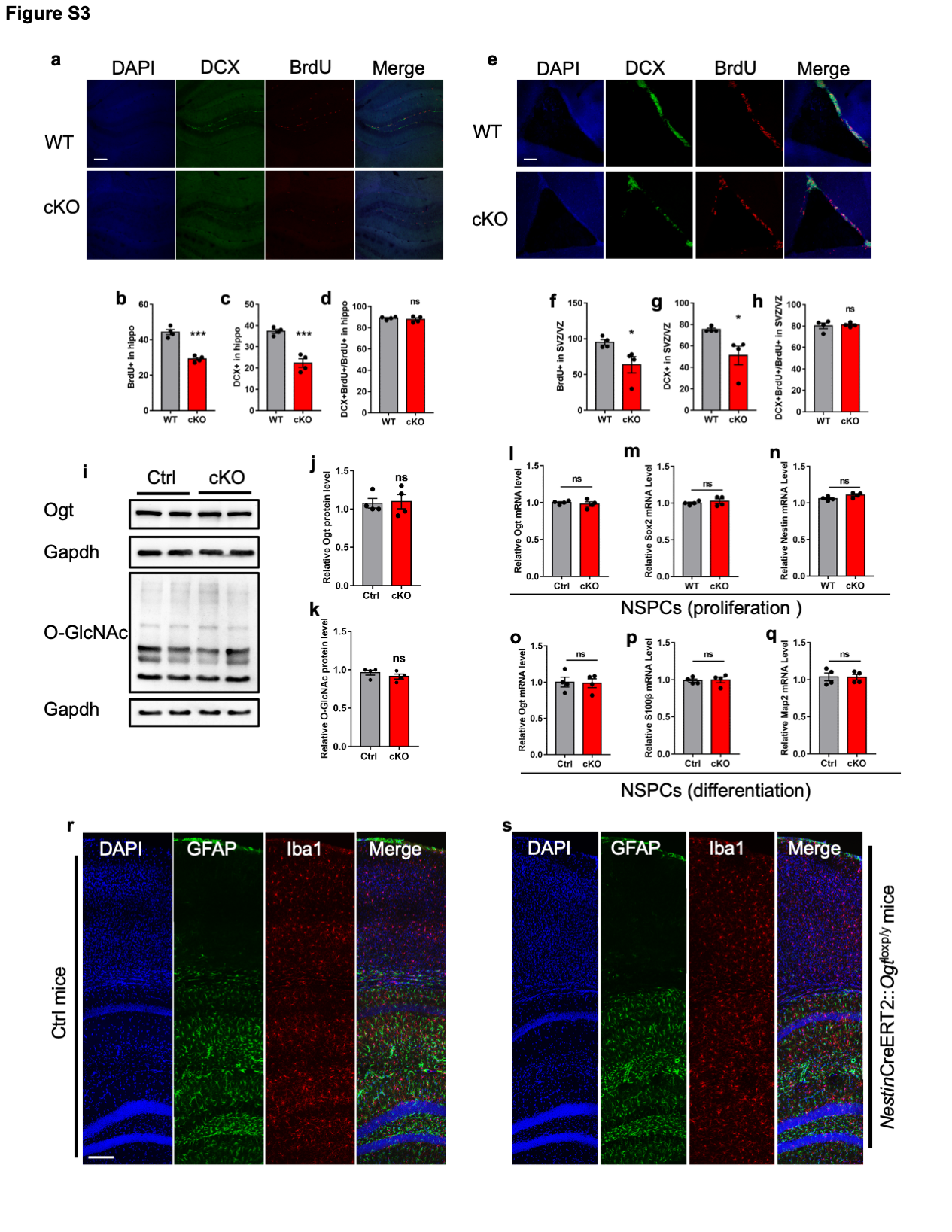

Supplement: Supplementary file 3 — Additional file 3: Figure S3. Astrocytic Ogt deficiency impairs adult neurogenesis, and Ogt cKO in neural stem/progenitor cells does not induce inflammation in vivo.Representative images of DCX and BrdU immunostaining in the subgranular zoneof the hippocampus of Ctrl and Ogt cKO mice. Scale bar, 100 μm.Quantification results showed that cKO mice had the reduced number of BrdU+ cellsand DCX+cells, but a similar percentage of BrdU+DCX+/BrdU+in the SGZ of Ctrl and Ogt cKO mice. mice were injectedwith BrdU and sacrificed 1-day post the final BrdU administration. n = 4 mice per genotype. Values represent mean ± SEM; *p < 0.05, **p < 0.01, ***p < 0.001; unpaired Student’s t-test.Representative images of DCX and BrdU immunostaining in the subventricular zoneof the lateral ventricles of Ctrl and Ogt cKO mice. Scale bar, 100 μm.Quantification results showed that cKO mice had the reduced number of of BrdU+ cellsand DCX+cells, but a similar percentage of BrdU+DCX+/BrdU+in the SVZ of Ctrl and Ogt cKO mice. mice were injectedwith BrdU and sacrificed 1-day post the final BrdU administration. n = 4 mice per genotype. Values represent mean ± SEM; *p < 0.05, **p < 0.01, ***p < 0.001; unpaired Student’s t-test.WB assayand quantification resultsshowed that the protein level of Ogt and O-GlcNAcylation were not affected in proliferating adult neural stem/progenitor cellsof Ctrl and Ogt cKO mice. n = 4 independent experiments. Values represent mean ± SEM; *p < 0.05, **p < 0.01, ***p < 0.001; unpaired Student’s t-test.qRT-PCR results showed that under proliferating condition, Ctrl and cKO aNSPCs showed no difference in mRNA levels of Ogt, Sox2and Nestin. n = 4 independent experiments. Values represent mean ± SEM; *p < 0.05, **p < 0.01, ***p < 0.001; unpaired Student’s t-test.qRT-PCR results showed that under differentiation condition, Ctrl and cKO aNSPCs showed no difference in mRNA levels of mRNA level of Ogt, S100βand Map2. n = 4 independent experiments. Values represent mean ± SEM; *p [file 12974_2023_2824_MOESM3_ESM.tiff]

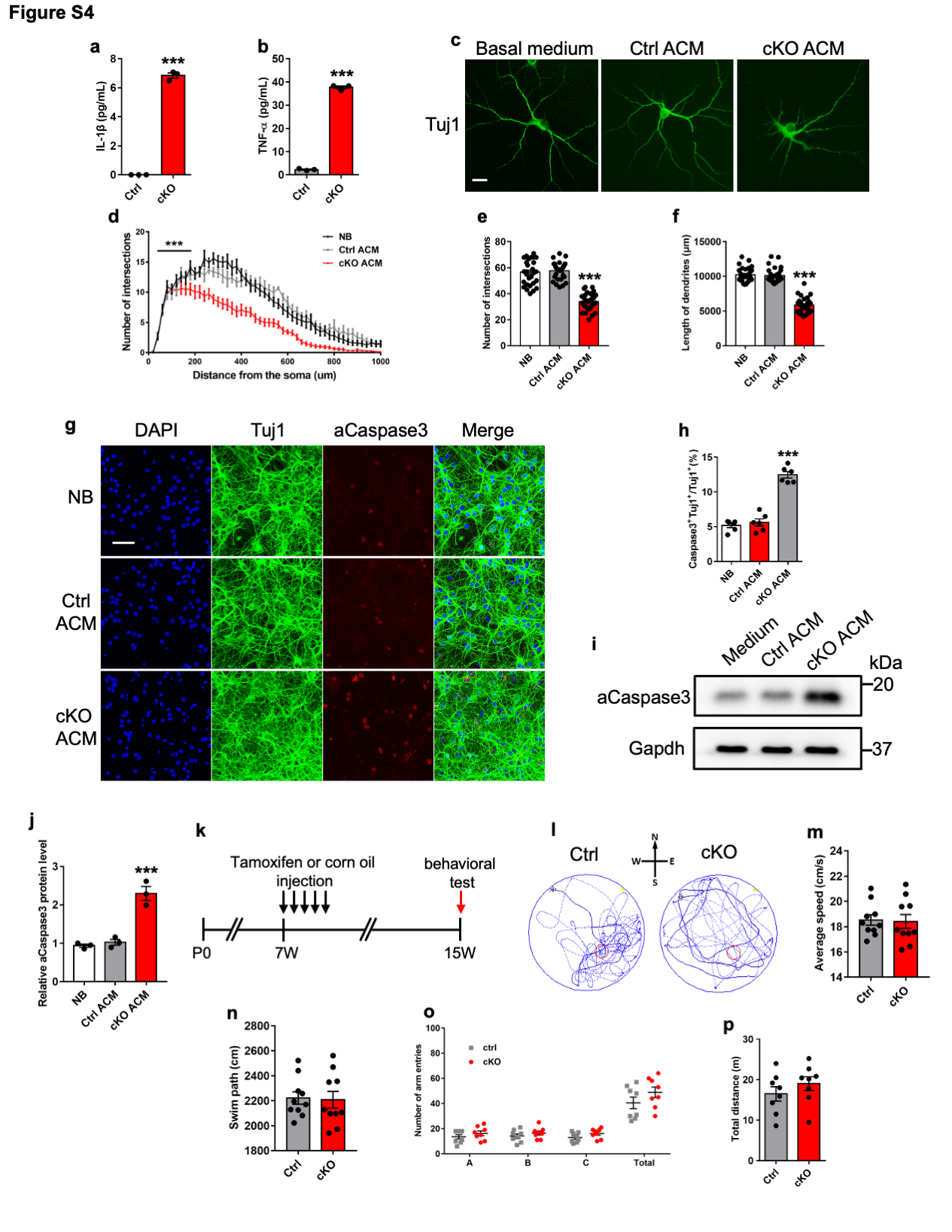

Supplement: Supplementary file 4 — Additional file 4: Figure S4. Reactive astrocytes impair hippocampal neuronal cells and cognition of mice.ELISA results showed that the levels of IL-1βand TNF-αremarkably increased in the supernatants of cKO astrocyte-conditioned medium. n = 3 independent experiments. Values represent mean ± SEM; *p < 0.05, **p < 0.01, ***p < 0.001; unpaired Student’s t-test.Representative images of Tuj1 immunostaining with hippocampal neurons at day in vitro 10cultured with neurobasal medium, Ctrl and cKO astrocyte-conditioned medium, respectively. Scale bar, 20 μm.Sholl analysis showed that hippocampal neurons cultured with cKO ACM displayed the overall decrease in the number of dendritic intersections per radius, the number of dendrites per celland total length of dendritesat DIV 10 compared with NB- and Ctrl ACM-cultured cells. Values represent mean ± SEM; *p < 0.05, **p < 0.01, ***p < 0.001; n = 36 neurons from 3 independent experiments for each group; two-way ANOVA analysis followed by Sidak’s multiple-comparison test for, F = 353.5; one-way ANOVA analysis followed by Tukey’s multiple-comparison test for, F = 87.52 for, F = 168.7 for.Representative images of Tuj1 and activated-Caspase3immunostaining with hippocampal neuronscultured with neurobasal medium, Ctrl and cKO ACM, respectively. Scale bar, 50 μm.Quantification results showed that hippocampal neurons cultured with cKO ACM displayed the increased percentage of activated-Caspase3+ cells compared to that with NB- and Ctrl ACM-cultured cells. n = 6 independent experiments. Values represent mean ± SEM; *p < 0.05, **p < 0.01, ***p < 0.001; one-way ANOVA analysis followed by Tukey’s multiple-comparison test, F = 86.5.WB assayand quantification resultsshowed that the protein level of aCaspase3 significantly increased in hippocampal neurons cultured with cKO ACM compared to cell cultured with NB and Ctrl ACM, respectively. n = 3 independent experiments. Values represent mean ± SEM; *p < 0.05, **p < 0.01, ***p < 0.001; one-way AN [file 12974_2023_2824_MOESM4_ESM.tiff]

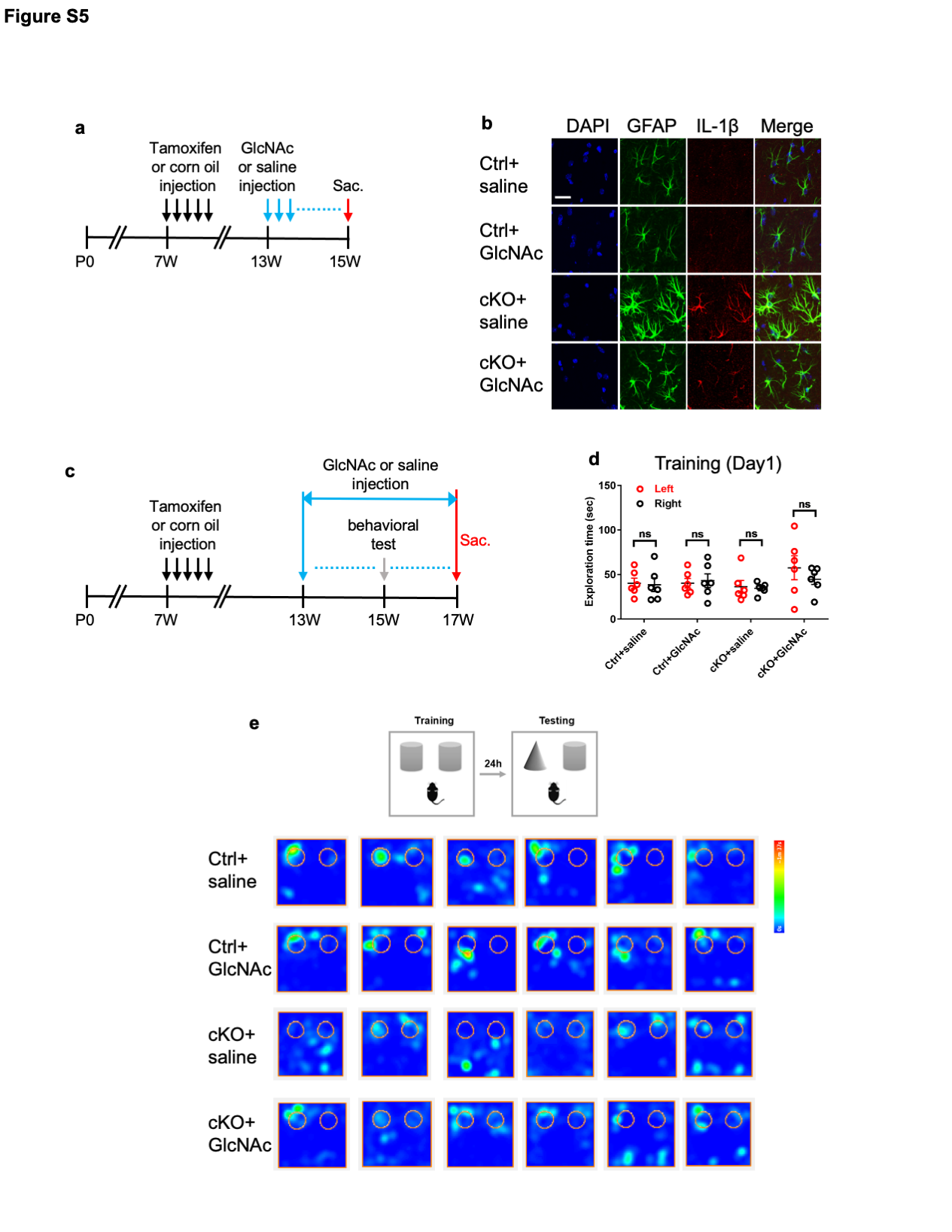

Supplement: Supplementary file 5 — Additional file 5: Figure S5. Restoration of O-GlcNAcylation inhibits astrocyte activation and inflammation, and improves cognitive function of Ogt deficient mice.Schematic illustration of tamoxifen and GlcNAc administration strategy. AdultGlast-CreERT2::Ogtfloxp/Y mice were intraperitoneally injected with tamoxifenand corn oil, respectively. 6 weeks after tamoxifen administration, Ctrl and cKO mice were injected with salineand GlcNAcfor 14 days, and then the mice were sacrificed for assays.Representative images of GFAP and IL-1β immunostaining with brain sections of Ctrl and cKO mice treated with salineand GlcNAc, respectively. Scale bar, 20 μm.Schematic illustration of tamoxifen, GlcNAc administration and behavioral test strategy. AdultGlast-CreERT2::Ogtfloxp/Y mice were intraperitoneally injected with tamoxifenand corn oilfor, respectively. Six weeks after tamoxifen administration, Ctrl and cKO mice were injected with salineor GlcNAcfor 14 days, respectively, and behavioral tests were performed. Saline and GlcNAc was continuously injected during the behavioral tests.Mice from the four group showed no difference in the percentage of time spent exploring two identical objectsand during the training period in novel object recognition task test. n = 6 mice per group; Values represent mean ± SEM; *p < 0.05, **p < 0.01, ***p < 0.001; two-way ANOVA analysis followed by Sidak's multiple-comparison test, F = 0.3952.Schematic illustration of novel object recognition task and heatmap image of each animal showing the distribution of exploring time of 4 groups mice during the testing trial. n = 6 mice per group. [file 12974_2023_2824_MOESM5_ESM.tiff]

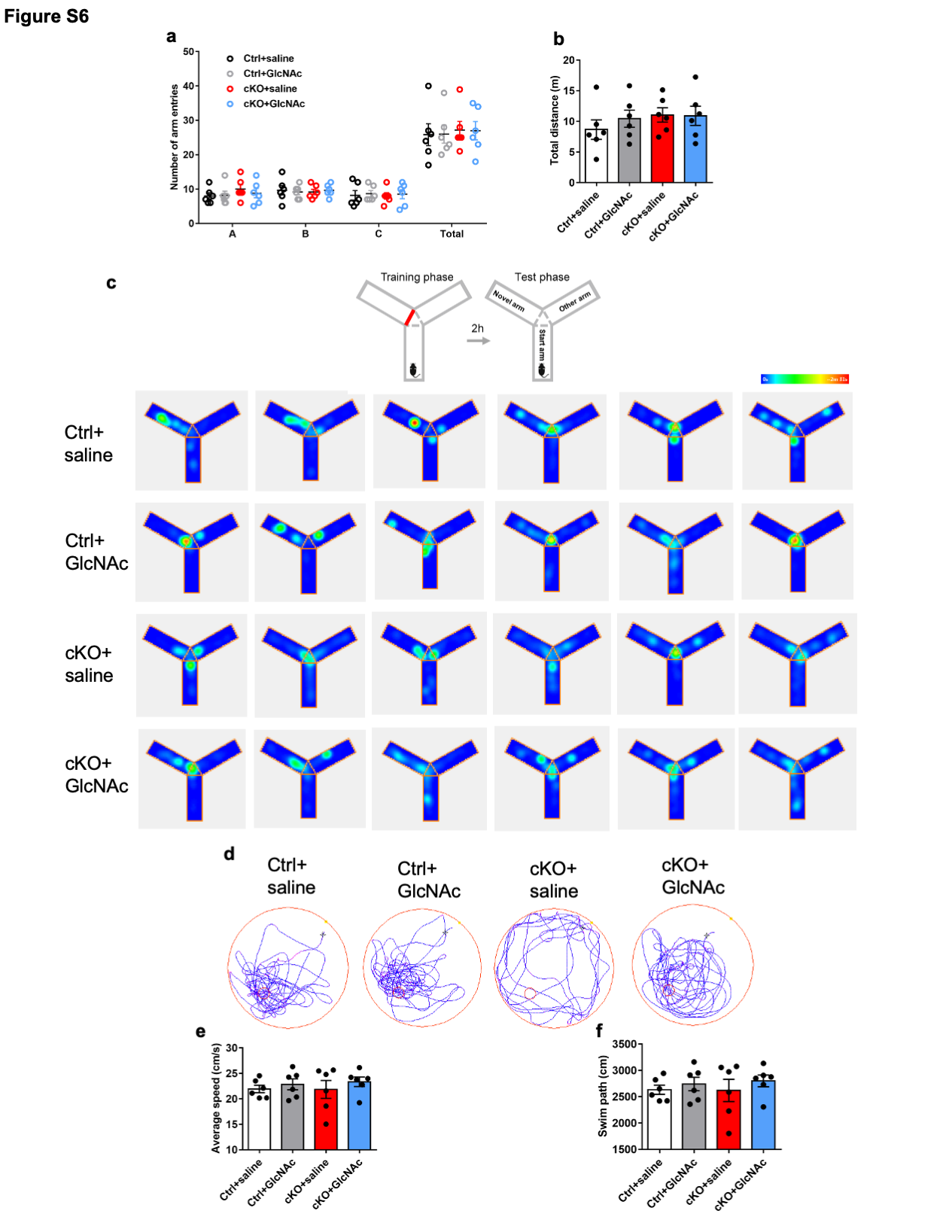

Supplement: Supplementary file 6 — Additional file 6: Figure S6. Restoration of O-GlcNAcylation improves cognitive function.Mice showed no difference in the number of arm entries, total distancein Y maze spontaneous alternation test between four groups. n = 6 mice per group. Values represent mean ± SEM; *p < 0.05, **p < 0.01, ***p < 0.001; two-way ANOVA analysis followed by Sidak's multiple-comparison test for, F = 0.1623; one-way ANOVA analysis followed by Tukey’s multiple-comparison test for, F = 0.5806.Schematic Illustration of Y spontaneous alternation test and representative heat map images of mice during Y maze spontaneous alternation test.Representative images of the swimming path during the probe trial of Morris Water Maze test. The red circle represents the platform.The average swimming speedand total swimming path lengthshowed no difference in all four groups during the probe trial test. n = 6 mice per group; Values represent mean ± SEM; *p < 0.05, **p < 0.01, ***p < 0.001; one-way ANOVA analysis followed by Tukey’s multiple-comparison test, F = 0.3744 forand F = 0.3702 for. [file 12974_2023_2824_MOESM6_ESM.tiff]

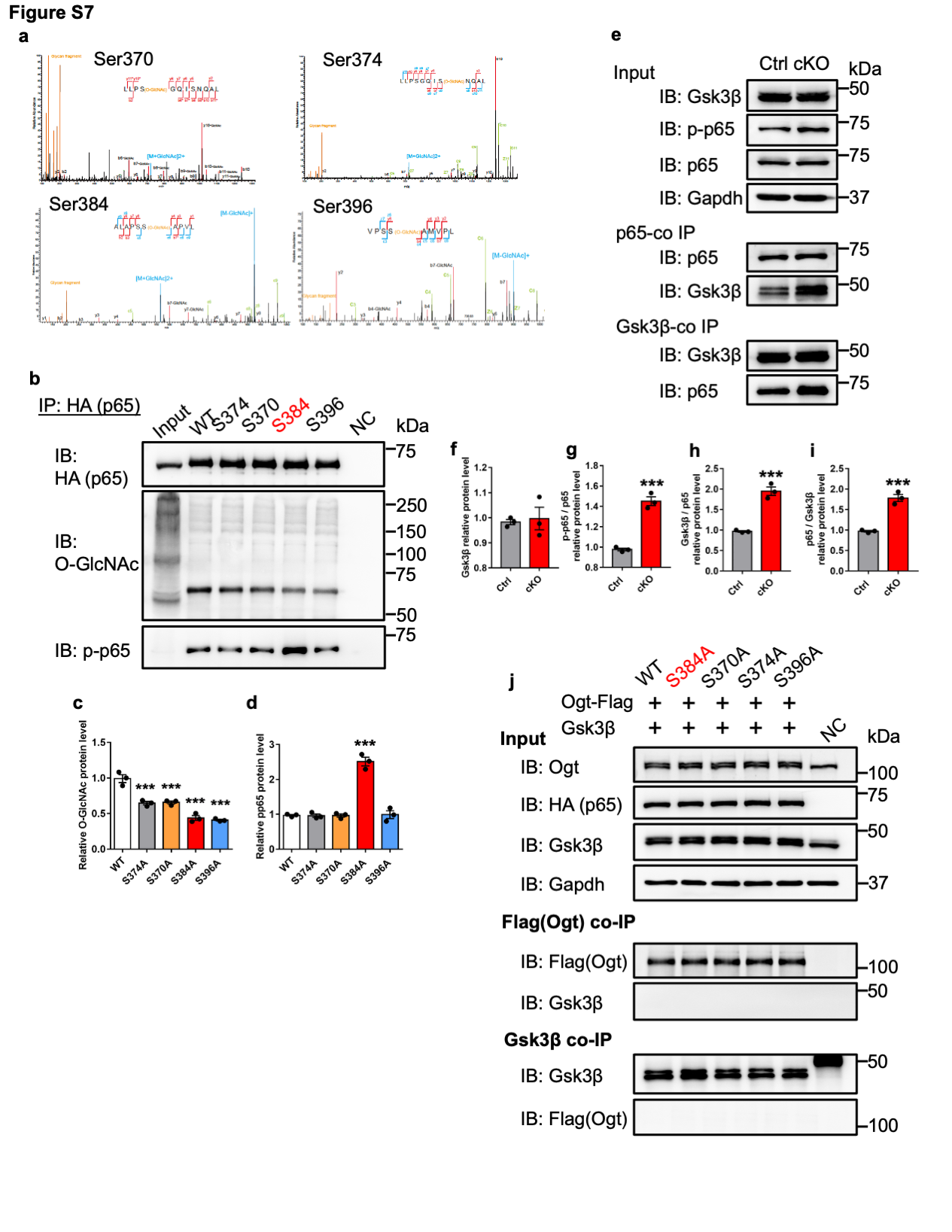

Supplement: Supplementary file 7 — Additional file 7: Figure S7. Ogt interacts with NF-κB and catalyzes the O-GlcNAcylation of NF-κB.Schematic illustration of O-GlcNAcylation sites of p65 on S374, S370, S384 and S396 in N2a cells identified by MS/MS analysis. nanoLC-LTQ-CID was used to mapping the sites of O-GlcNAcylation on p65. The matched fragment ions are labeled in y and b.IP-WB assayand quantification results show that mutation of single potential O-GlcNAcylation sites led to a significant decrease in the O-GlcNAcylation level of p65in N2a cells, and only the mutation of S384 induced a significant increase of p-p65. n = 3 independent experiments. Values represent mean ± SEM; *p < 0.05, **p < 0.01, ***p < 0.001; one-way ANOVA analysis followed by Tukey’s multiple-comparison test, F = 51.62 forand F = 74.06 for.IP followed by WB assay results showed the level of GSK3β showed no difference in the hippocampus of Ctrl and cKO mice, and the interaction between p65 and GSK3β was significantly increased in the hippocampus of Ctrl and cKO mice. n = 3 independent experiments. Values represent mean ± SEM; *p < 0.05, **p < 0.01, ***p < 0.001; unpaired Student’s t-test.co-IP followed by WB assay showed no direct interaction between Gsk3β and Ogt. [file 12974_2023_2824_MOESM7_ESM.tiff]

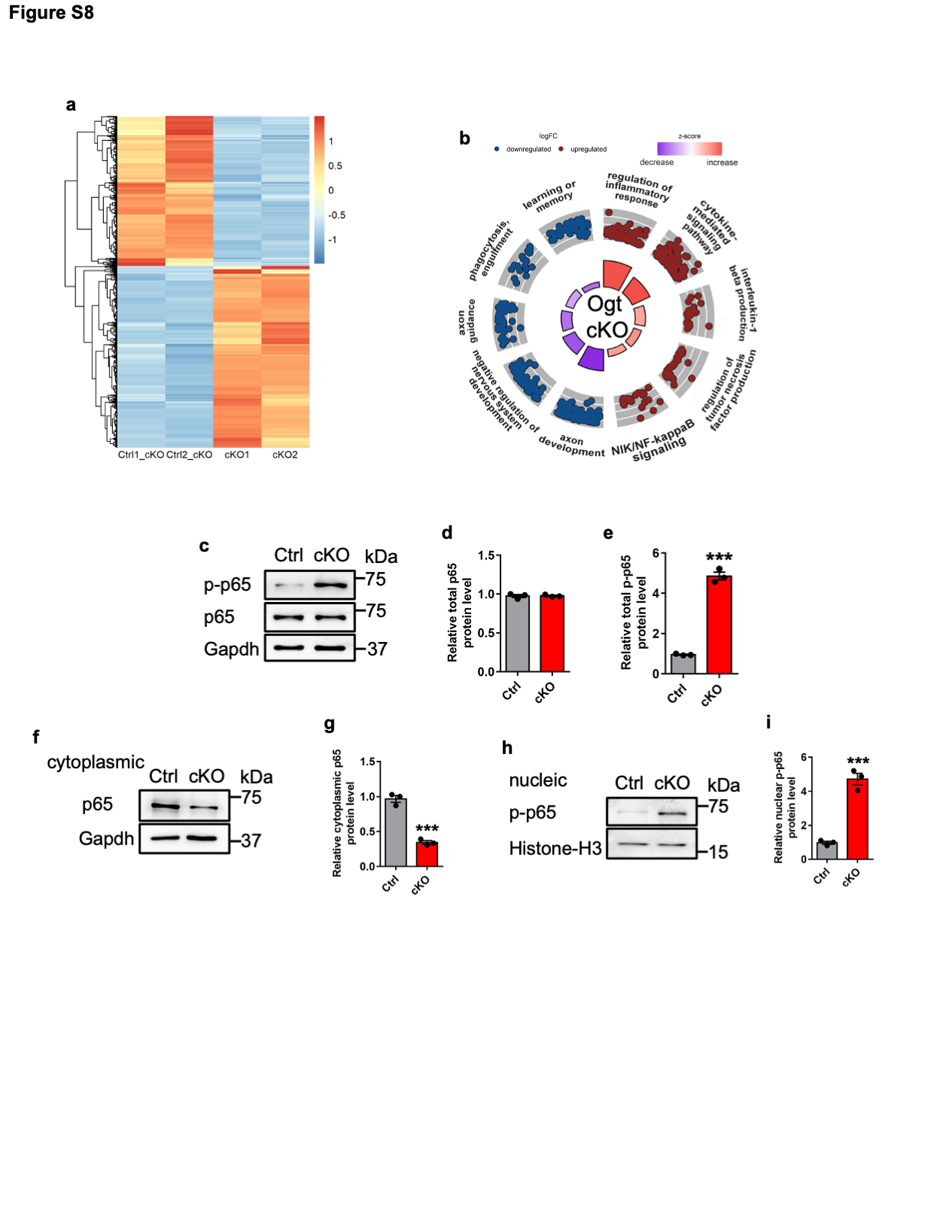

Supplement: Supplementary file 8 — Additional file 8: Figure S8. RNA-seq data analysis shows that Ogt deficiency activates NF-κB signaling pathway in astrocytes.Heatmap illustrating the altered transcriptome of cKO astrocytes group compared to Ctrl astrocytes. Red color, up-regulated genes; blue color, down-regulated genes. Two biological replicate samples were used for sequencing in each group.Gene ontologyCircle visualization of differentially expressed genesenriched biological process terms in cKO astrocytes including inflammatory response, cytokine signaling, NF-κB signaling, axon development and learning and memory. The outer circle showed the log2 fold changeof the genes in each category, and the height of the inner bar plot indicated the significance level of the GO term), and the color represents the Z score.WB assayand quantification results showed that Ogt deficiency did not affect the level of total p65, but significantly increased the protein level of p-p65in cultured astrocytes. n = 3 independent experiments. Values represent mean ± SEM; *p < 0.05, **p < 0.01, ***p < 0.001; unpaired Student’s t-test.WB assaysand quantification results showed that Ogt deficiency significantly reduced the level of p65 in cytoplasmand significantly increased the level of p-p65 in nucleus. n = 3 independent experiments. Values represent mean ± SEM; *p < 0.05, **p < 0.01, ***p < 0.001; unpaired Student’s t-test. [file 12974_2023_2824_MOESM8_ESM.tiff]

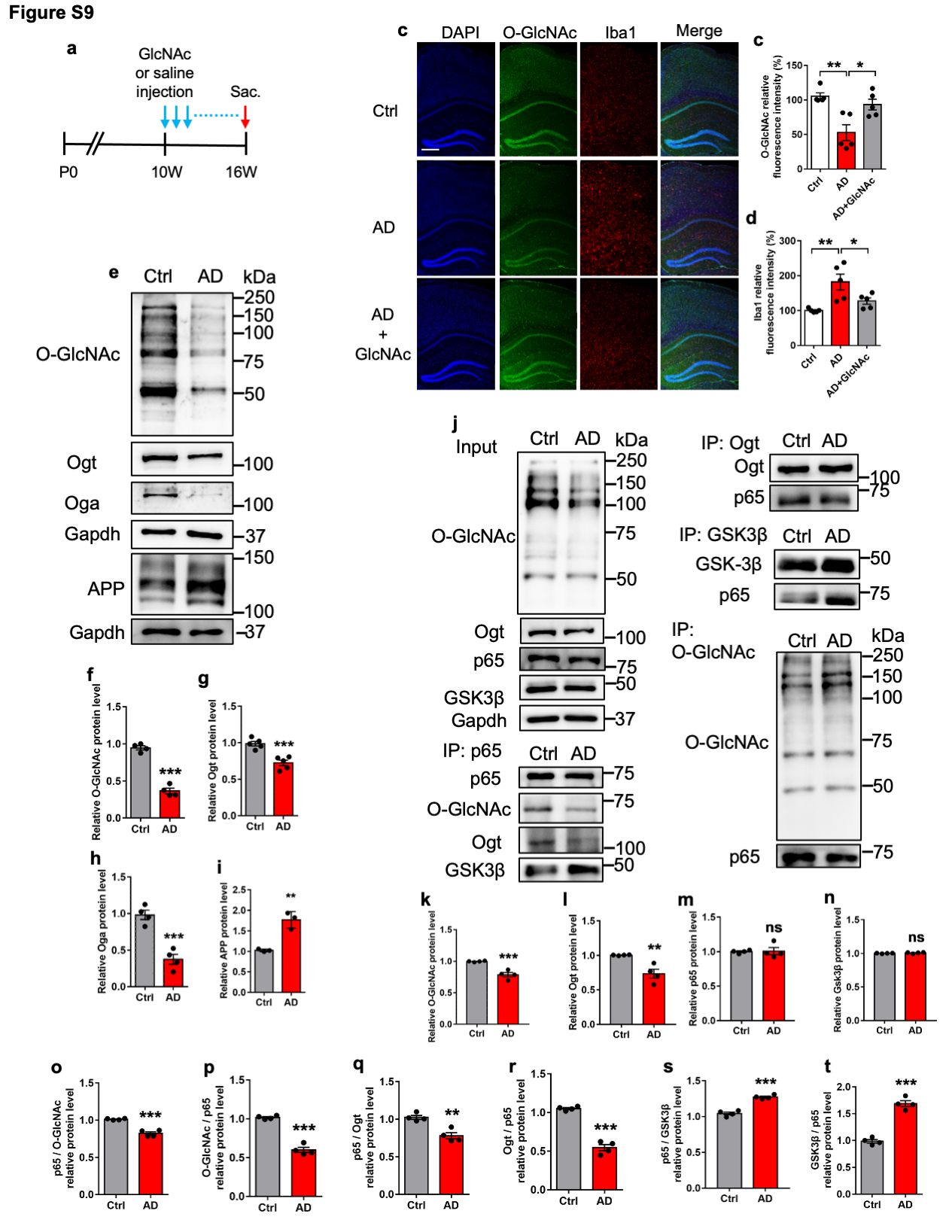

Supplement: Supplementary file 9 — Additional file 9: Figure S9. Restoration of O-GlcNAcylation inhibits astrocyte activation and inflammation, and improves cognitive function of Ogt deficient mice.Schematic illustration of GlcNAc administration strategy. 2.5-month-old Ctrl and AD mice were administrated with salineand GlcNAcfor 6 weeks, and mice were sacrificed for assays.Representative images of O-GlcNAcylation and Iba1 immunostaining with brain sections of Ctrl and cKO mice treated with salineand GlcNAc, respectively. Scale bar, 100 μm.Quantification results show that the level of of O-GlcNAcylation fluorescence intensitysignificantly decreased in the hippocampus region of AD mice compared to Ctrl mice, which was significantly increased by GlcNAc administration. The intensity of Iba1 fluorescence was also significantly reduced after GlcNAc administration. n = 5 mice per group. Values represent mean ± SEM; *p < 0.05, **p < 0.01, ***p < 0.001; one-way ANOVA analysis followed by Tukey’s multiple-comparison test, F = 10.64) for, F = 8.674.WB assaysand quantification results showed that the levels of O-GlcNAcylation, Ogt, Ogasignificantly decreased, but the level of APPsignificantly increased in AD astrocytes compared with Ctrl astrocytes. n = 4 independent experiments. Values represent mean ± SEM; *p < 0.05, **p < 0.01, ***p < 0.001; unpaired Student’s t-test.IP-WBshowed the reduced levels of O-GlcNAcylationand Ogt, but the levels of p65and GSK3βwere not affected in the hippocampus of Ctrl and AD mice. n = 4 mice per group. Values represent mean ± SEM; *p < 0.05, **p < 0.01, ***p < 0.001; unpaired Student’s t-test.Quantification results of IP inshowed that the decreased O-GlcNAcylation on p65, the decreased interaction between p65 and Ogt, and the increased interaction between p65 and GSK3βin the hippocampus of AD mice compared to Ctrl mcie. n = 4 mice per group. Values represent mean ± SEM; *p < 0.05, **p < 0.01, ***p < 0.001; unpaired Student’s t-test. [file 12974_2023_2824_MOESM9_ESM.tiff]
