# Supplementary material for: Ogt-mediated O-GlcNAcylation inhibits astrocytes activation through modulating NF-κB signaling pathway
Source: J Neuroinflammation. 2023 Jun 22;20:146. doi: 10.1186/s12974-023-02824-8 (PMC10286367; doi:10.1186/s12974-023-02824-8)
Supplement: Supplementary file 10 — Additional file 10: Figure S10. The original images of all western blot assays. [file 12974_2023_2824_MOESM10_ESM.pdf]

Figure 1c

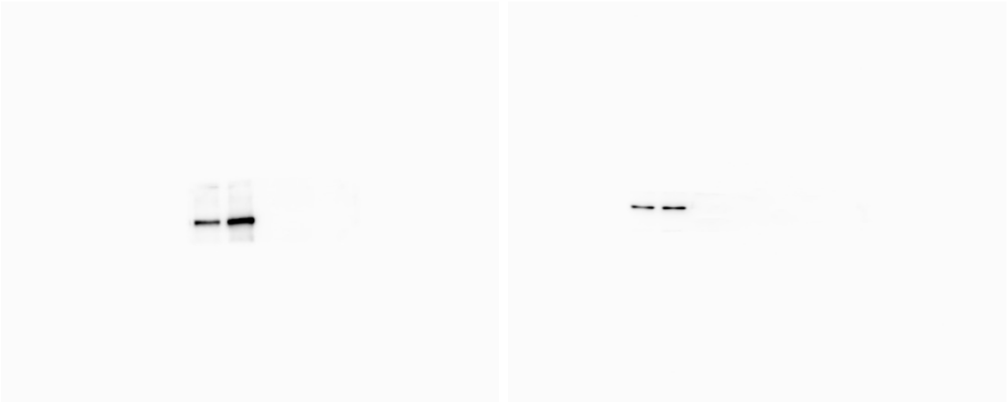

GFAP Gapdh

Figure 1q

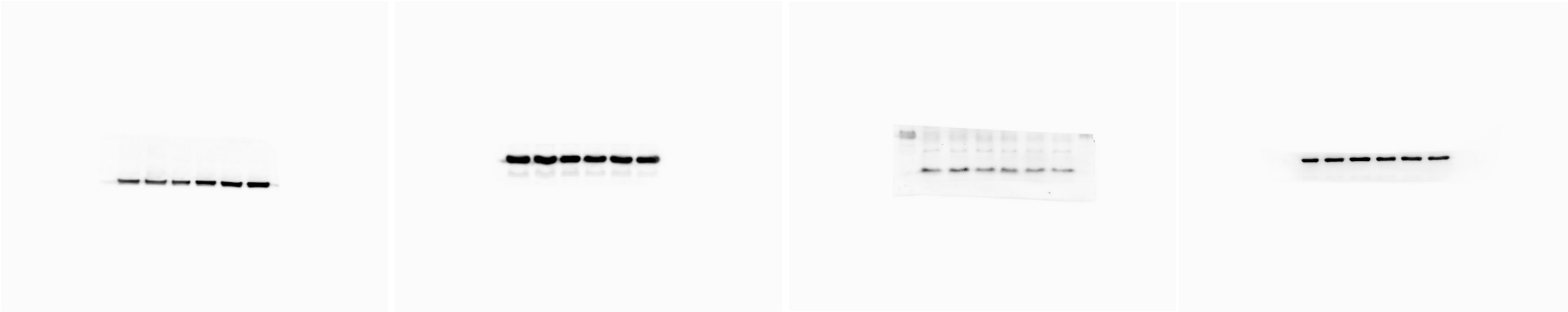

GFAP Gapdh Iba1 Gapdh

Figure 2d

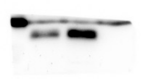

IL-1β

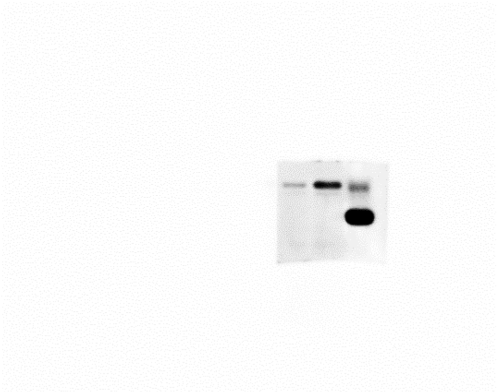

TNF-α

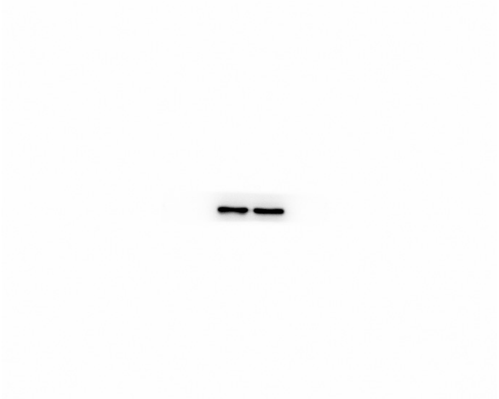

Gapdh

Figure 2j

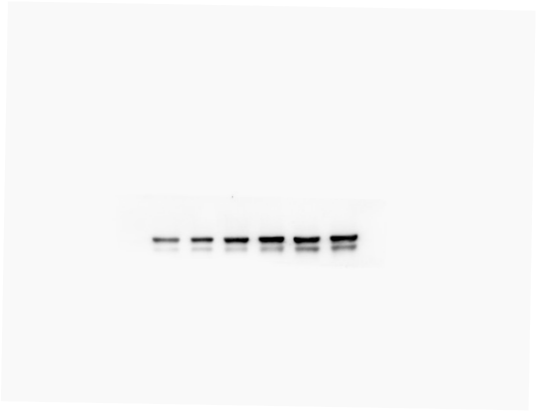

IL-1β

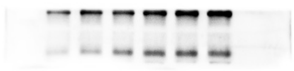

TNF-α

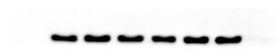

Gapdh

Figure 2o

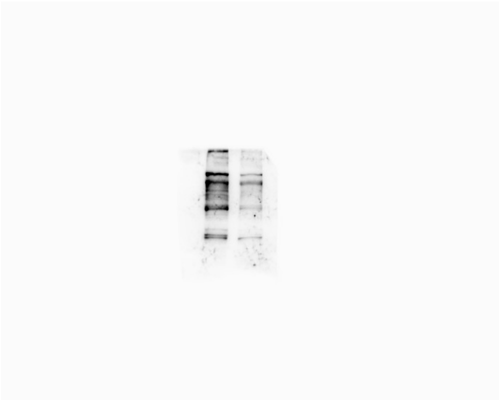

O-GlcNAc

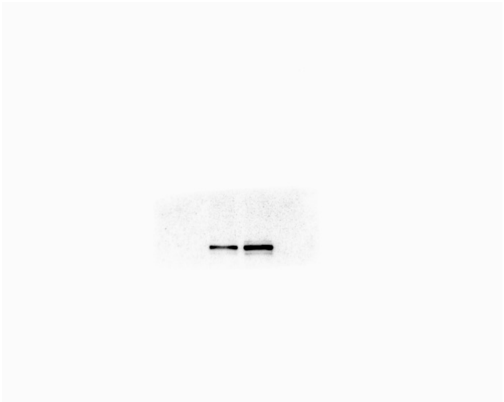

GFAP

Gapdh

Figure 2s

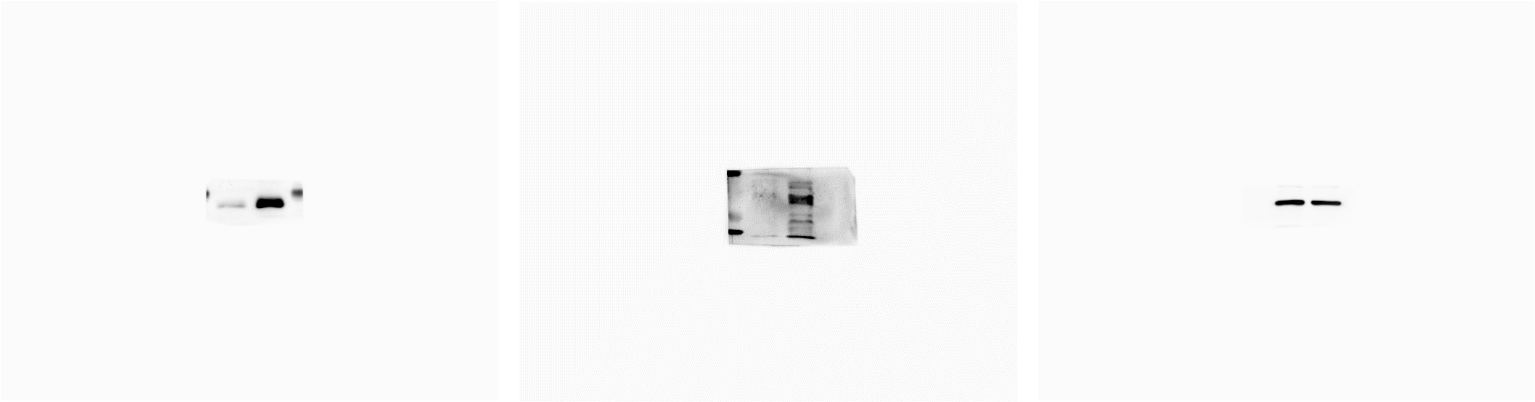

IL-1β TNF-α Gapdh

Figure 4b

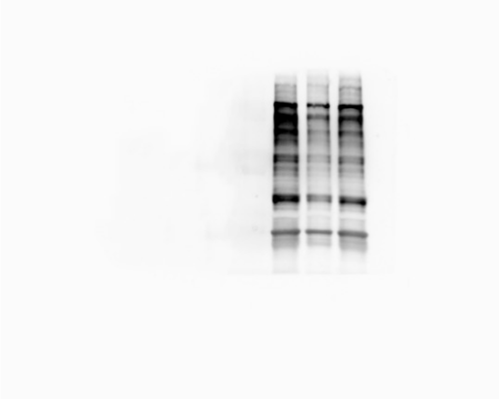

O-GlcNAc

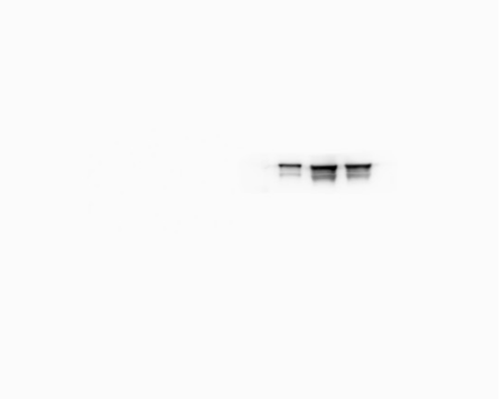

GFAP

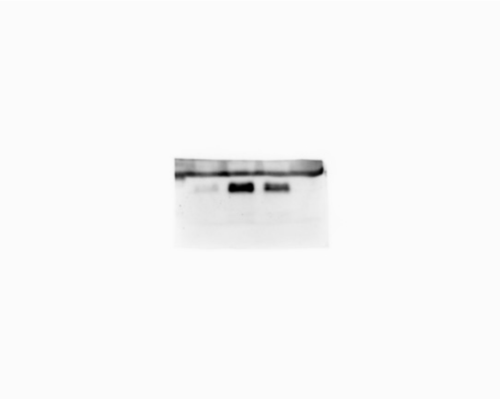

IL-1β

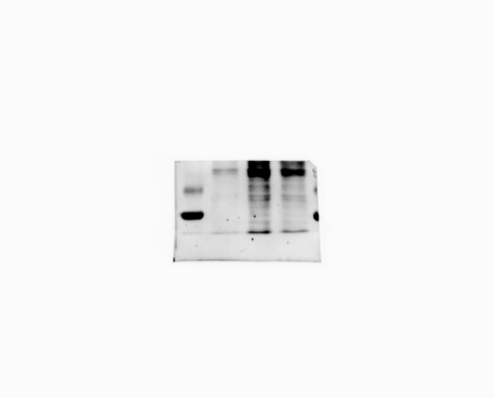

TNF-α

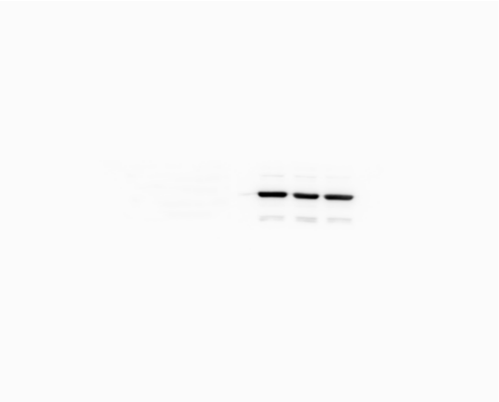

Gapdh

Figure 4m

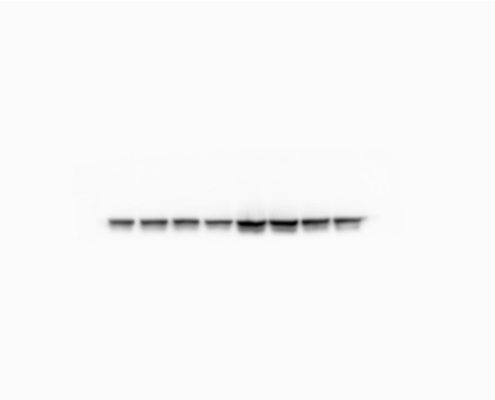

GFAP

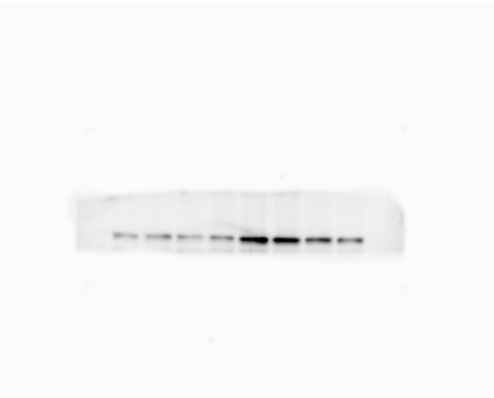

IL-1β

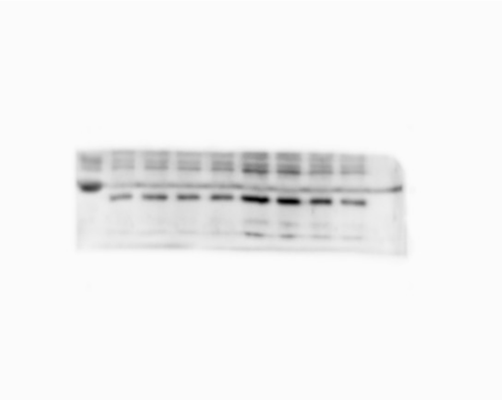

TNF-α

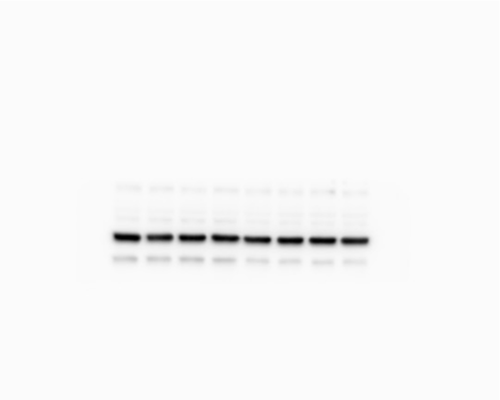

Gapdh

Figure 6a

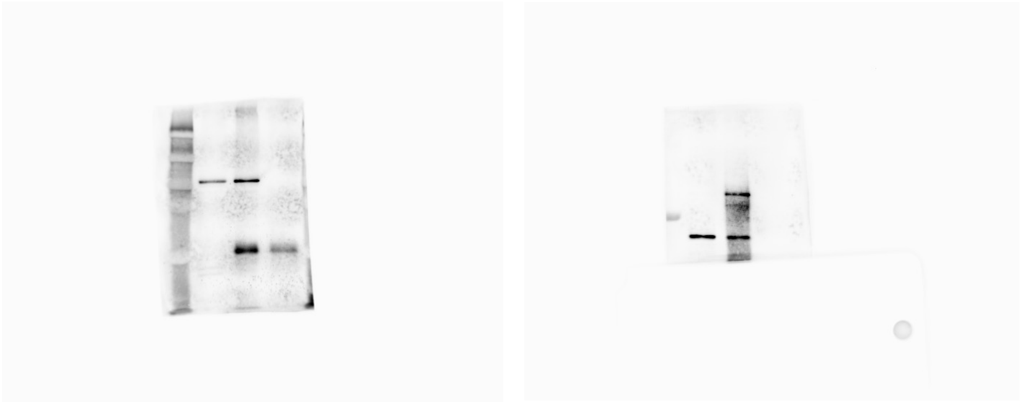

Ogt p65

Figure 6b

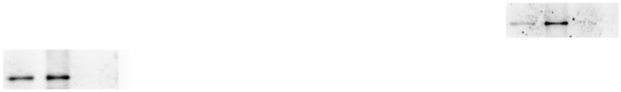

p65 Ogt

Figure 6c

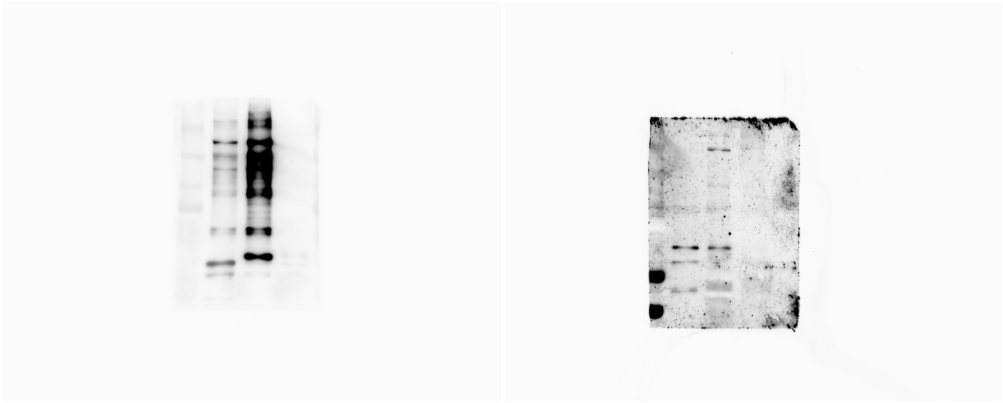

O-GlcNAc p65

Figure 6d

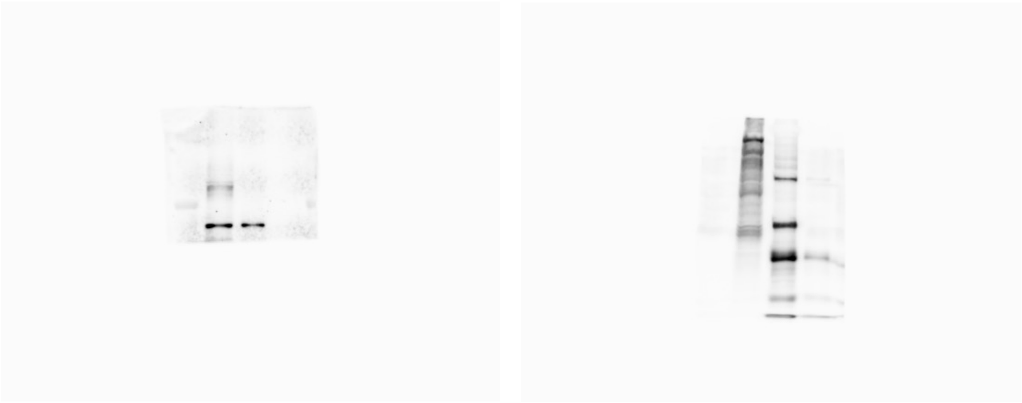

p65 O-GlcNAc

Figure 6e

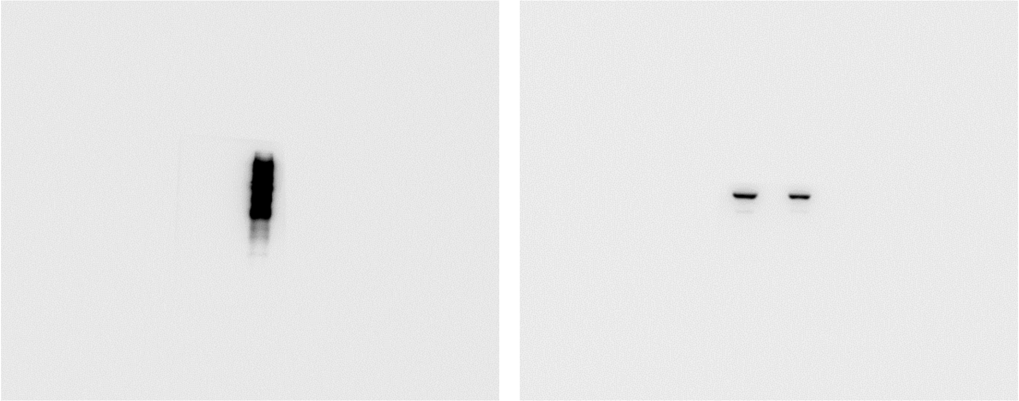

Streptavidin-HRP p65

Figure 6f

Input

Ogt

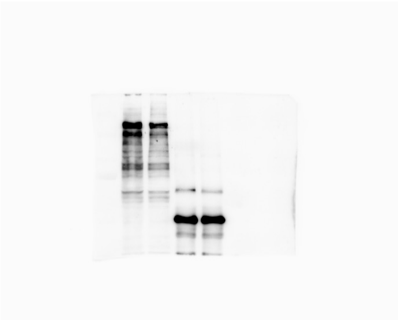

O-GlcNAc

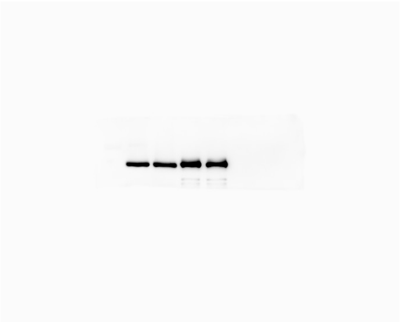

HA (p65)

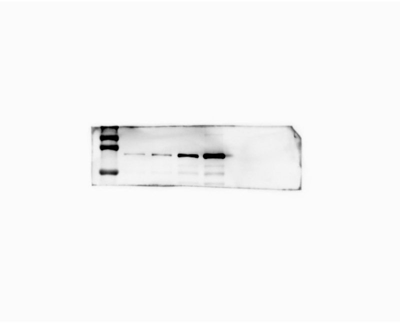

p-p65

Gapdh

IP

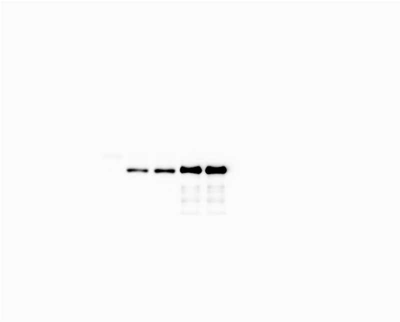

HA (p65)

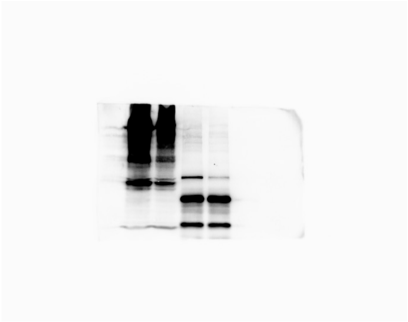

O-GlcNAc

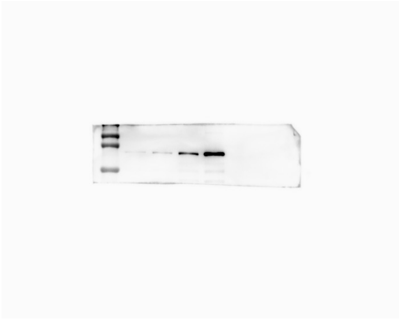

p-p65

Figure 6i

Input

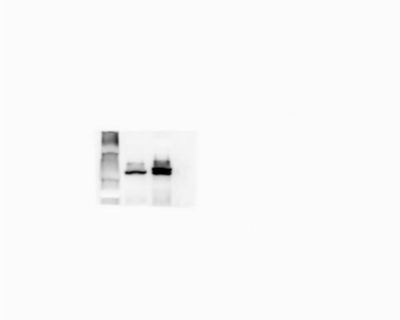

Ogt

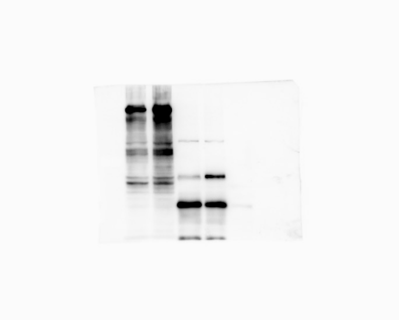

O-GlcNAc

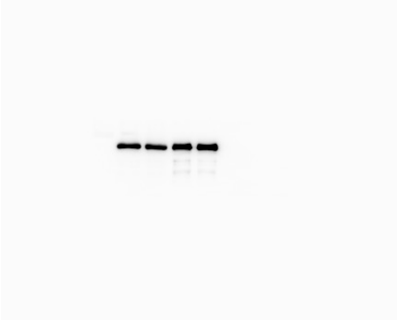

HA (p65)

p-p65

Gapdh

IP

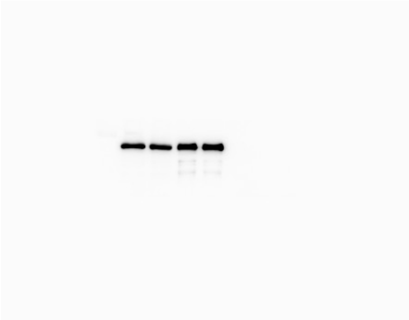

HA (p65)

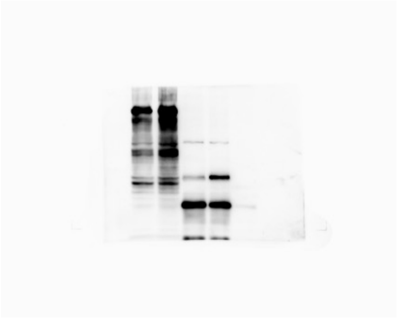

O-GlcNAc

p-p65

Figure 6I

Input

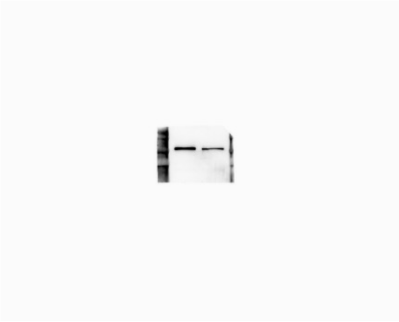

Ogt

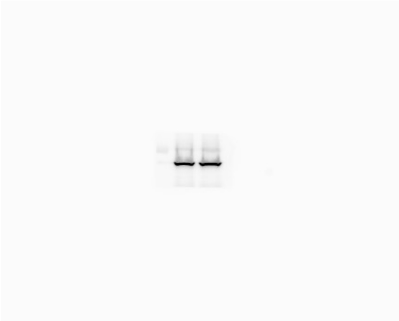

HA (p65)

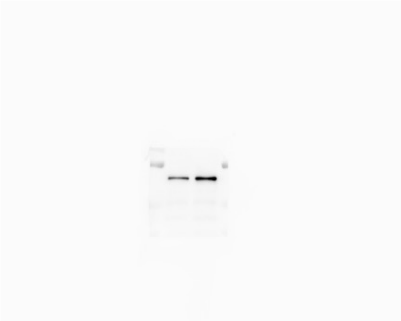

p-p65

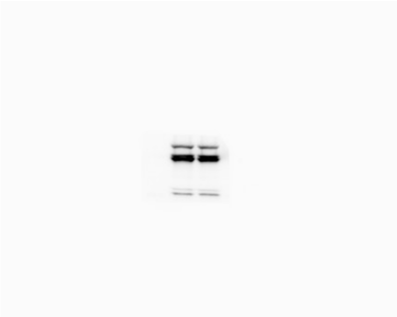

Gsk3β

Gapdh

HA(p65) co-IP

HA (p65)

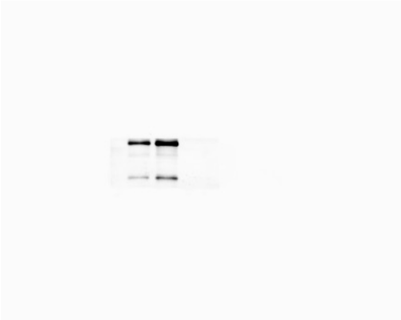

Gsk3β

Gsk3β co-IP

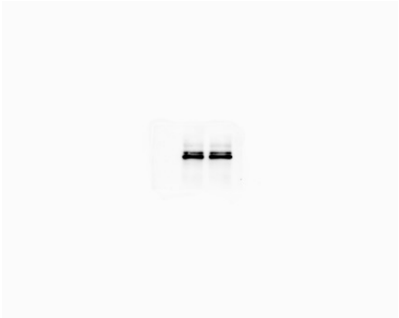

Gsk3β

HA (p65)

Figure 6o

Input

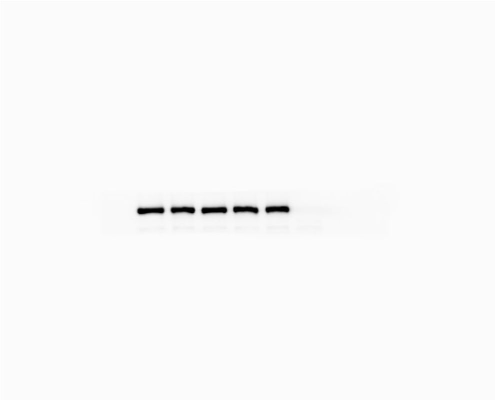

HA (p65)

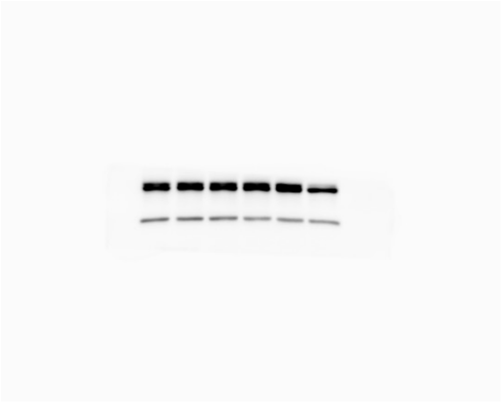

Gsk3β

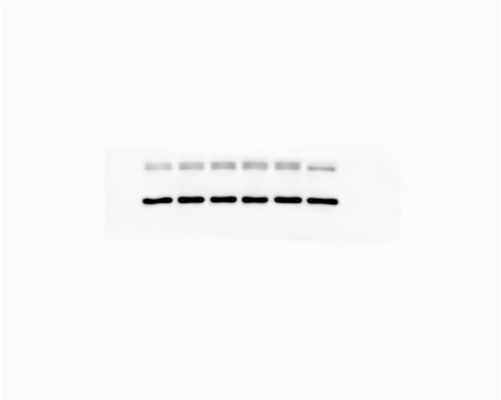

Gapdh

HA(p65) Co-IP

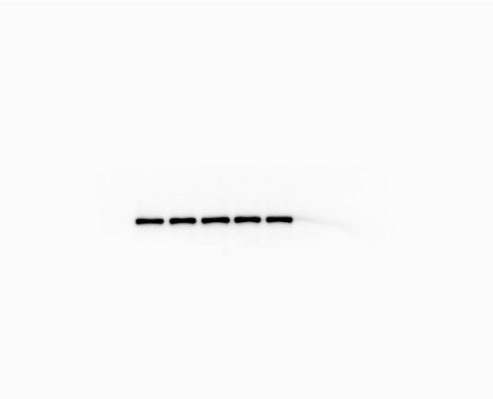

HA(p65)

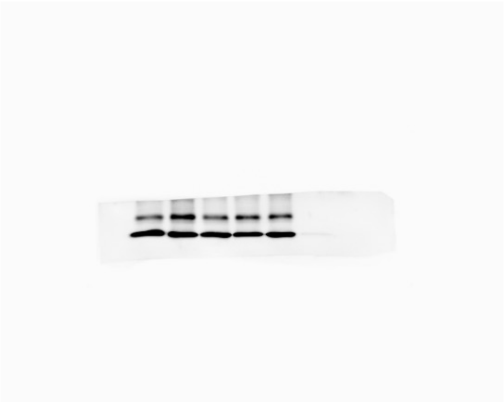

Gsk3β

Gsk3β Co-IP

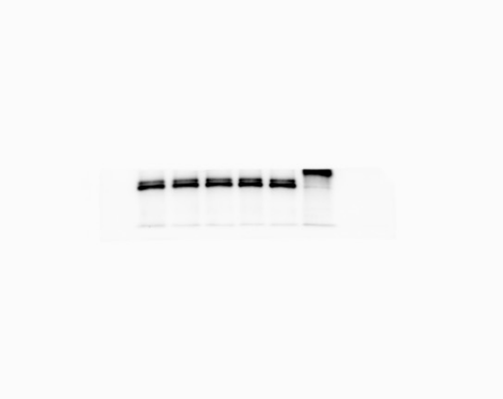

Gsk3β

HA(p65)

Figure 7e

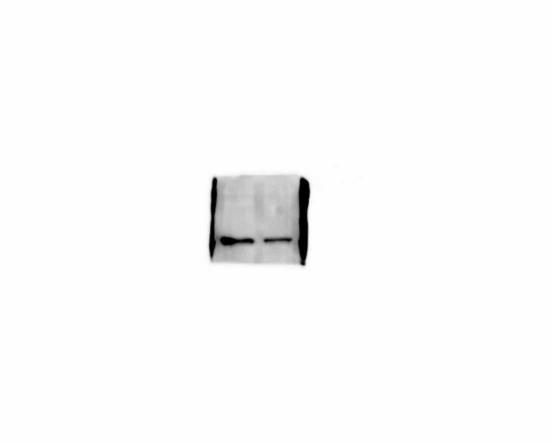

Ogt

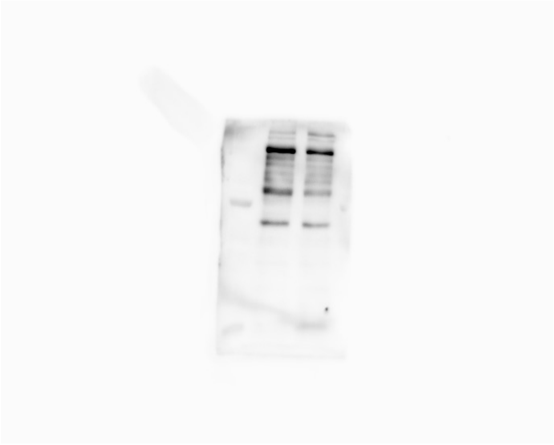

O-GlcNAc

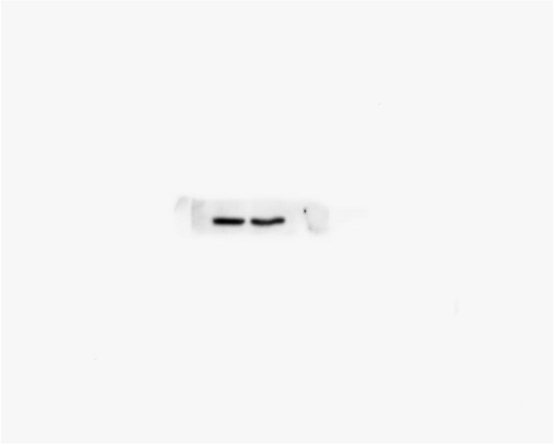

Actin

Figure 7h

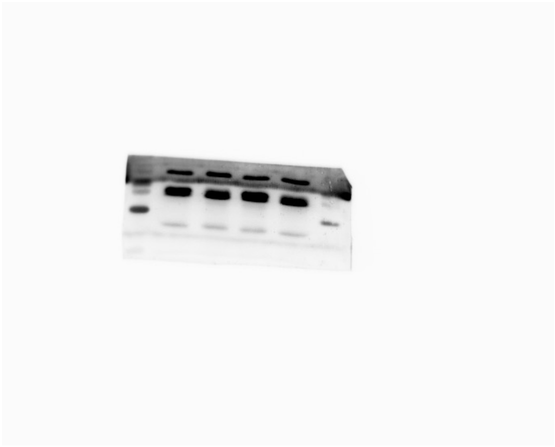

IL-1β

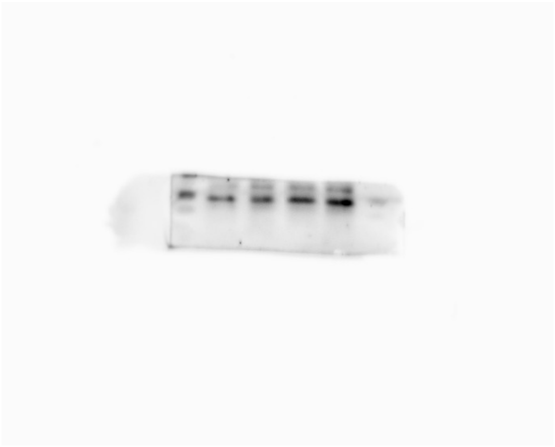

TNF-α

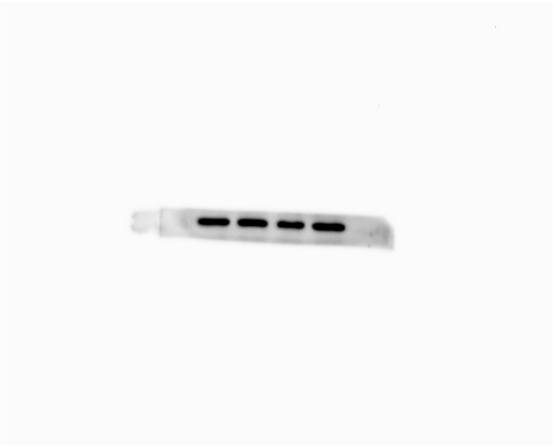

Actin

Figure 7k

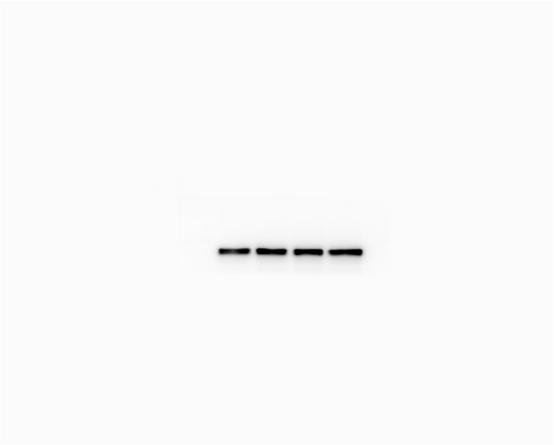

p65

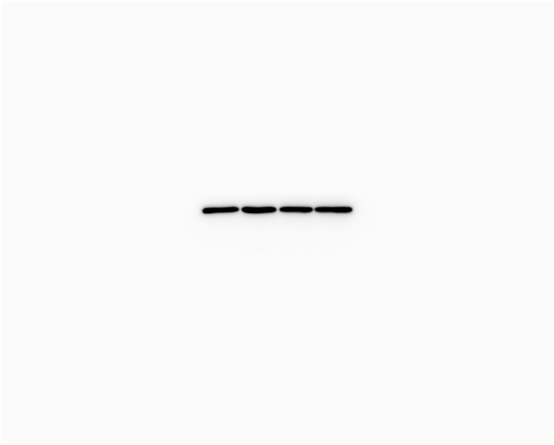

Gapdh

Figure 7m

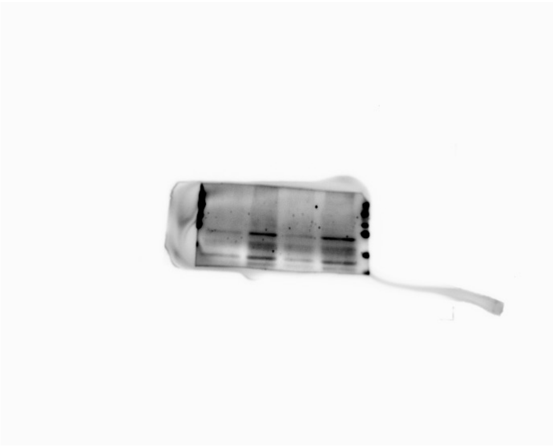

pp65

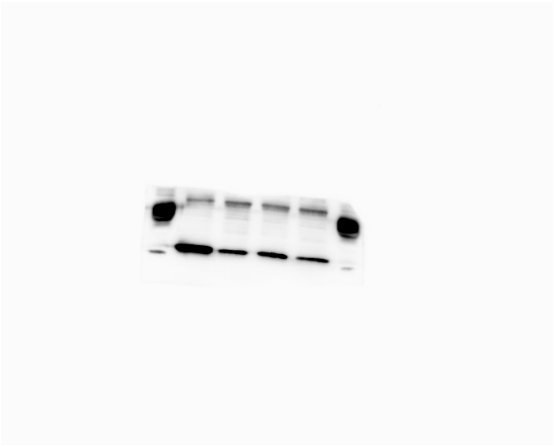

H3

Figure 8e

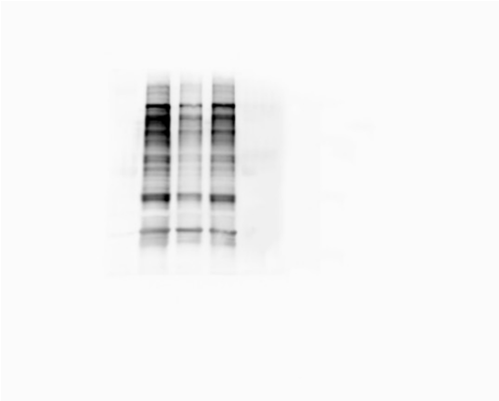

O-GlcNAc

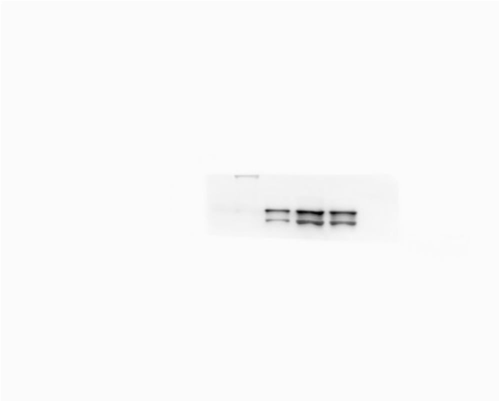

GFAP

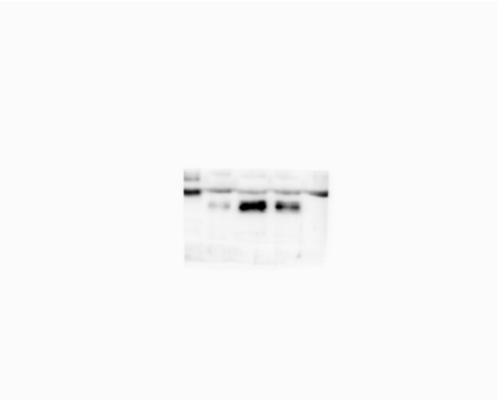

IL-1β

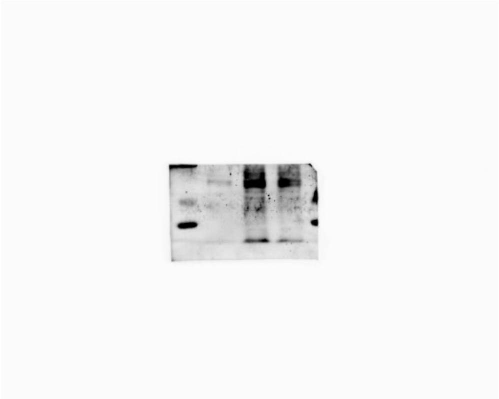

TNF-α

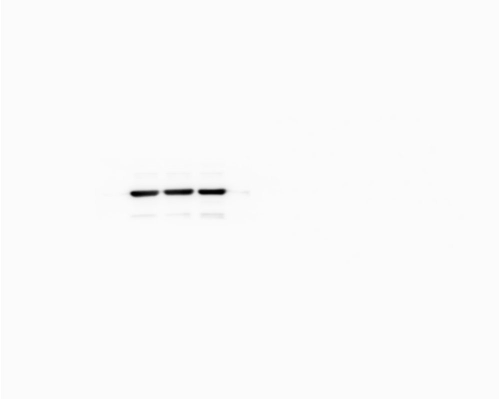

Gapdh

Figure 8o

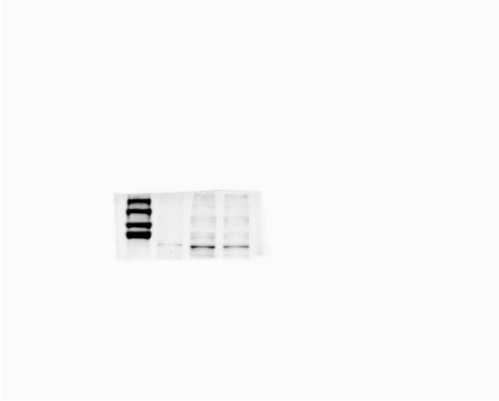
p-p65

p65

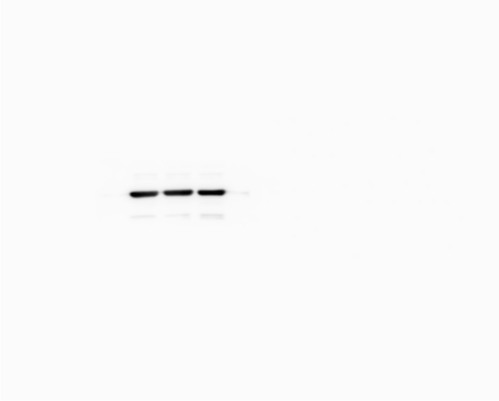
Gapdh

Figure 8r

p65

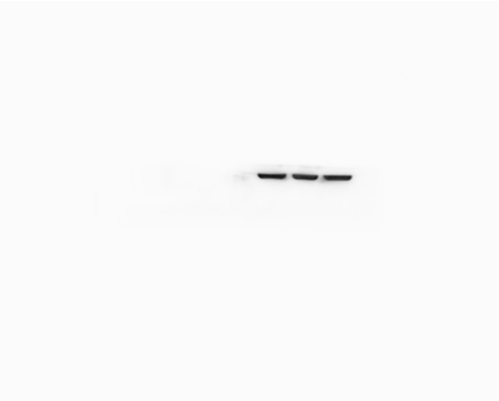
Gapdh

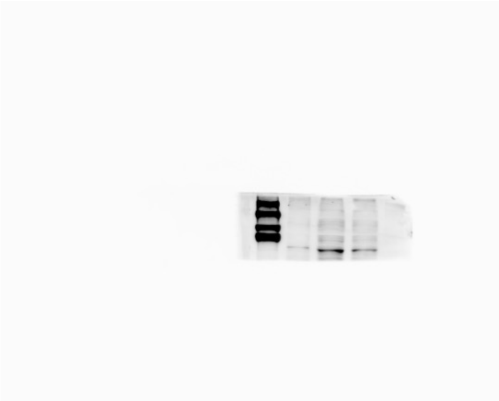
p-p65

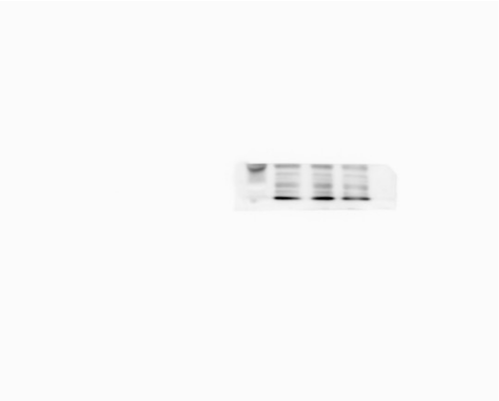
Histone-H3

Figure S1c

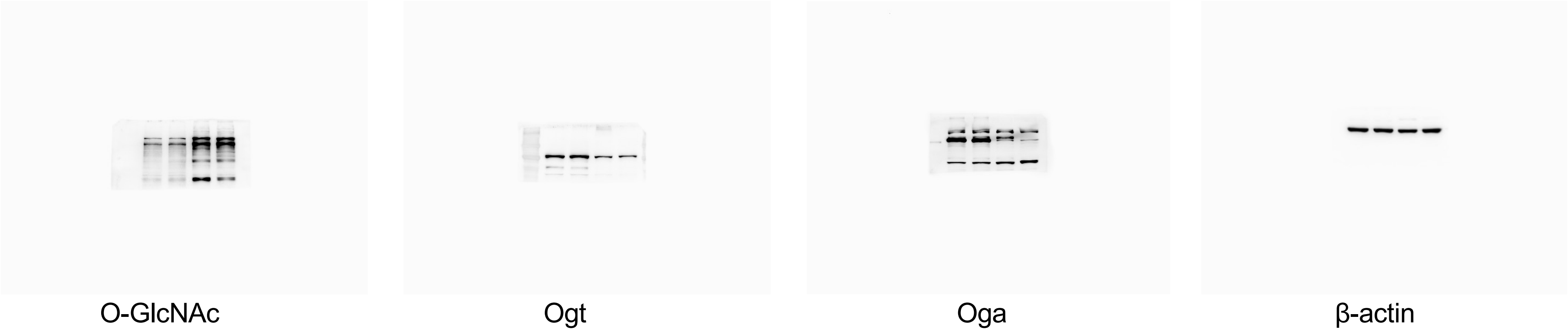

Figure S1j

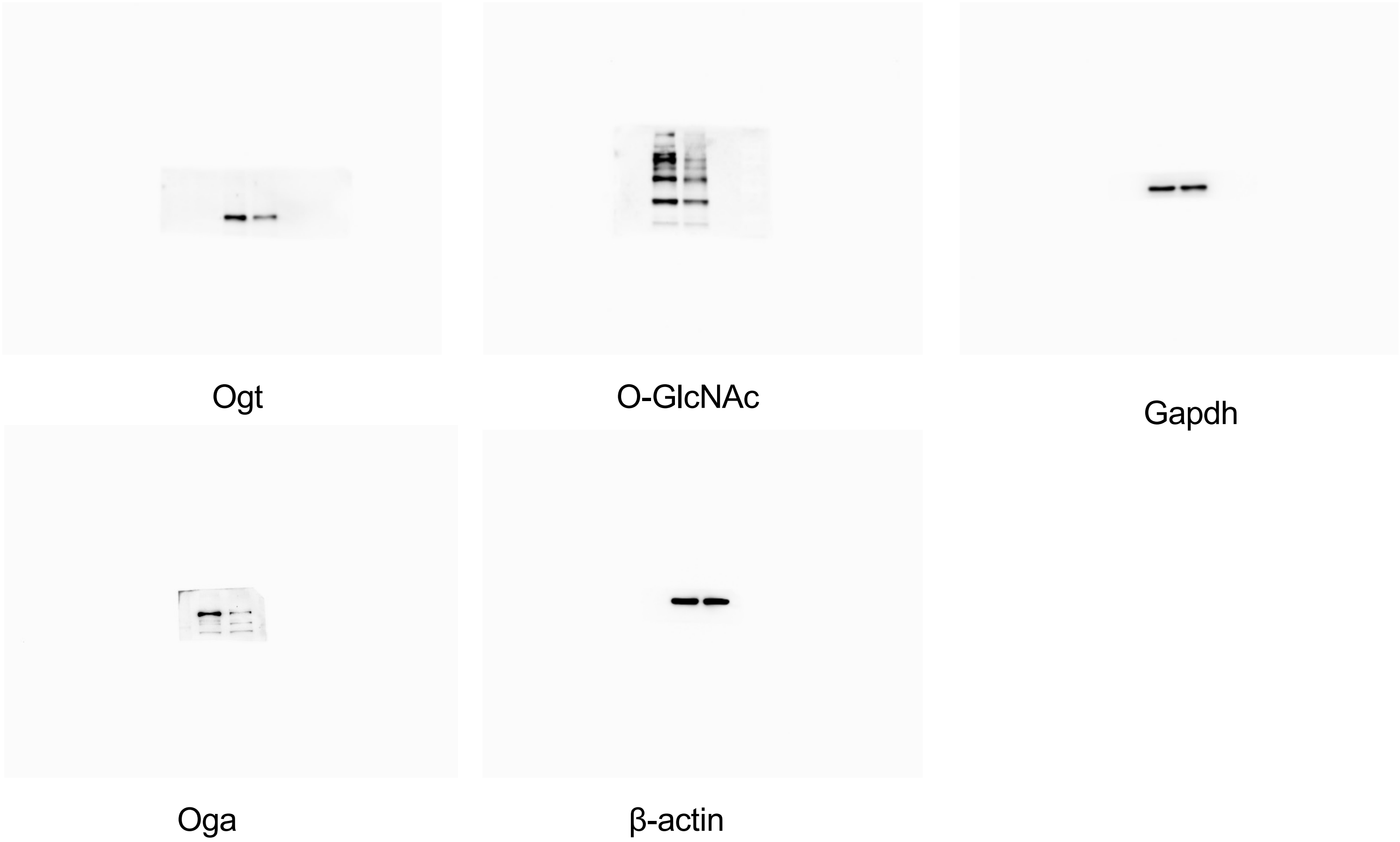

Figure S3i

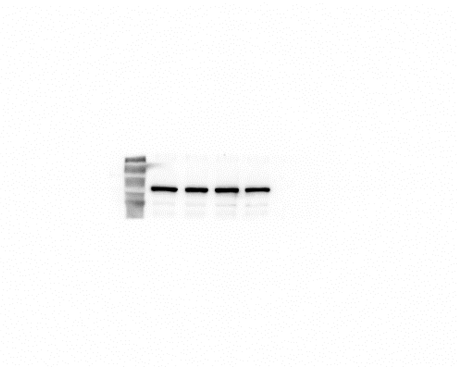

Ogt

Gapdh

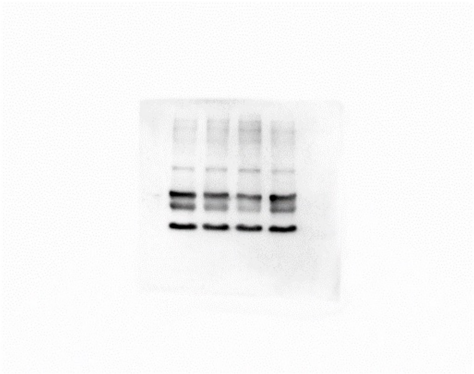

O-GlcNAc

Gapdh

Figure S4i

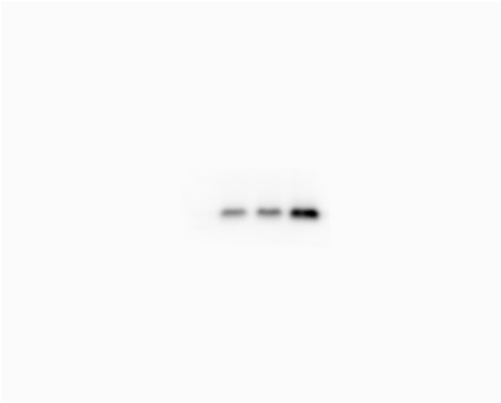

aCaspase3

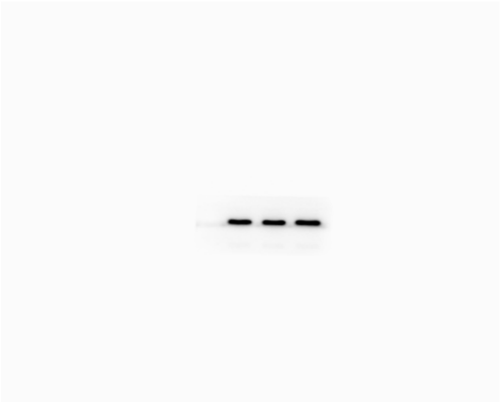

Gapdh

Figure S7b

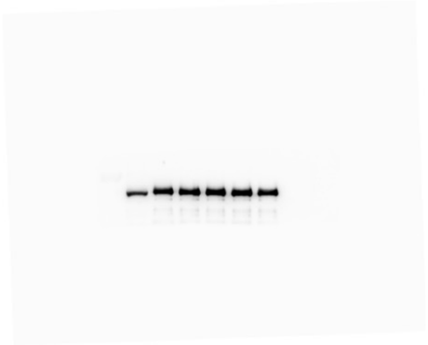

HA (p65)

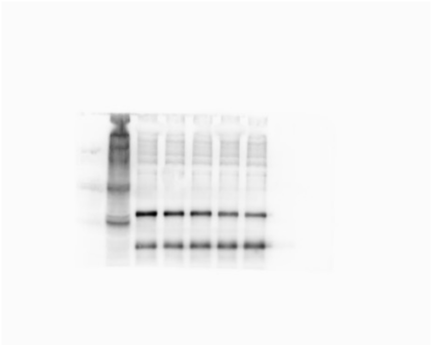

O-GlcNAc

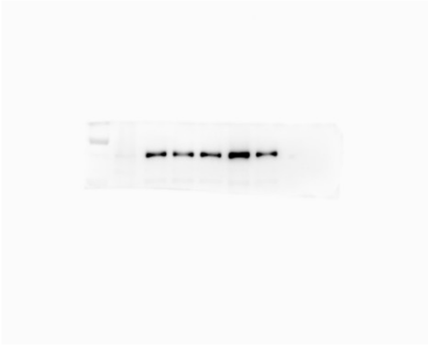

p-p65

Figure S7e

Input

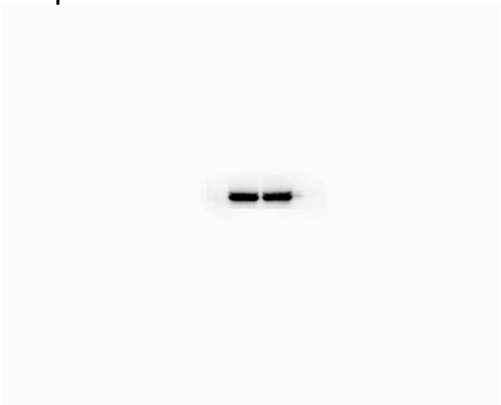

Gsk3β

p-p65

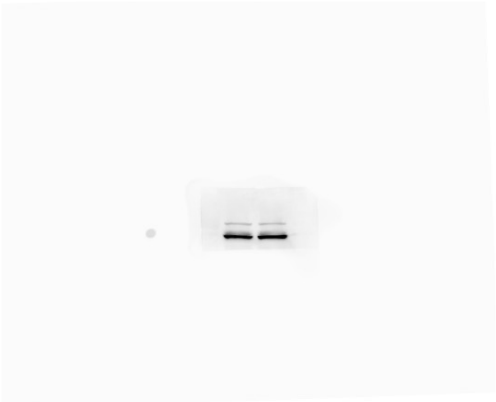

p65

Gapdh

p65-co IP

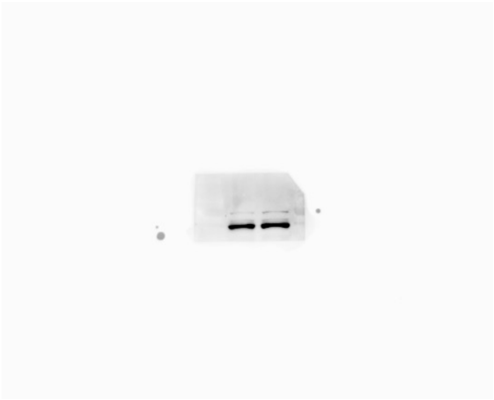

p65

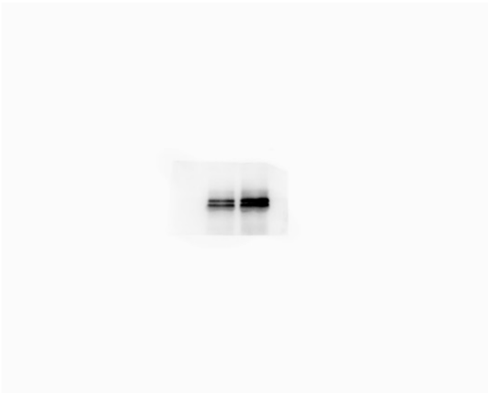

Gsk3β

Gsk3β-co IP

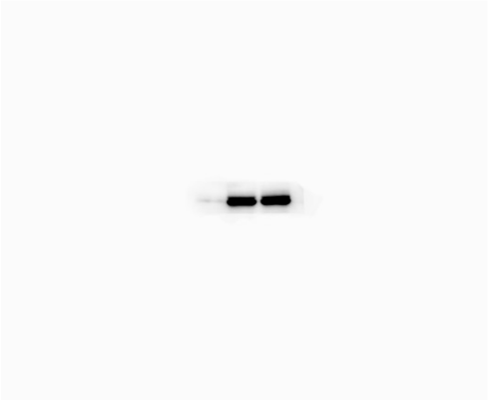

Gsk3β

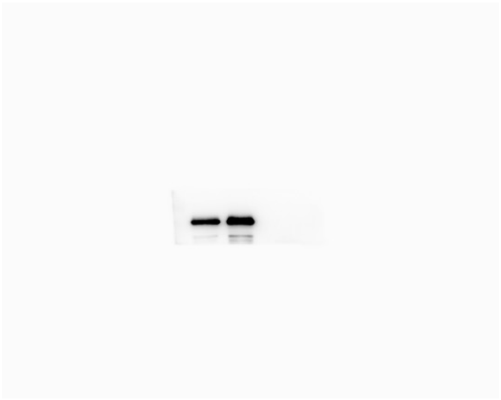

p65

Figure S7j

Input

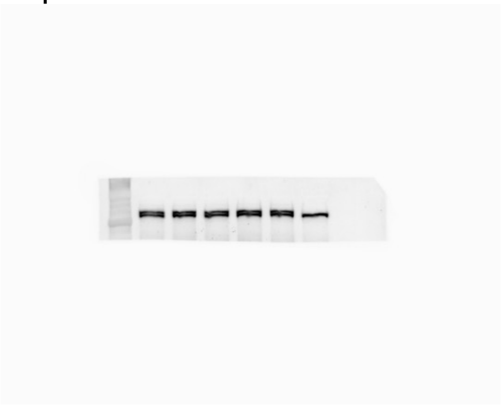

Ogt

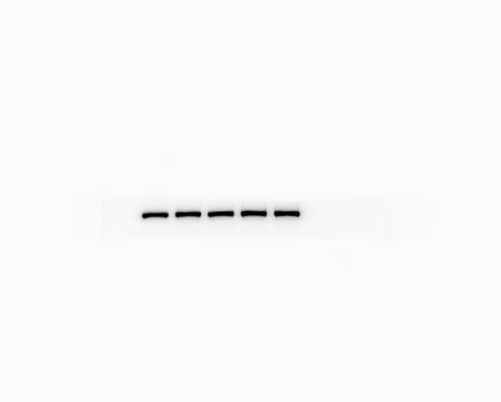

HA (p65)

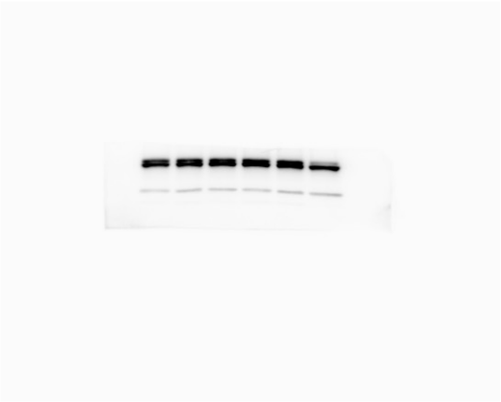

Gsk3β

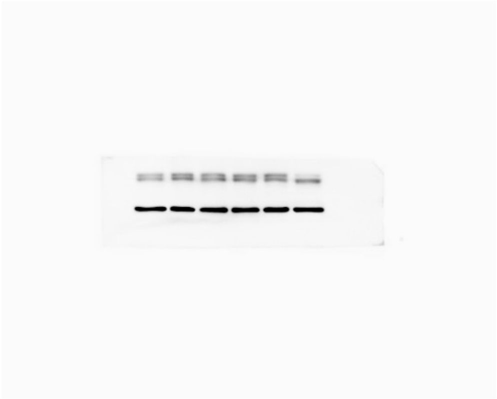

Gapdh

Flag(Ogt) co-IP

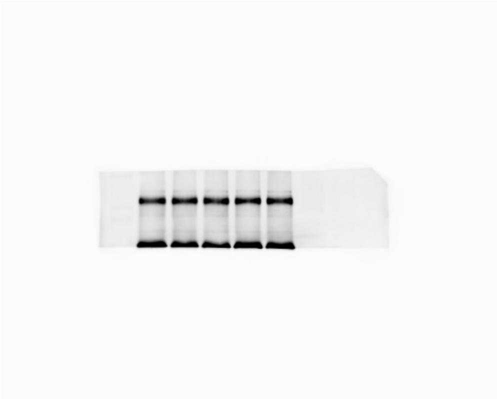

Flag(Ogt)

Gsk3β

Gsk3β co-IP

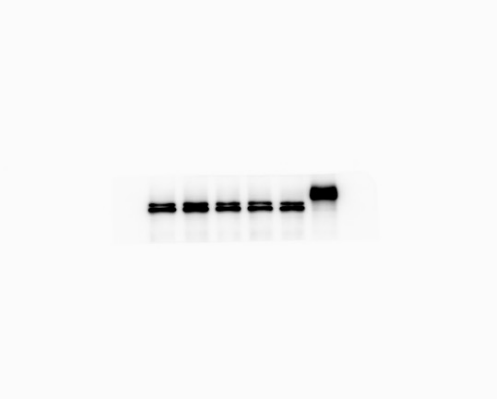

Gsk3β

Flag(Ogt)

Figure S8c

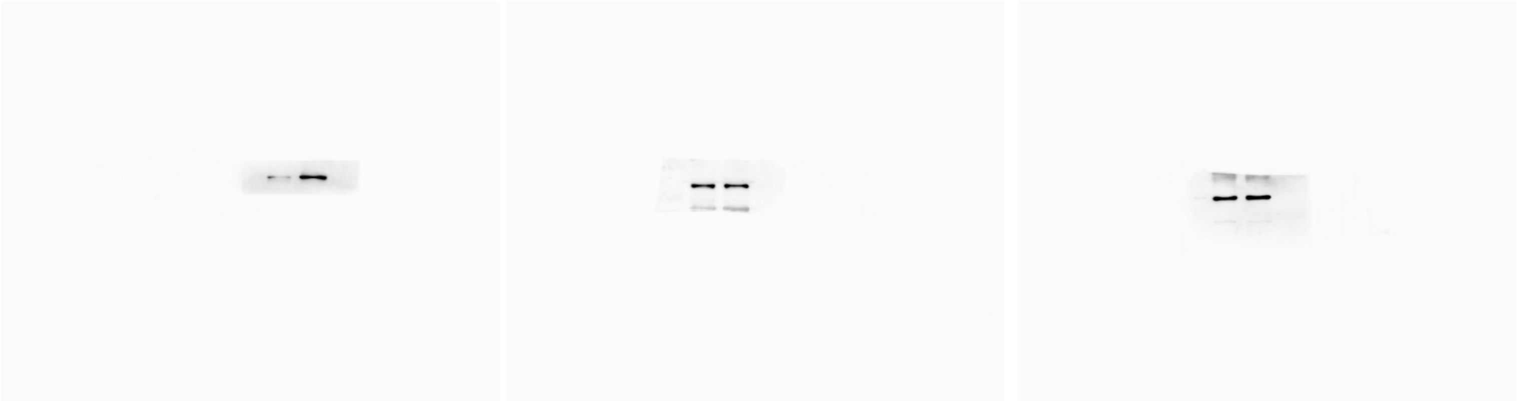

p-p65 p65 Gapdh

Figure S8f

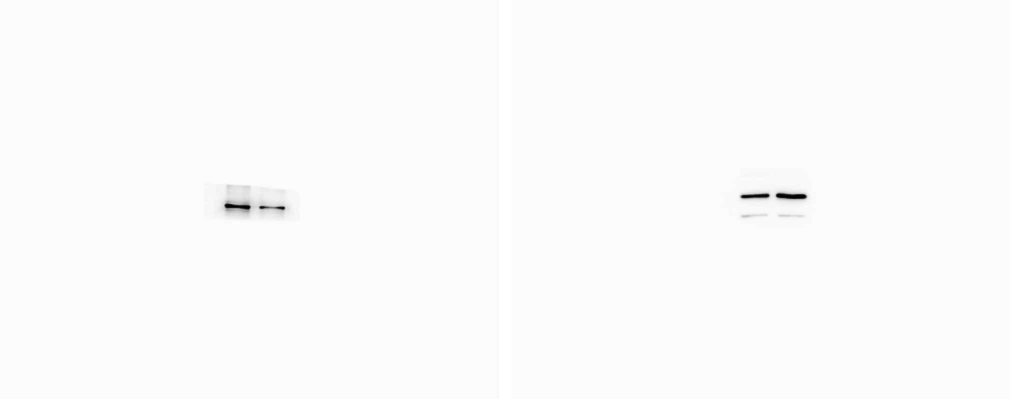

p65 Gapdh

Figure S8h

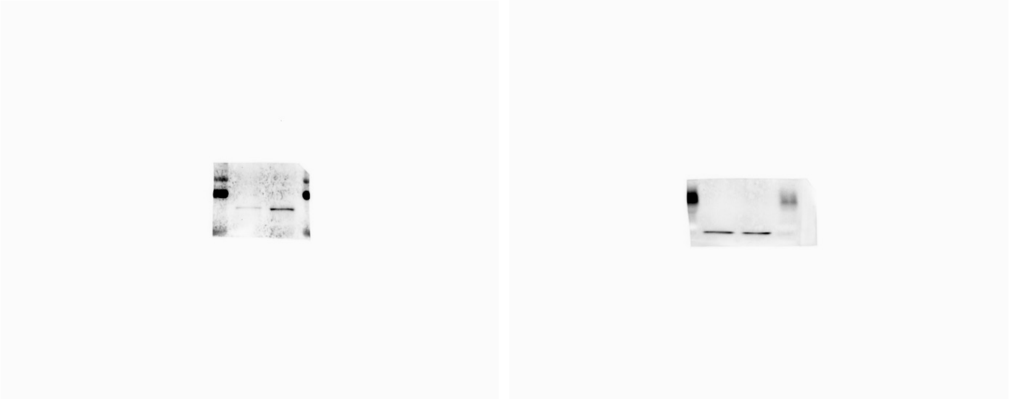

p-p65 Histone-H3

Figure S9e

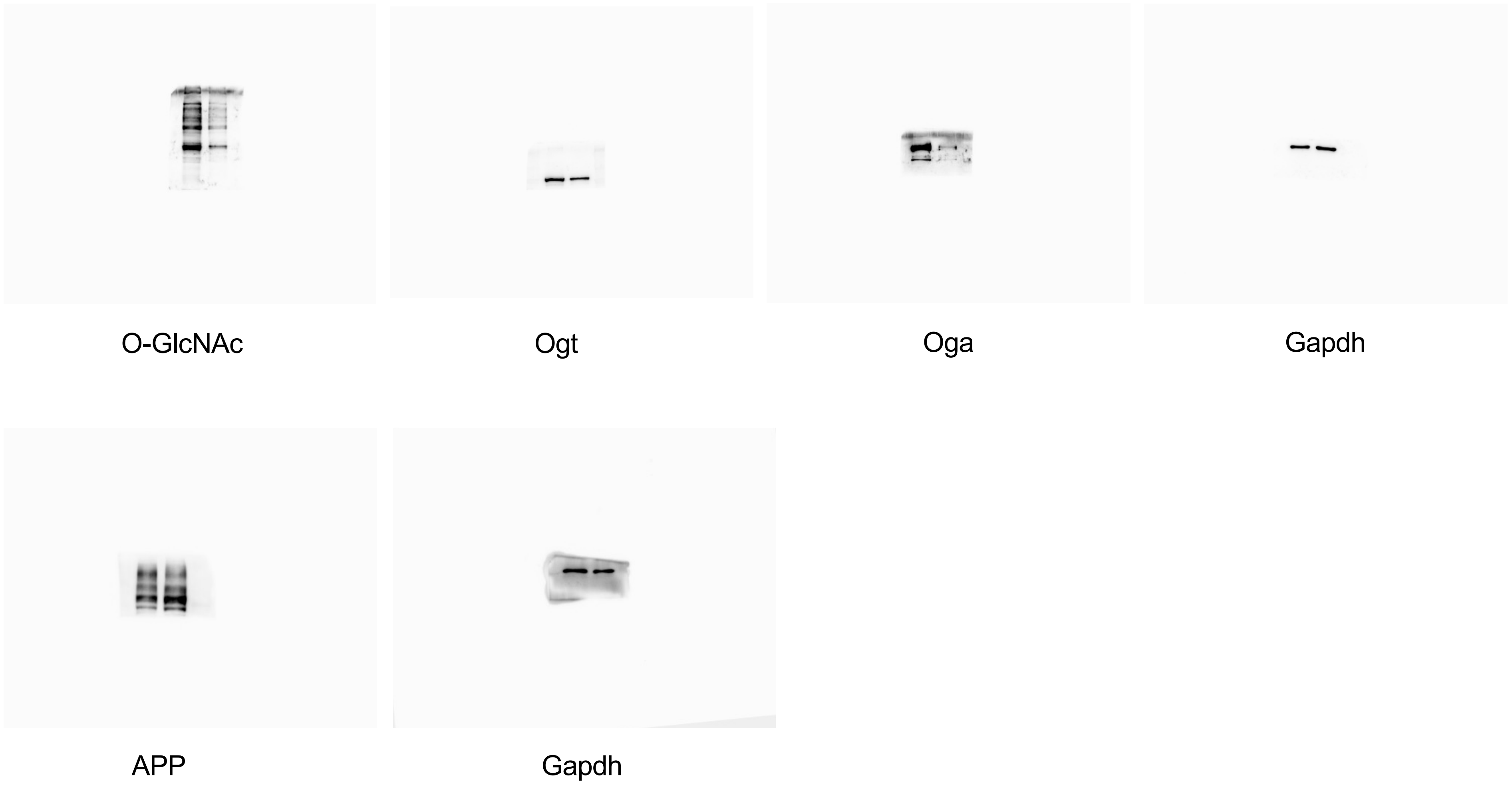

Figure S9j

Input

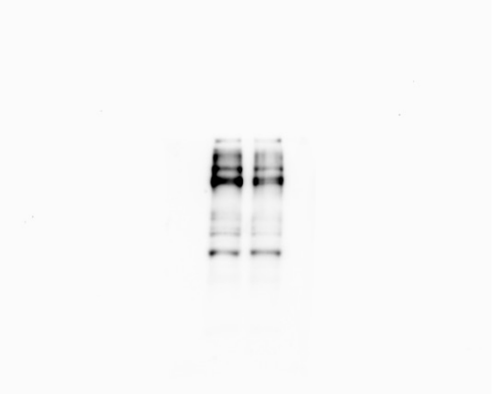

O-GlcNAc

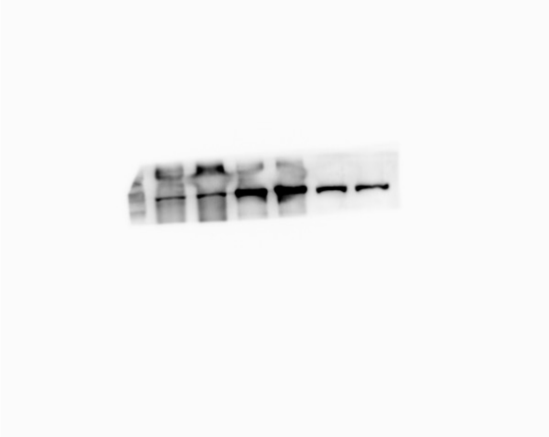

Ogt

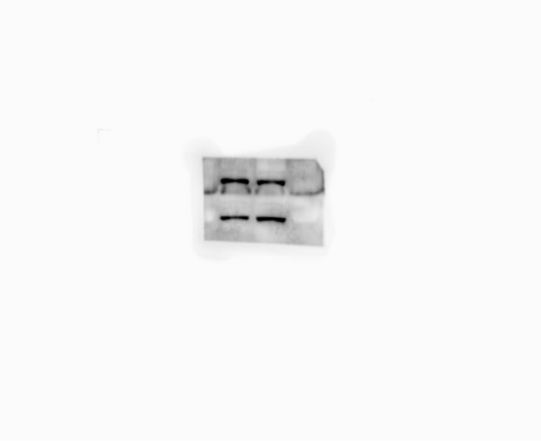

p65

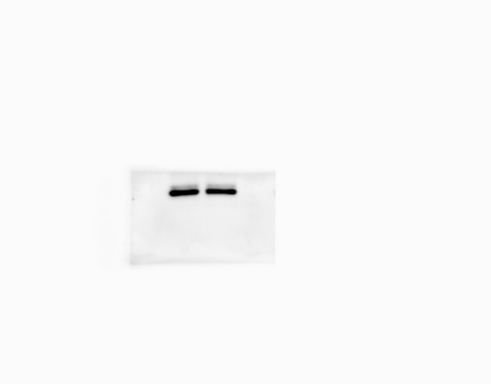

GSK3β

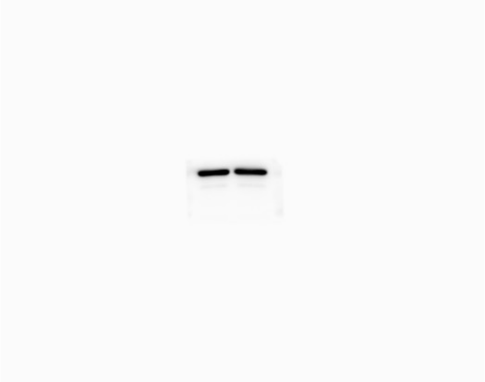

Gapdh

Figure S9j

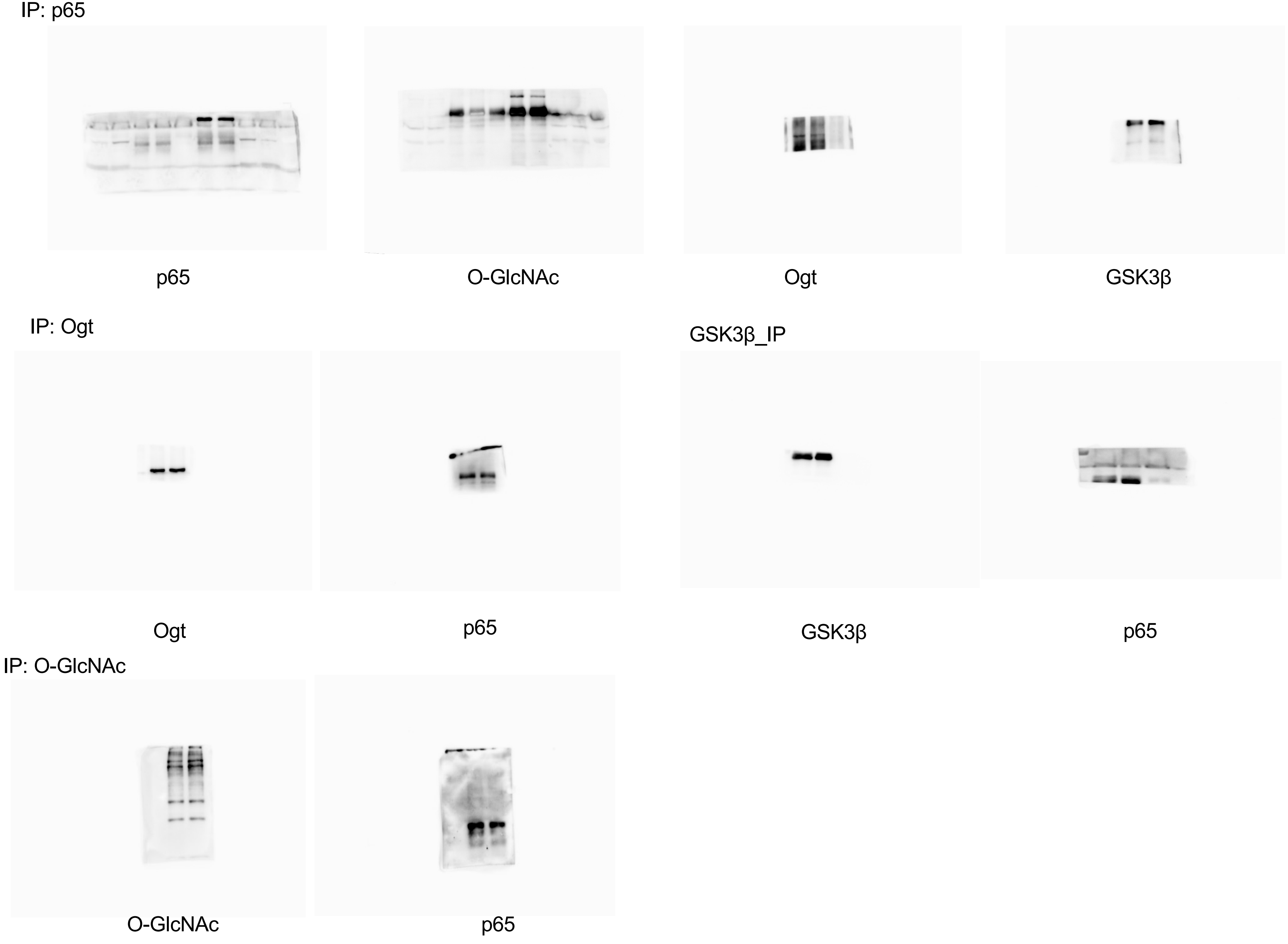
IP: p65
p65
O-GlcNAc
Ogt
GSK3β
IP: Ogt
GSK3β_IP
Ogt
p65
GSK3β
p65
IP: O-GlcNAc
O-GlcNAc
p65
